# *In vivo* modeling of lethal congenital contracture syndrome 1 suggests pathomechanisms in cellular stress responses

**DOI:** 10.1111/febs.70195

**Published:** 2025-07-17

**Authors:** Tomáš Zárybnický, Sonja Lindfors, Saana Metso, Julia Koivula, Zoltan Szabo, Rasmus Valtonen, Mikko Tulppo, Johanna Magga, Samu Saarimäki, Sonja Bläuer, Ilkka Miinalainen, Risto Kerkelä, Petteri. T. Piepponen, Vootele Voikar, Juho Väänänen, Riikka Kivelä, Bhagwan Yadav, Hanna Lindgren, Pirkko Mattila, Fu‐Ping Zhang, Petra Sipilä, Reetta Hinttala, Satu Kuure

**Affiliations:** ^1^ Helsinki Institute of Life Science University of Helsinki Finland; ^2^ Stem Cells and Metabolism Research Program Unit, Faculty of Medicine University of Helsinki Finland; ^3^ Research Unit of Biomedicine and Internal Medicine, Department of Pharmacology and Toxicology University of Oulu Finland; ^4^ Medical Research Center Oulu Oulu University Hospital and University of Oulu Finland; ^5^ Biocenter Oulu University of Oulu Finland; ^6^ Division of Pharmacology & Pharmacotherapy, Faculty of Pharmacy University of Helsinki Finland; ^7^ Laboratory Animal Centre, Helsinki Institute of Life Science (HiLIFE) University of Helsinki Finland; ^8^ Biomedicum Functional Genomics Unit, Helsinki Institute of Life Science and Applied Tumors Genomics Research Program, Faculty of Medicine University of Helsinki Finland; ^9^ Wihuri Research Institute Helsinki Finland; ^10^ Faculty of Sport and Health Sciences University of Jyväskylä Finland; ^11^ Institute for Molecular Medicine Finland (FIMM) University of Helsinki Finland; ^12^ EATRIS ERIC‐European Infrastructure for Translational Medicine Amsterdam The Netherlands; ^13^ Institute of Biomedicine, Research Centre for Integrative Physiology and Pharmacology, Turku Center for Disease Modeling University of Turku Finland; ^14^ Research Unit of Clinical Medicine, Medical Research Center University of Oulu, Oulu University Hospital Finland; ^15^ Laboratory Animal Centre University of Helsinki Finland

**Keywords:** embryonic development, LCCS1, neural crest, stress response, sympathetic neurons

## Abstract

The mRNA export factor GLE1 protein plays critical yet enigmatic functions in RNA processing and has been linked with multiple developmental disorders, including lethal congenital contracture syndrome 1 (LCCS1). Using *in vivo* genetic engineering to study disturbed GLE1 functions under physiological conditions, we demonstrate that total inactivation of GLE1 results in disorganization of the blastocyst inner cell mass and early embryonic lethality due to defects in lineage specification. In contrast, the knock‐in mice genocopying the LCCS1‐associated *GLE1*_FinMajor_ variant (*Gle1* ^PFQ/PFQ^) survive the prenatal period but die suddenly at midadulthood. *Gle1* ^PFQ/PFQ^ mice present an irregular count and distribution of spinal motor neurons as well as impaired development of neural crest‐derived tissues, as demonstrated by defects in the sympathetic innervation of heart ventricles, irregularities in the paravertebral sympathetic ganglia volume, and decreased adrenal chromaffin cell counts. Unlike previously reported for yeast and HeLa cells, analysis of the molecular consequences of the *GLE1*_FinMajor_ variant identified normal poly(A) + RNA distribution in *Gle1* ^PFQ/PFQ^ cells; however, cells were impaired in RNA and protein synthesis and simultaneously showed severely disturbed formation of G3BP stress granule assembly factor 1 (G3BP1)‐positive stress granules. Intriguingly, stressed *Gle1* ^PFQ/PFQ^ cells show microRNA profiles indicative of impaired transcription, protein metabolism, nervous system development, and axon guidance, further corroborating our functional findings. Our results show the necessity of functional GLE1 for life and indicate that LCCS1 etiology is a result of the pathogenic *GLE1*_FinMajor_ variant impinging differentiation of neural crest derivatives and leading to complex multiorgan defects.

## Graphical abstract

GLE1 plays a direct role mRNA regulation, translation, and formation of cytosolic stress granules. GLE1 dysfunction has been associated with devastating diseases, including lethal congenital contracture syndrome 1(LCCS1), a rare disorder enriched in isolated populations. *Gle1*‐knockout mice fail to segregate cell lineages at the blastocyst stage, resulting in very early embryonic lethality. *Gle1* knock‐in mice harboring a pathogenic variant that causes LCCS1 show both known and novel innervation defects, supportive of the multiorgan pathology seen in human fetuses carrying the same mutation. Mutant *Gle1* knock‐in cells exhibit aberrant miRNA profiles which, together with abnormal cellular stress responses, suggest wider pathomechanisms in RNA metabolism. This work shows how the pathogenic *GLE1* variant may lead to complex multiorgan defects in LCSS1.

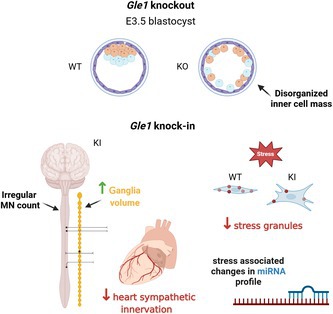

**Abbreviations**
C: cervical
ECG: electrocardiography
EdU: 5‐ethynyl‐2′‐deoxyuridine
EU: ethynyl uridin
FISH: fluorescent *in situ* hybridization
GO: gene ontology
HRV: heart rate variability
ISR: integrated stress response
KI: knock‐in
KO: knockout
L: lumbar
LCCS1: lethal congenital contracture syndrome 1
MEFs: mouse embryonic fibroblasts
MN: motoneuron
MST: median survival time
NMJ: neuromuscular junctions
OP‐puro: O‐propargyl‐puromycin
polyA: poly‐adenylated
RNA‐seq: RNA‐sequencing
SG: stellate ganglia
SMRT: single molecule, real‐time
T: thoracic
TH: tyrosine hydroxylase
VLF: very low frequency
WT: wild‐type
αBTX: α‐bungarotoxin

## Introduction

The fate of an mRNA transcript is determined by stepwise changes in messenger ribonucleoprotein complexes, achieved through the remodeling actions of RNA‐dependent DEAD‐box ATPases (DDX in eukaryotic cells, Dbp in *Saccharomyces cerevisiae*). Providing an additional layer of regulation, some Dbps/DDXs require specific cofactors to modulate their ATPase activities. Among the known cofactors, the conserved multidomain protein GLE1 is a uniquely versatile DDX/Dbp modulator [1] playing a direct role in poly(A) + RNA nucleocytoplasmic shuttling [2], protein translation, and formation of cytosolic stress granules [3].

GLE1 dysfunction has been associated with several devastating diseases including ALS [4], rare diseases enriched in isolated populations such as lethal congenital contracture syndrome 1 [5] (LCCS1, OMIM #253310, autosomal recessive), and congenital arthrogryposis with anterior horn cell disease (CAAHD, OMIM #611890, autosomal recessive‐homozygous or compound heterozygous mutation), as well as other developmental disorders [6, 7, 8]. GLE1 malfunctions have so far been studied *in vivo* in zebrafish (*Danio rerio*), where its deficiency demonstrated a complex phenotype including immobility, edema, underdeveloped motor neurons, apoptosis in the central nervous system [9], and defective myelination of Schwann cells [10]. The changes partially mimic lethal congenital contracture syndrome 1 (LCCS1), which is an autosomal recessive disease causally linked to the GLE1 dysfunction [11]. LCCS1 is typically observed in early pregnancy due to fetal akinesia and it invariably leads to prenatal death of the fetus before the 32nd gestational week [5, 12]. The cause of death is obscure, but the fetuses show hydrops, pulmonary and skeletal muscle hypoplasia, micrognathia, and arthrogryposis, defects in anterior horn motor neurons, and severe atrophy of the ventral spinal cord. The most common variant associated with LCCS1 is *GLE1*_FinMajor_ (c.432‐10A>G), which is significantly enriched in ethnic Finns. The aberration results in alternative splicing of the *GLE1* transcript inducing an intronic retention of nine nucleotides, and thus coding extra three amino acids (PFQ) in the mutant GLE1 protein.

The *GLE1*_FinMajor_ mutation has been intensely studied *in vitro* in yeast and HeLa cells where its function was associated with disturbed mRNA nucleocytoplasmic shuttling and poly(A) + RNA accumulation in the cell nucleus, as well as malformations and disorganization of GLE1 associated complexes [13]. The *in vivo* consequences of GLE1 dysfunctions in mammals and particularly in LCCS1 and CAAHD pathologies are largely missing. This hampers our understanding of why certain cell populations are more sensitive to RNA dysregulation than others. To elucidate this, we employed mice as a model to genetically disturb GLE1 functions with CRISPR/Cas9 genome engineering. Phenotypic analyses of the two novel mouse models generated, *Gle1* knockout and *Gle1* knock‐in of the *GLE1*_FinMajor_ mutation, demonstrate the requirement of GLE1 function for gastrulation and proper development of the peripheral nervous system. These findings provide novel aspects on the function of GLE1 in development, health, and disease.

## Results

### Loss of GLE1 function compromises lineage specification in early mouse development

**Fig. 1:**
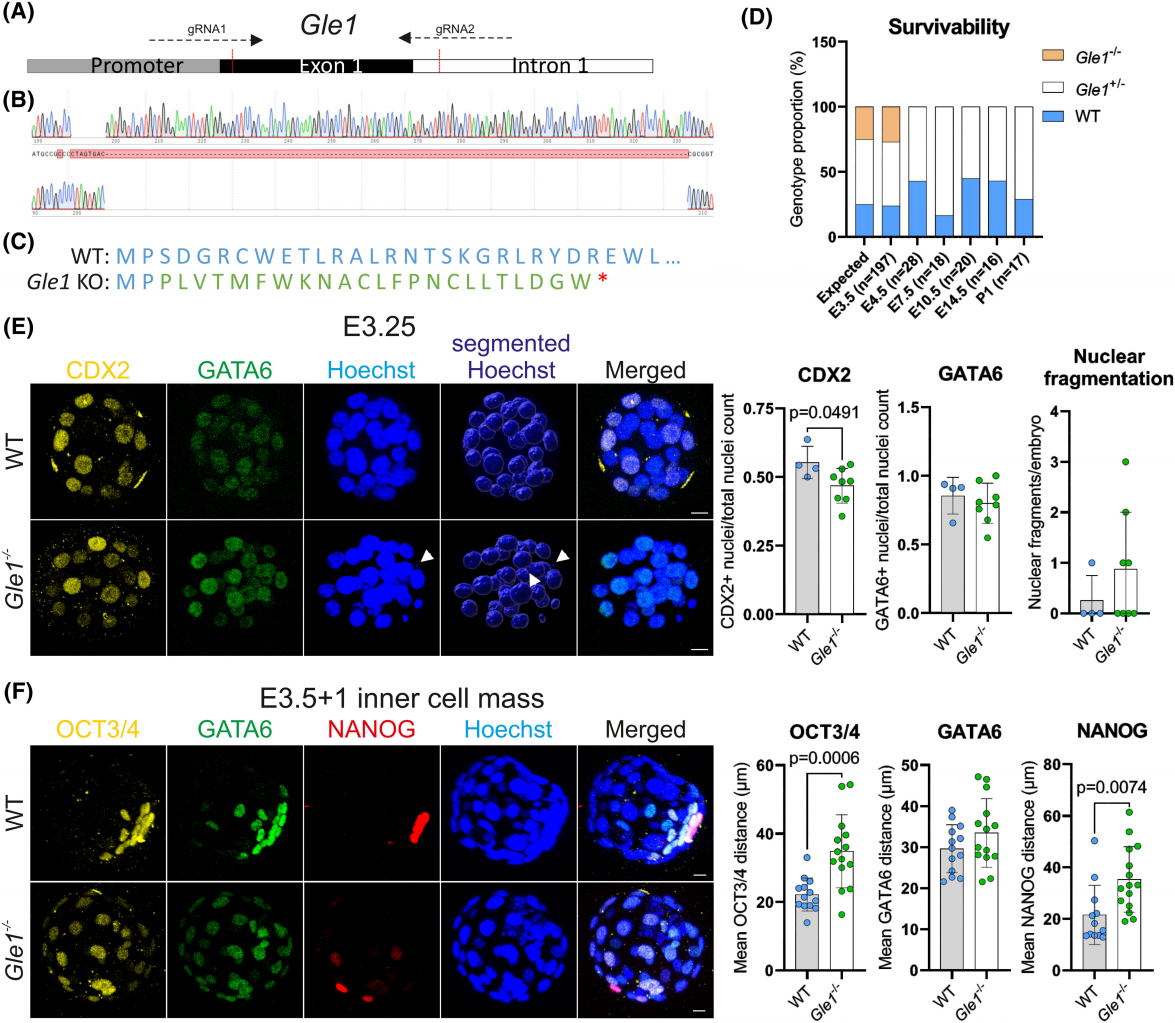
Generation of the *Gle1* knockout mice and early embryo phenotype characterization. (A) Schematics of the strategy for CRISPR/Cas9‐mediated genome engineering to target exon 1 of the *Gle1* gene by two gRNAs, aiming to induce reading frame shift and early stop codon for deletion of exon 1, potentially resulting in *Gle1* inactivation (knockout, KO). (B) Sanger sequencing results of the targeted genomic site showing the wild‐type (top) and heterozygote (wild‐type WT/KO, bottom) sequences in F2 generation. The electropherograms demonstrate a formation of around 140 base pair indel in the heterozygote DNA sequence. (C) Native amino acid sequence of the GLE1 protein (blue) in the wild‐type (WT) mice (top) and predicted amino acid sequence of the GLE1 protein post CRISPR/Cas9 mediated deletion of exon 1 (KO, bottom), predicted by Benchling, showing reading frame shift (green) and stop codon formation (red star). (D) Genotype frequencies of embryos produced from *Gle1* ^+/−^ heterozygote to heterozygote mating. E, embryonic day; Exp., expected Mendelian outcome; P, postnatal day. Samples analyzed per each stage: E3.5 *n* = 197, E4.5 = 28, E7.5 = 18, E10.5 = 20, E14.5 = 16, P1 = 17. (E) Maximum projections of early embryos cultured from 2‐cell stage for 2 days until morulae (E3.25), illustrating the presence of immunostained markers for trophectoderm (CDX2, yellow) and primitive endoderm (GATA6, green) in both WT and Gle1^−/−^ homozygotes. The quantification involves determining the number of CDX2‐positive and GATA6‐positive nuclei relative to the total number of nuclei, which are visualized by Hoechst staining (blue) as well as the number of nuclear fragments (white arrowheads) relative to the total number of nuclei. Mean ± SD are plotted, as well as individual data points generated from each embryo (WT: *n* = 4, Gle1^−/−^: *n* = 8 embryos). Scale bar 10 μm, two‐tailed Student's *t*‐test. (F) Maximum projections of blastocysts flushed from superovulated females at E3.5 and cultured for one more day (E3.5 + 1) and analysis of the immunostained inner cell mass. The quantification involves determining the distance between the nuclei expressing the inner cell mass markers: OCT3/4 (yellow), GATA6 (green, endoderm), NANOG (red, pluripotent epiblast); demonstrating an abnormal distribution of these cells all around the Gle1^−/−^ homozygote blastocyst. Hoechst staining (blue) visualizes all cell nuclei. Mean ± SD are plotted, as well as individual data points generated from each embryo (WT: *n* = 13, Gle1^−/−^: *n* = 14). Scale bar 10 μm, two‐tailed Student's *t*‐test.

To start elucidating GLE1 physiological functions *in vivo*, we first inactivated the *Gle1* function by utilizing the CRISPR/Cas9 system in mouse zygotes. The first exon was targeted by two gRNAs (Fig. 1A), leading to CAS9‐mediated cut, introduction of an approximately 140‐bp deletion (Fig. 1B, Fig. S1A) and resulting in a frameshift mutation corresponding to the premature stop codon (Fig. 1C). Heterozygote *Gle1* mice (*Gle1* ^*+/−*^) were born with expected Mendelian ratios, but no homozygote *Gle1* KO mice (*Gle1* ^−/−^) were detected after birth (Fig. 1D). Systematic stage‐by‐stage analysis of embryos collected from heterozygote mating revealed the presence of *Gle1* ^−/−^ embryos only at embryonic day 3.5 (E3.5) or earlier (Fig. 1D). Thus, inactivation of GLE1 function results in much earlier embryonic lethality than that reported for human LCCS1 fetuses [5, 12].

We next collected embryos from 2‐cell to blastocyst stage and either analyzed them directly or cultured *in vitro* to characterize their developmental and differentiation potential. Analysis of E3.25 morulae showed that overall specification to embryo proper, trophectoderm, and primitive endoderm lineages occurred similarly in *Gle1* ^−/−^ as in wild‐type (WT) control embryos (Fig. 1E). However, a slight decrease in CDX2‐positive cells was detected in *Gle1* ^−/−^ embryos, which also demonstrated an increase in OCT3/4‐positive cells and nuclear fragmentation (Fig. 1E, Fig. S1B), suggesting possible morphological or functional defects. At late blastocyst stage, an abnormal distribution of cell lineages was observed, as demonstrated by an increased mean distance between OCT3/4 and NANOG‐positive nuclei in the *Gle1* ^−/−^ blastocyst (Fig. 1F). This indicates defects in morphological changes that segregate the inner cell mass from the other lineages of the developing embryo.

Next, 5‐ethynyl‐2′‐deoxyuridine (EdU) incorporation assay was employed to assay the proliferative capacity of *Gle1* ^−/−^ embryos. This revealed a statistically significant decrease in the proportion of EdU‐positive nuclei at E3.5 (Fig. S2A). To determine whether *Gle1* ^−/−^ blastocysts exhibit reduced proliferation due to increased cell death, apoptosis was measured by TUNEL (terminal deoxynucleotidyl transferase dUTP nick end labeling) and cleaved Caspase 3 (c‐CASP3) staining. Indeed, TUNEL‐positive nuclei were significantly augmented in the *Gle1* ^−/−^ blastocysts at E3.5, and an increase in c‐CASP3 abundance was detected at late blastocyst stage (E3.5 + 1, Fig. S2B,C). Such changes in viability at the late blastocyst stage likely explain the failure to thrive in the absence of GLE1 functions.

### *Gle1* ^−/−^ blastocyst shows transcriptional changes in cell adhesion, ion channels, and transmembrane transport machinery

**Fig. 2:**
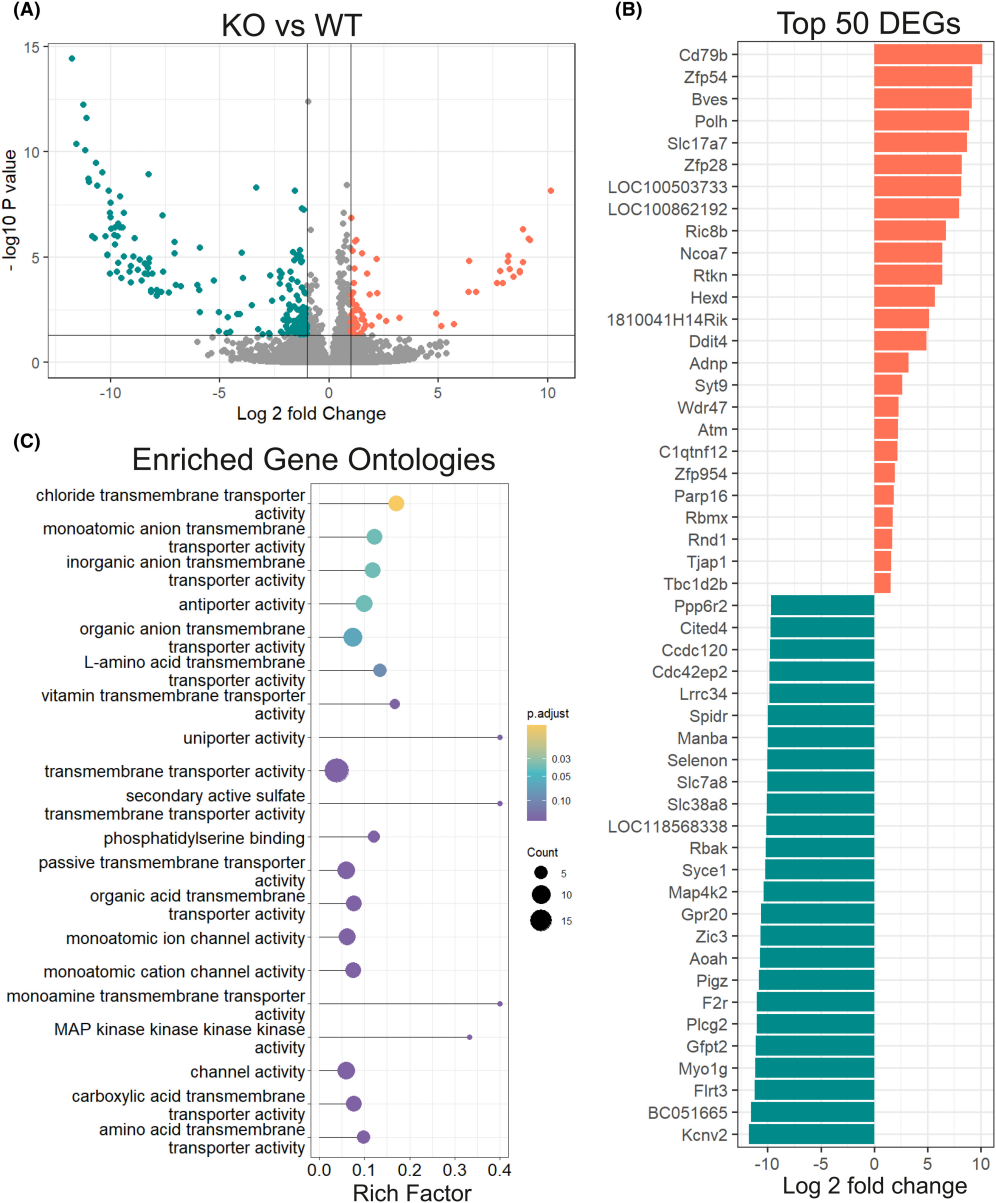
Transcriptional profiling of E3.5 *Gle1* knockout blastocysts. (A) Volcano plot showing the 242 differentially expressed genes (DEGs) in bulk RNA‐seq analysis of E3.5 blastocysts (RNA from three blastocysts per biological replicate, three replicates per genotype). Significantly differentially expressed genes in *Gle1* knockout (KO) embryos (log_2_ fold change |1|; adjusted *P*‐value < 0.05) are indicated in green (downregulated) and orange (upregulated). This identified total of 65 upregulated and 177 downregulated genes. WT, wild‐type. (B) Identities of top 50 deregulated genes in *Gle1* KO embryos. Orange: top 25 upregulated, green: top 25 downregulated transcripts and their log_2_ fold changes. (C) Dot plot of gene ontology enrichment analysis visualizing top 20 molecular functions affected by *Gle1* inactivation. Genes with adjusted *P*‐value < 0.05 and log_2_ fold change > 1 in either direction were used for analysis.

In search of transcriptional changes that could suggest mechanisms behind defective development in *Gle1* ^−/−^ embryos, a bulk RNA‐sequencing (RNA‐seq) of the E3.5 blastocysts was performed. Initial quality checks demonstrated good segregation and similar distribution of WT and *Gle1* ^−/−^ transcripts and identified 242 differentially expressed genes (65 up‐ and 177 downregulated) with the cutoff of log_2_ fold change |1| and adjusted *P*‐value < 0.05 (Fig. 2A, Fig. S3A,B, Data S1). The most significantly downregulated transcripts in *Gle1* ^−/−^ blastocysts (*Kcnv2*, *Flrt3*, *Gfpt2*, *Myo1g*, unannotated BC051665, *Plcg2* and *Zic3*) failed to be detected in all samples by RNA‐seq while showing robust counts in control embryos (Fig. 2B, Data S1). This suggests that GLE1 function in blastocysts is required for their expression. Potassium voltage‐gated channel *Kcnv2* (Data S1) encodes the ether‐à‐go‐go‐related channel (hERG1) often dysregulated in schizophrenia, cardiac arrhythmia, and tumor proliferation, as well as in normal cell motility involving VIMENTIN (VIM) [14, 15, 16, 17]. *Vimentin* (*Vim*) itself and several gene products encoding adhesion and extracellular matrix interacting molecules (*Dag1*, *Fndc10*, *Igsf8*, *Igsf9b*, *Cldn23*) and their regulators (*Plcg2*, *Rab15*, *Pigz*, *Ppp6r2*) were among the most significantly downregulated transcripts in *Gle1* ^−/−^ embryos (Data S1). Of the most downregulated genes, *Dystroglycan 1* (*Dag1*) inactivation results in early embryonic lethality (E6.5) since it is required for laminin‐dependent polarization of the epiblast [18, 19]. Interestingly, myosin 1G (*Myo1g*) is required for proper cardiac differentiation in *Drosophila* [20] while loss of zinc finger protein of the cerebellum 3 (*Zic3*) in mice causes neural tube and heart defects [21].

Among the most significantly upregulated transcripts in *Gle1* ^−/−^ embryos were *Cd79b*, required for B cell development, and *Polh*, which encodes DNA polymerase eta (Data S1) with essential survival functions in replicating damaged DNA [22, 23]. Gene ontology (GO) analysis revealed the most significant changes in transcripts participating in ion channel and transmembrane transport activities (Fig. 2C). The transcriptional changes in genes associated with ion channels and transmembrane transport activities suggest that the phenotype in early *Gle1* ^−/−^ blastocyst development may be linked to impaired trans‐trophectodermal ion gradient formation. Such gradients, together with junctional tightening, are crucial for morphological changes that physically separate cell lineages during development [24].

### Knock‐in of the LCCS1 *Gle1* ^PFQ/PFQ^ variant to mouse genome causes premature death

**Fig. 3:**
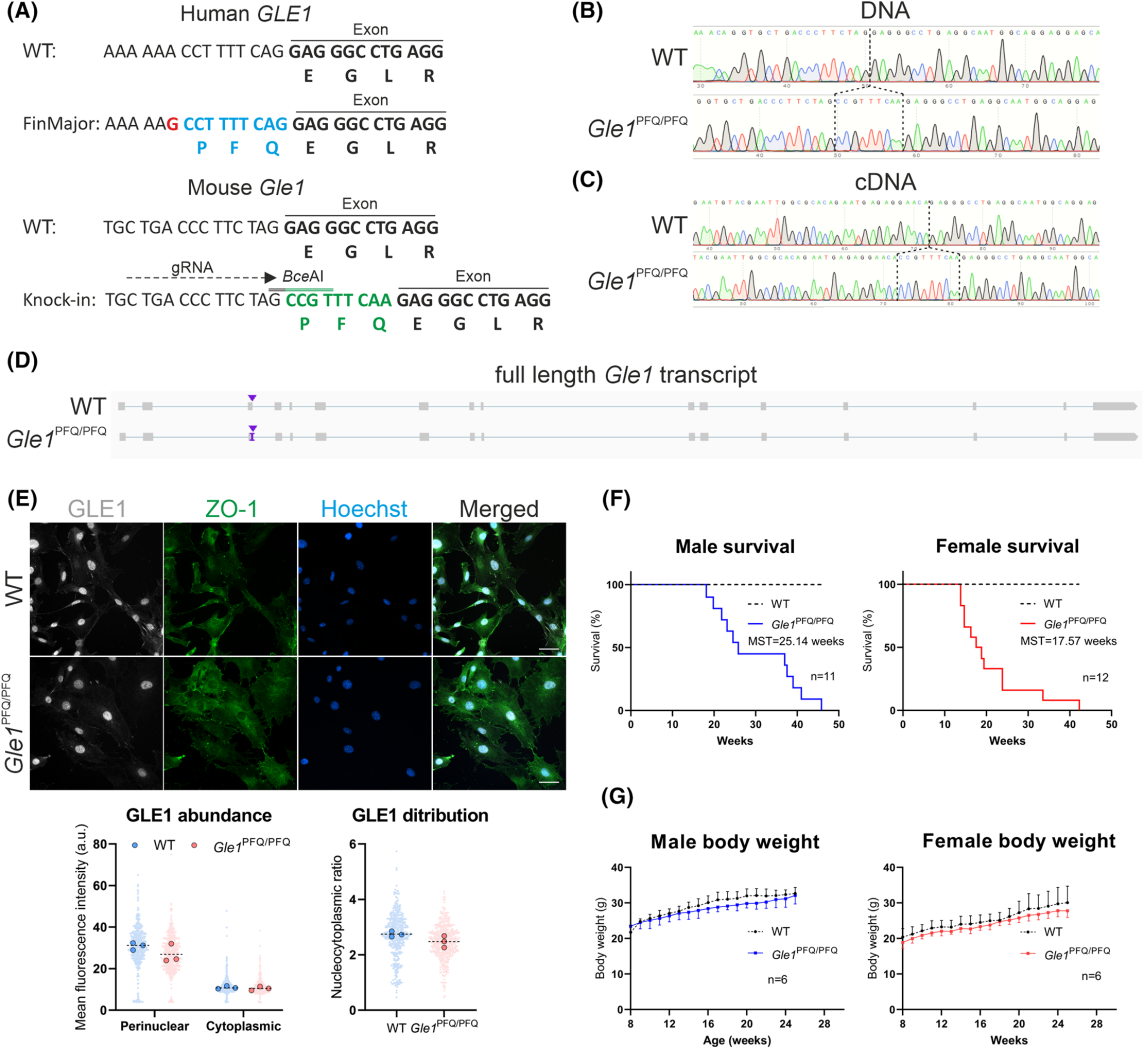
Generation of the *Gle1* ^PFQ/PFQ^ KI mice. (A) Schematics of the target locus comparing the human and mouse sequences of the *GLE1/Gle1* gene, demonstrating similarity in the exon, but major difference in the target intron. This variability mitigates the possibility of introducing the human A‐to‐G substitution (red), which would induce the alternative splicing and nine nucleotide intron retention (blue), into mice. Nine nucleotides were directly knocked‐in (green) at the mouse intron/exon boundary, mimicking the human alternative splicing outcome, with two silent mutations including a digestion site for genotyping purpose (green line above the inserted base pairs shown in green). Accession number of human *GLE1* is NM_001003722.2 and mouse NM_028923.3. Sequences were aligned with Snap Gene. (B) Sequence results of the target site from the DNA of wild‐type (WT, top) and KI/KI homozygote (bottom) mice, isolated from ear samples of the F2 generation mice. The Sanger sequencing electropherograms demonstrate a successful knock‐in of the desired nine nucleotides (CCGTTTCAA) at the intron/exon boundary of *Gle1*. Sequence shown in the figure: ATG CCG CCC TAG TGA C….CGC GGT. (C) Sequence results of the target site from the reverse transcribed cDNA of wild‐type (top) and KI/KI homozygote (bottom) mice. RNA was isolated from the heart of adult (10 weeks old) mice at the F2 generation. The Sanger sequencing electropherograms demonstrate a successful transcription of the inserted nine nucleotides in the KI homozygotes, mimicking the outcome of the alternative splicing in the GLE1_FinMajor_ mutation. (D) Analysis of the *Gle1* transcript full length and sequence in the WT and *Gle1* KI mice. Visualization of long‐range RNA‐seq reads in IGV, produced by the PacBio SMRT sequencing and using the RNA isolated from the postnatal day zero (P0) hearts. Purple arrowhead indicates the nine‐nucleotide insertion into the mouse *Gle1* transcript (NM_028923.3). (E) Immunofluorescent analysis of GLE1 (gray) protein abundance and distribution in mouse embryonic fibroblasts, counterstained by ZO‐1 (green). Individual data points generated from each cell are plotted (150 cells per embryo, three embryos per genotype), as well as median values from each embryo (large dots). Scale bar: 50 μm. (F) Kaplan–Meier survival curve of the WT and Gle1 KI males and females, showing the premature lethality of KI homozygotes in mid‐adulthood and lower median survival time (MST) in KI females than KI males (WT males: 11, KI males: 11, WT females: 12, KI females 12). (G) Analysis of the body weight development in the adult WT and KI homozygote males and females (WT males: 6, KI males: 6, WT females: 6, KI females: 6). Mean ± SD are plotted.

The early lethality of *Gle1* ^−/−^ mice indicates that the LCCS1‐associated *GLE1*_FinMajor_ variant, which is an intronic mutation, does not fully inactivate GLE1 function, but otherwise modifies its functions. Next, we thus wanted to model LCCS1 in a mammalian model and introduced the human disease‐causing *GLE1* variant into the mouse genome. Although the *GLE1*/*Gle1* exons are highly similar between humans and mice, the introns are not. Therefore, it is likely that introducing the intronic A‐to‐G change in mice would not induce similar alternative splicing as in human patients (Fig. 3A). To bypass the species differences in intronic sequences, we straightforwardly knocked‐in (KI) nine nucleotides (coding the PFQ amino acids) to the intron/exon boundary of the mouse *Gle1* gene (Fig. 3A,B) to generate the *Gle1* ^*em1Skuu*^ mouse line.

The *Gle1* ^*em1Skuu/em1Skuu*^ mice, hereafter referred to as *Gle1* ^PFQ/PFQ^ and *Gle1* KI mice, were born at Mendelian ratios, and successful transcription of the inserted nine nucleotides was confirmed in adult *Gle1* ^PFQ/PFQ^ KI homozygotes (Fig. 3C, Fig. S4A,B). The result demonstrated a similar transcriptional outcome as detected in cDNA of human *GLE1*_FinMajor_ fetuses [5]. To confirm that the modification did not induce unintentional splicing events of the *Gle1* transcript, we performed full‐length transcript sequencing using the PacBio SMRT (Single Molecule, Real‐Time). This verified the length and sequence for mouse WT and KI *Gle1* transcripts (Fig. 3D) and demonstrated the lack of undesired splicing. No changes in the expression of *Gle1* transcript or protein levels were detected between WT and *Gle1* ^PFQ/PFQ^ adult mouse hearts (Fig. S4C,D), nor did the GLE1 abundance or distribution change in mouse embryonic fibroblasts (MEFs) (Fig. 3E).

Structurally, the inserted PFQ amino acids are in the GLE1 N‐terminal domain and associate with protein oligomerization in a phosphorylation‐dependent manner, thus being important for many of GLE1‐associated functions [1, 13]. Comparison of bioinformatically predicted domains from human and mouse amino acid sequences demonstrated an equal localization of the PFQ amino acid insertion at the proximal boundary of the predicted coiled coil domain, as well as proximity to one of the aggregation hotspots (Fig. S4E,F). These predictions suggest that our *Gle1* ^PFQ/PFQ^ mouse model is biologically valid to study the human LCCS1 disease. However, while the LCCS1 patients die during fetal development, the *Gle1* ^PFQ/PFQ^ mice survive until midadulthood before dying suddenly overnight, without any obvious changes in growth, welfare, or body weight (Fig. 3F,G). This implies a difference in how GLE1 PFQ insertion affects protein functions in mouse and man, suggesting either species differences or alterations deriving from editing strategy. Interestingly, the median survival time (MST) in the *Gle1* ^PFQ/PFQ^ females (MST = 17.57 weeks) was shorter than in *Gle1* ^PFQ/PFQ^ males (MST = 25.14 weeks), implying a sex bias in phenotypic severity, which is not explored in LCCS1 fetuses.

### *Gle1* ^PFQ/PFQ^ KI mouse embryonic fibroblasts demonstrate signals of premature senescence

**Fig. 4:**
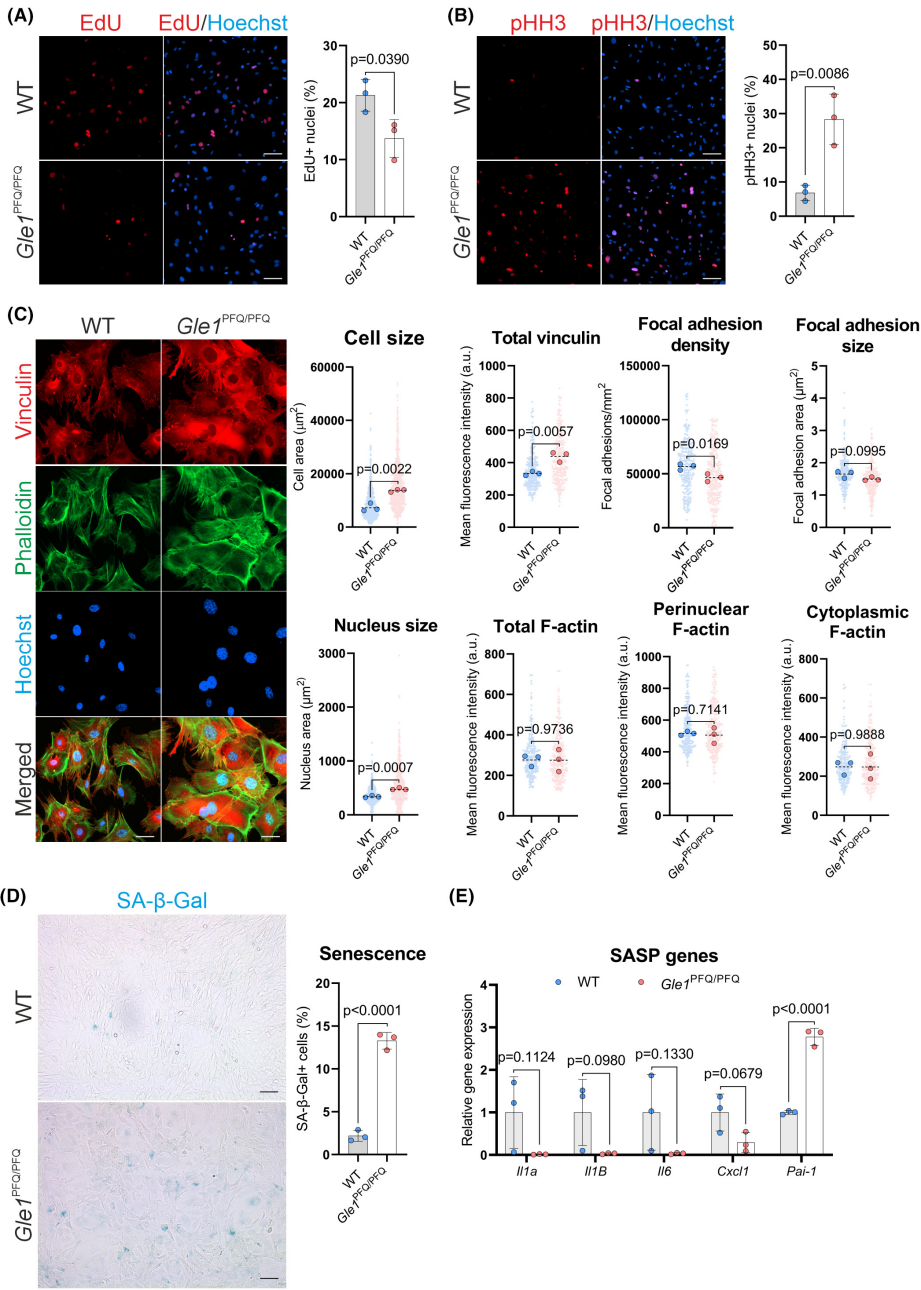
Disturbed cell cycle and senescence progression *Gle1* ^PFQ/PFQ^ in mouse embryonic fibroblasts. (A) Cell proliferation assay with quantification of 5‐ethynyl‐2′‐deoxyuridine (EdU) uptake (red, 10 μm EdU, 6 h). Mean ± SD are plotted, as well as individual data points generated from each embryo (three embryos per genotype, over 500 cells analyzed per embryo). Scale bar 100 μm, two‐tailed Student's *t*‐test. (B) Immunofluorescent staining and quantification of phospho‐Histone H3 (pHH3) + (red) mitotic cells. Mean ± SD are plotted, as well as individual data points generated from each embryo (three embryos per genotype, over 500 cells analyzed per embryo). Scale bar 100 μm, two‐tailed Student's *t*‐test. (C) Vinculin and phalloidin stained MEFs were used to analyze the cell size, focal adhesions, and F‐actin distribution. Individual data points generated from each cell are plotted (100 cells per embryo, three embryos per genotype), as well as median values from each embryo (large dots). Scale bar 50 μm, two‐tailed Student's *t*‐test. Hoechst staining (blue) visualizes all cell nuclei in A–C. (D) Senescence‐associated β‐galactosidase (SA‐β‐Gal) staining (blue) of mouse embryonic fibroblasts (MEFs) as a marker of cellular senescence. Mean ± SD are plotted, as well as individual data points generated from each embryo (three embryos per genotype, over 400 cells analyzed per embryo). Scale bar 200 μm, two‐tailed Student's *t*‐test. (E) RT‐qPCR quantification of senescence‐associated secretory profile (SASP) genes in MEFs at passage 4. Mean ± SD are plotted, as well as individual data points generated from each embryo (three embryos per genotype). Two‐tailed Student's *t*‐test. WT, wild‐type.

To dissect the effects of the KI modification, we analyzed basic cellular properties in MEFs. Flow cytometry analysis of MEFs showed an increase in the G2/M population of *Gle1* ^PFQ/PFQ^ cells (Fig. S5A), while the EdU incorporation assay demonstrated a lower rate of proliferation in *Gle1* ^PFQ/PFQ^ MEFs than in WT cells (Fig. 4A). Interestingly, a striking increase in the number of phospho‐histone H3 (Ser10)‐positive (pHH3) mitotic nuclei was detected in *Gle1* ^PFQ/PFQ^ MEFs (Fig. 4B). This suggests that the *GLE1*_FinMajor_ variant disrupts cell cycle progression, as *Gle1* ^PFQ/PFQ^ MEFs are dominantly mitotic, suggesting that they are arrested in M phase. A similar cell cycle progression defect was previously described after the knockdown of *GLE1* in HeLa cells [25].

The cell shape and size of *Gle1* ^PFQ/PFQ^ MEFs differed by eye from WT cells and prompted us to study this more in detail. Analysis of cell size revealed that *Gle1* ^PFQ/PFQ^ cells are significantly larger than wild‐type cells, as shown by overall size and the abundance of vinculin‐positive focal adhesions, which in *Gle1* ^PFQ/PFQ^ cells showed similar size to wild‐type cells but a significant decrease in the density (Fig. 4C). No measurable alterations in cytoskeletal organization, as assayed by F‐actin distribution and abundancy, were detected (Fig. 4C).

The significant increase of *Gle1* ^PFQ/PFQ^ cells in mitosis, together with the observed cytomorphological changes in cell size, resembles hallmarks of cellular senescence [26, 27, 28]. Such growth arrest is often triggered by persistent response to DNA damage or stress signaling [29, 30, 31, 32], and senescent cells usually exhibit increased lysosomal β‐galactosidase activity [33]. Under certain conditions, senescent cells also secrete interleukins, inflammatory cytokines, and growth factors, which are recognized as the senescent‐associated secretory profile (SASP) [34, 35]. To further analyze possible premature senescence in *Gle1* ^PFQ/PFQ^ MEFs, we utilized senescence‐associated β‐galactosidase (SA‐β‐gal) and DNA damage assays. Strikingly, while the wild‐type cells barely showed any β‐gal‐positive cells, their abundance in *Gle1* ^PFQ/PFQ^ MEFs was statistically significantly increased (Fig. 4D). Analysis of DNA damage by staining with phospho‐histone H2A.X (Ser 139) (γH2A.X) revealed an increase in *Gle1* ^PFQ/PFQ^ cells, which additionally showed decreased expression of cell cycle regulators *Cdkn2a* (p16, p19) and *Cdkn1a* (p21) (Fig. S5B,C). Out of the tested SASP‐related genes, the expression of *Il1α*, *Il1β*, *Il6*, and *Cxcl1* was downregulated, while *Pai‐1* presented a significantly increased expression in the *Gle1* ^PFQ/PFQ^ MEFs (Fig. 4E). Thus, our characterization of *Gle1* ^PFQ/PFQ^ cellular features indicates that the genetic LCCS1 variant *Gle1* ^PFQ/PFQ^ induces cellular senescence in MEFs.

It has been described that senescent cells may suffer from insufficient protein degradation, for example via the ubiquitin‐proteasome system, resulting in accumulation of damaged proteins and a disturbed proteostasis [36, 37]. Western blot analysis of ubiquitinated proteins and measurement of proteasome 20S activity (Fig. S5D,E) revealed no changes in *Gle1* ^PFQ/PFQ^ MEFs. Therefore, the observed cellular phenotypes are less likely to stem from impaired proteostasis but may instead reflect stress response dysfunction.

### *Gle1* ^PFQ/PFQ^ variant in mouse allows intact mRNA shuttling but disrupts cellular stress responses

**Fig. 5:**
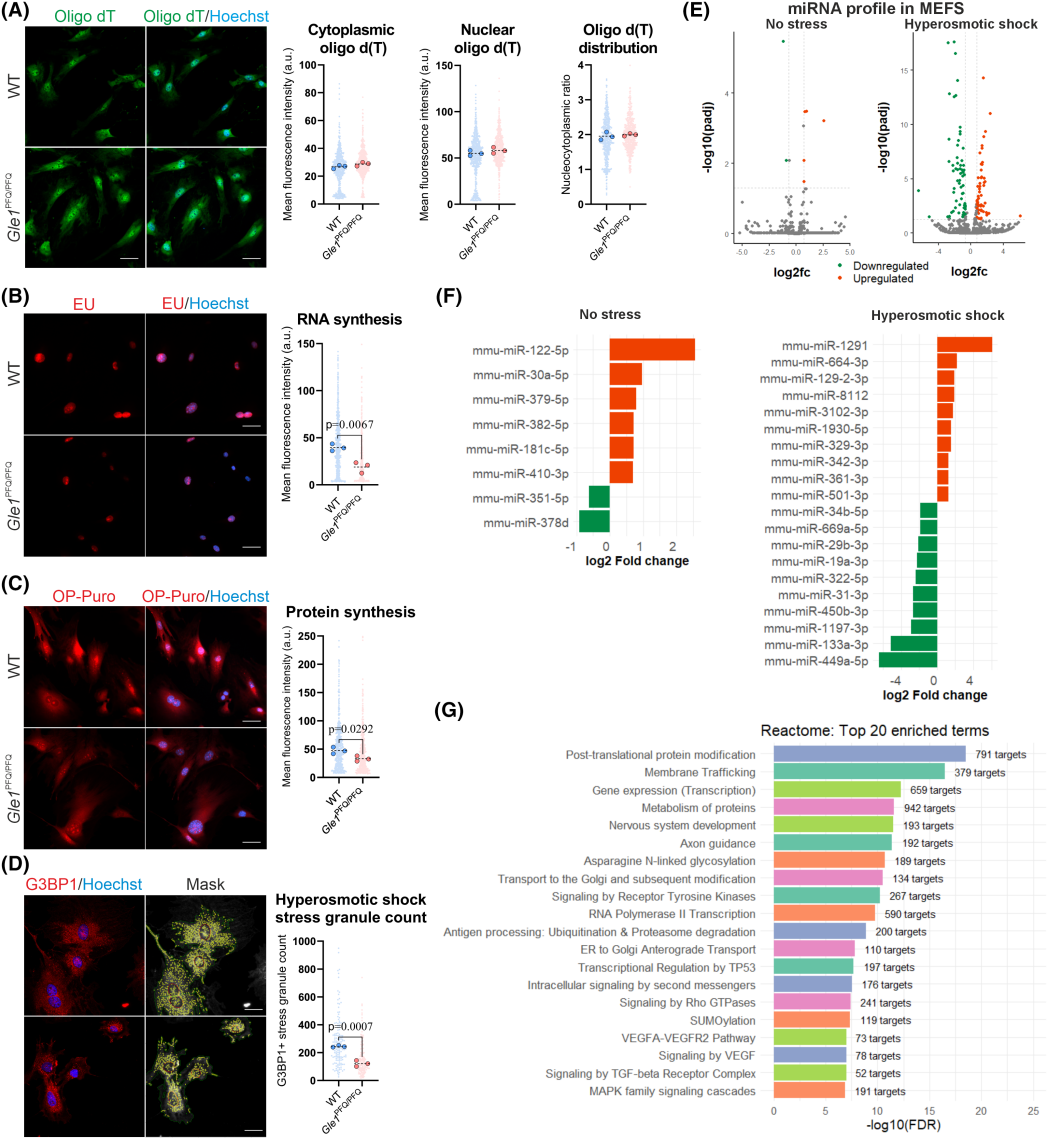
GLE1 functional analysis using E13.5 mouse embryonic fibroblasts. (A) Visualization and quantification of poly(A) + RNA through fluorescent *in situ* hybridization of 5′‐Cy3‐oligo d(T)30 probe in mouse embryonic fibroblasts (MEFs). Individual data points generated from each cell are plotted (200 cells per embryo, three embryos per genotype), as well as median values from each embryo (large dots). Scale bar 50 μm. (B) Quantification of RNA synthesis via ethynyl uridine (EU) metabolic incorporation (1 mm EU, 1 h pulse). Individual data points generated from each cell are plotted (150 cells per embryo, three embryos per genotype), as well as median values from each embryo (large dots). Scale bar 50 μm, two‐tailed Student's *t*‐test. (C) Quantification of protein synthesis via O‐propargyl‐puromycin (OP‐puro) metabolic incorporation (20 μm OP‐Puro, 30 min pulse). Individual data points generated from each cell are plotted (200 cells per embryo, three embryos per genotype), as well as median values from each embryo (large dots). Scale bar 50 μm, two‐tailed Student's *t*‐test. (D) Maximum projection of MEFs stained with G3BP1 (red) after stress challenge with hyperosmotic shock (400 mm sorbitol, 1 h) followed by stress granule quantification as G3BP stress granule assembly factor 1 (G3BP1)‐positive speckle‐type objects (yellow) in the cytoplasm (green) and around the nucleus (red). Hoechst staining (blue) visualizes all cell nuclei. Individual data points show total stress granule signal generated in each cell (50 cells per embryo, three embryos per genotype), as well as median values from each embryo (large dots). Scale bar 30 μm, two‐tailed Student's *t*‐test. (E) Volcano plots showing differentially expressed miRNAs from RNA‐seq analysis of *Gle1* ^PFQ/PFQ^ MEFs (three embryos per genotype) under no stress or after hyperosmotic shock (400 mm sorbitol, 1 h). Significantly differentially expressed miRNAs (log_2_ fold change > 0.7; adjusted *P*‐value < 0.05) are indicated in green and orange. (F) Plots of differentially expressed miRNAs in *Gle1* ^PFQ/PFQ^ MEFs under normal condition (left), and top 20 dysregulated microRNAs (miRNAs) in MEFs under hyperosmotic shock (right; top 10 upregulated: orange, and top 10 downregulated: green) and their log_2_ fold change. (G) Significant enrichment of specific biological pathways associated with differentially expressed miRNAs in *Gle1* ^PFQ/PFQ^ MEFs under hyperosmotic shock, identified by miRPath‐v4. WT, wild‐type.

Previous *in vitro* experiments have established a basis for *GLE1*_FinMajor_ driven pathology, which lies in disturbed nucleocytoplasmic shuttling of poly(A) + RNA leading to its accumulation in the cell nucleus [13]. Interested if this is the potential cellular mechanism disrupting GLE1 functions in mouse *Gle1* ^PFQ/PFQ^ cells as well, we analyzed the mRNA distribution by oligo d(T) fluorescent *in situ* hybridization (FISH) followed by quantification of poly(A) + RNA subcellular localization. These experiments found no differences in the oligo d(T) abundance or nucleocytoplasmic distribution between genotypes (Fig. 5A). Motivated by the known involvement of GLE1 in translation [3] we further investigated if, despite normal poly(A) + RNA distribution, the *Gle1* ^PFQ/PFQ^ variant would impact RNA and/or protein synthesis. Metabolic labeling of RNA with ethynyl uridine (EU) indeed identified a decrease in RNA synthesis (Fig. 5B). Similarly, O‐propargyl‐puromycin (OP‐puro) incorporation demonstrated diminished protein synthesis (Fig. 5C). Notably, these experiments cannot distinguish if the identified changes in RNA synthesis and protein translation are primarily induced by modified GLE1 functions or secondarily by changes in general cellular activities.

To further assess possible pathological mechanisms beyond mRNA nucleocytoplasmic transport, we next addressed the ability of *Gle1* ^PFQ/PFQ^ cells to handle and react to stress, which typically induces integrated stress response (ISR). This pathway is activated by phosphorylation of the alpha subunit of eukaryotic translation initiation factor 2 (eIF2α, serine 51) to tackle a range of physiological changes and several different pathological conditions [38, 39, 40, 41]. Importantly, activation of ISR modulates the transcriptional and translational responses of the stressed cells to facilitate handling of the immediate effects of the threat [42]. At the subcellular level, an acute response to environmental stressors and activation of the ISR promotes stress granule formation [43, 44].

We stressed the WT and *Gle1* ^PFQ/PFQ^ MEFs with three different conditions followed by measurement of p‐eIF2α levels. Cells in all stress inducer conditions, hyperosmotic shock (sorbitol), oxidative shock (sodium arsenite), and heat shock (43 °C) had similar p‐eIF2α levels and thus ISR activation regardless of their genotype (Fig. S6A). However, quantification of stress granules by visualizing the protein G3BP stress granule assembly factor 1 (G3BP1) demonstrated a significant decrease in the total count of G3BP1‐positive areas per cell in *Gle1* ^PFQ/PFQ^ MEFs under all of the tested conditions (Fig. 5D, Fig. S6B,C). This shows that although *Gle1* ^PFQ/PFQ^ MEFs have normal activation of p‐eIF2α indicating an intact ISR pathway, they yet have altered capacity to handle stress suggesting essential functions for GLE1 in cellular stress response.

Cellular stress response involves also changes in small noncoding RNAs, such as tRNAs and miRNAs, which tailor the protein synthesis to respond to the stress in a timely and targeted manner [45, 46, 47, 48]. We next performed small noncoding RNA sequencing of control and *Gle1* ^PFQ/PFQ^ MEFs grown in normal and hyperosmotic shock conditions (Fig. S6D,E). This revealed similar abundance of tRNA pools under normal conditions, and only two differentially expressed tRNAs in stressed cells at the 60 min timepoint: upregulated AspGTC and downregulated SerGGA (Fig. S6F,G, Data S2). In contrast, major changes in miRNA profiles were detected in stressed *Gle1* ^PFQ/PFQ^ MEFs (Data S2) suggesting that epigenetic regulation plays a major role in the acute stress response of the *Gle1* ^PFQ/PFQ^ MEFs. While under normal conditions only eight miRNAs were differentially expressed (six upregulated and two downregulated), 73 miRNAs were dysregulated (32 upregulated and 31 downregulated) between WT and *Gle1* ^PFQ/PFQ^ hyperosmotically shocked cells (Fig. 5E,F, Data S2). Reactome pathway analysis identified transcription, protein metabolism, and nervous system development together with axon guidance as biological processes mostly affected by the *Gle1* ^PFQ/PFQ^ variant in stressed MEFs (Fig. 5G). These align well with the clinical manifestation of neurodevelopmental defects in LCCS1 disease characterized by severe motor neuron pathology and suggest that GLE1 dysfunction may disrupt early tissue innervation by altering post‐transcriptional regulation of key developmental genes [49, 50, 51].

### Impaired motor neuron organization in the developing ventral spinal cord of *Gle1* ^PFQ/PFQ^ mice

Following the detailed analysis of morphological and functional changes in *Gle1* KI MEFs, we focused on examining the developmental abnormalities induced by the *Gle1* ^PFQ/PFQ^ variant in mice. One of the most profound phenotypes of LCCS1 is the loss of ventral horn motor neurons, resulting in fetal akinesia [5]. Primary characterization of E11.5 brachial spinal cords revealed a comparable count of OLIG2‐positive motor neuron (MN) progenitors in WT and *Gle1* KI embryos (Fig. S7A). However, a statistically significant reduction in ISL1/2‐positive general MNs was detected in the lateral motor column of *Gle1* ^PFQ/PFQ^ embryos (Fig. S7B) verifying the mouse model phenotype similar but clearly milder than that reported in LCCS1 fetuses. Like in MEFs, oligo d(T) FISH analysis of embryonic spinal cords presented no changes in poly(A) RNA distribution (Fig. S7C), supporting our *in vitro* findings that suggest other than a global nucleocytoplasmic mRNA shuttling defect as a contributing molecular mechanism of LCCS1 pathology.

**Fig. 6:**
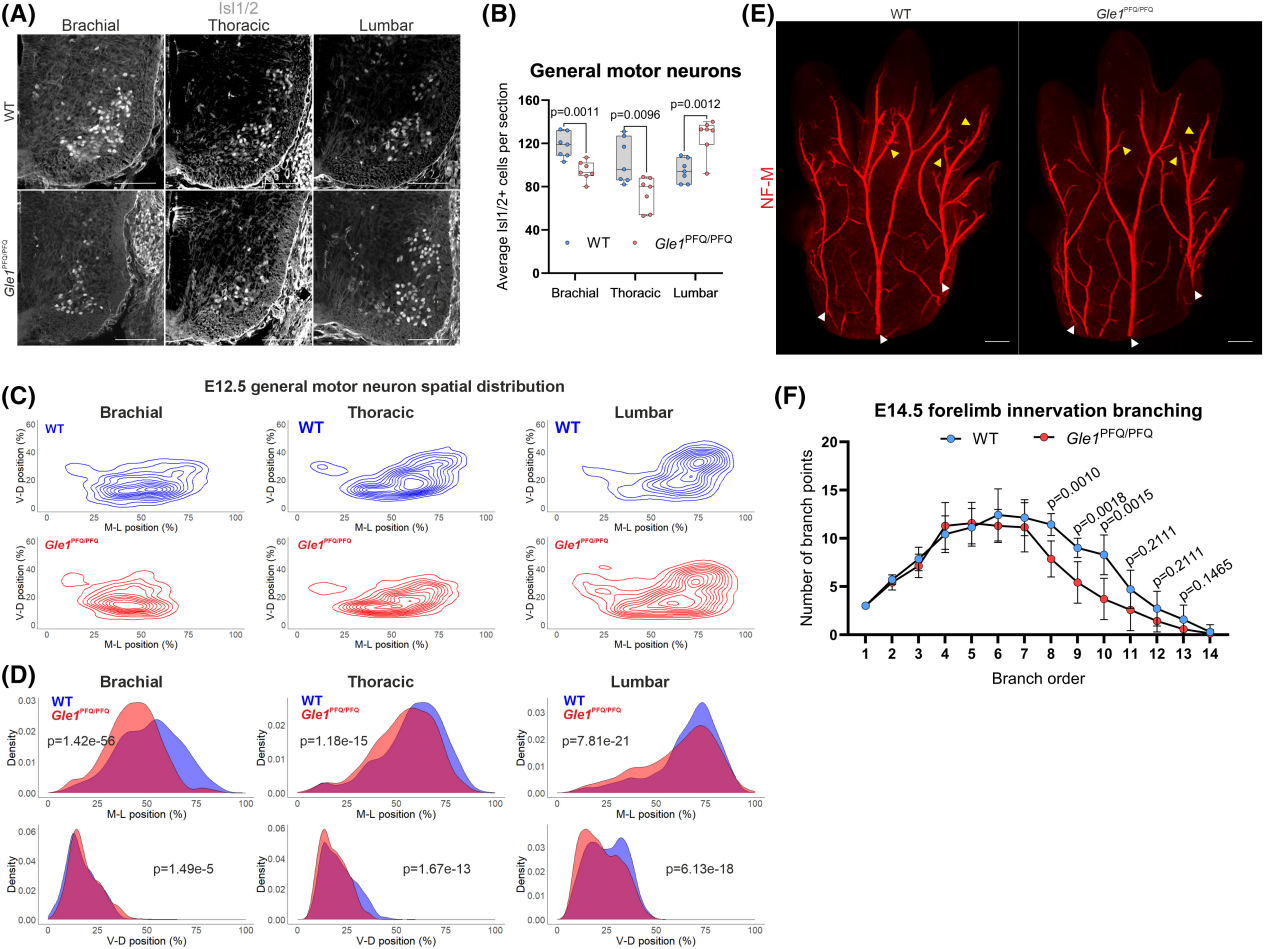
Aberrant number and distribution of spinal cord ventral general motor neurons in *Gle1* ^PFQ/PFQ^ embryos. (A) Representative images of E12.5 ventral spinal cord transverse hemi‐sections at the brachial, thoracic, and lumbar regions, immunolabeled for Islet1/2 (ISL1/2, white) as a marker of general motor neurons. Seven embryos per genotype, with multiple sections per each spinal region – brachial: three, thoracic and lumbar: four. Scale bar 100 μm. (B) Quantification of the ISL1/2 immunostained E12.5 general motor neurons in the ventral spinal cord at the brachial, thoracic, and lumbar regions. Box and whisker plots, as well as individual data points (*n* = 7) generated from each mouse (i.e., the average motor neuron count from multiple sections – brachial: 3, thoracic: 4, lumbar: 4), two‐tailed Student's *t*‐test. (C) Contour density plots demonstrating the qualitative changes in relative spatial distribution of the E12.5 ventral spinal cord ISL1/2‐positive general motor neurons in the medio‐lateral (M‐L) and ventro‐dorsal (V‐D) planes. Seven embryos per genotype, with multiple sections per each spinal region – brachial: three, thoracic and lumbar: four. (D) Density plots highlighting the quantitative changes in relative spatial distribution of the E12.5 ventral ISL1/2 immunostained general motor neurons in the medio‐lateral (M‐L) and ventro‐dorsal (V‐D) planes. Kolmogorov–Smirnov test. Figures in A and E show representative images for each genotype. Seven embryos per genotype and multiple sections per each spinal region (brachial: three, thoracic and lumbar: four) were analyzed. (E) Whole‐mount images of neurofilament‐M (NF‐M, red) immunostained E14.5 right forelimb paws imaged on the dorsal side. White arrowheads indicate the three major nerve trunks analyzed. Yellow arrows indicate higher‐order branches showing major branching differences between genotypes. Seven embryos per genotype, with multiple sections per each spinal region – brachial: three, thoracic and lumbar: four. Scale bar 100 μm. (F) Quantification of branch structures in NF‐M‐stained E14.5 forelimbs. Branch points at each order (number of branching events per trunk) were summed across the three nerve trunks per sample. Shown are mean ± SD, seven mice per genotype, two‐tailed Student's *t*‐test. WT, wild‐type.

It has been described that the spinal cord in LCCS1 fetuses is macroscopically thinned because of an early reduction of the anterior horn and a paucity of anterior horn cells [52]. To assess if the *Gle1* KI spinal cords present any morphological phenotype early in development, we first quantified the number of Sox2‐positive progenitors and SOX2/pHH3‐positive mitotic cells in the neural tubes without detecting differences between the genotypes (Fig. S8A,B). Next, more detailed scrutiny of ISL1/2‐positive general MNs was performed in the E12.5 ventral spinal cord. This identified that indeed, brachial and thoracic spinal cord segments have a lower number of MNs in *Gle1* ^PFQ/PFQ^ than WT embryos, but also revealed increased MN count in the lumbar segment (Fig. 6A,B).

The irregularities in MN numbers are likely to affect the developing neural circuitry and modify the choices for synaptic pruning to optimize the neural network during development [53], which can be further vitiated by the shift in MN distribution. Accordingly, the relative localization of MNs in all of the studied E12.5 spinal cord segments was altered in a way that the MNs in *Gle1* ^PFQ/PFQ^ embryos were positioned more medially in the medio‐lateral axis than in WT embryos (Fig. 6C). At the ventro‐dorsal axis, the *Gle1* ^PFQ/PFQ^ brachial MNs were scattered slightly more dorsally, in contrast to the KI thoracic and lumbar MNs being shifted more ventrally than the arrangement detected in WTs (Fig. 6D).

It has been previously described that *gle1* inactivation in zebrafish results not only in fewer motor neurons, but also in disorganized axon arborization [9]. Encouraged by this, we performed whole‐mount analysis of E14.5 forelimb paws to address axonal development in *Gle1* ^PFQ/PFQ^ mice. We found that while the number of NF‐M‐positive axon branch points was significantly reduced at the 8th branching order and higher, this was primarily due to diminished axonal side branching (Fig. 6E,F).

### Adult *Gle1* ^PFQ/PFQ^ spinal motor neurons show minor changes without functional consequences

**Fig. 7:**
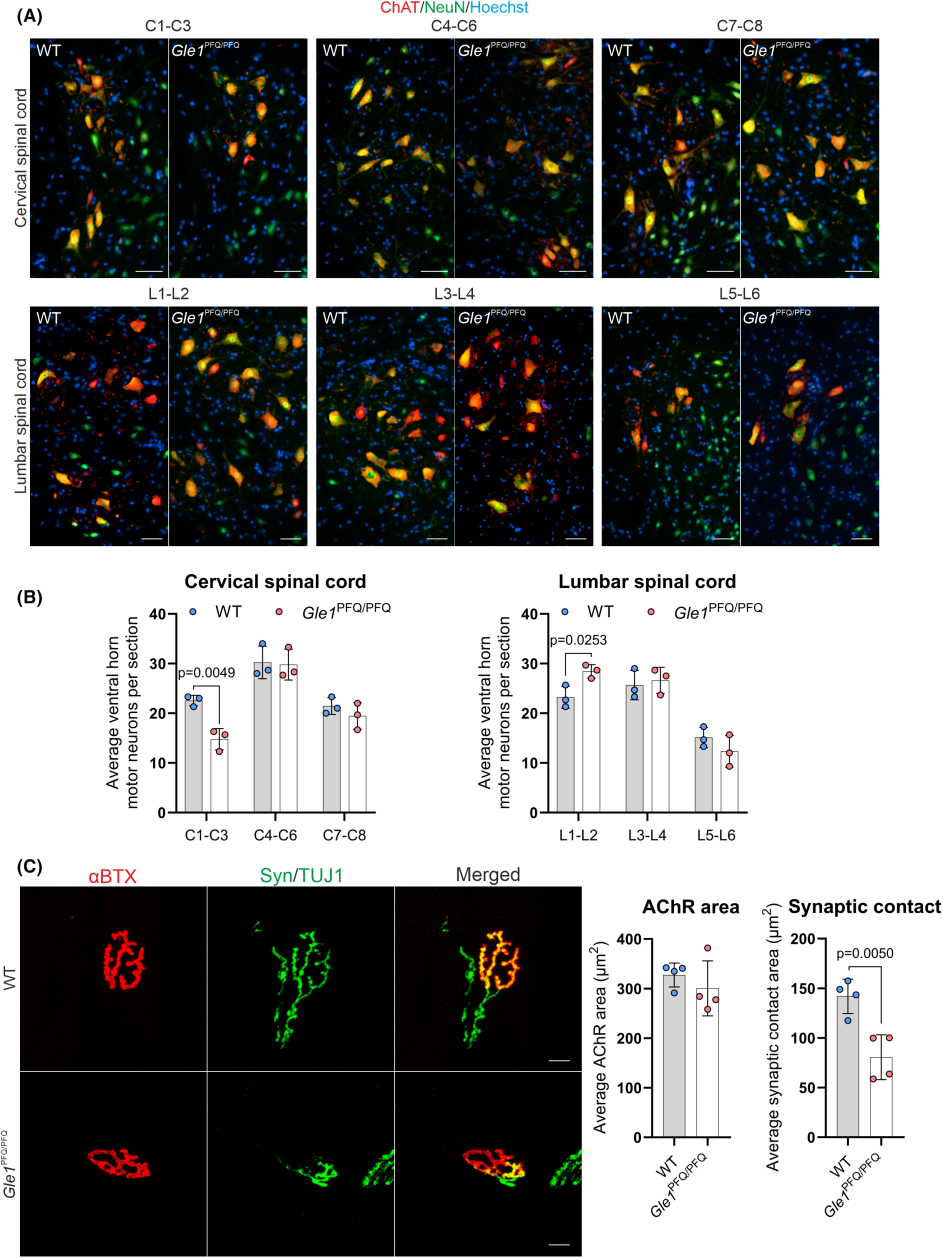
Quantitative analysis of adult *Gle1* ^PFQ/PFQ^ spinal motor neurons and neuromuscular junctions. (A) Representative images of choline acetyltransferase (ChAT) and neuronal nuclear antigen (Neun) stained ventral horn motor neurons in the distinct segmental levels of cervical (C1–C3, C4–C6, and C7–C8) and lumbar (L1–L2, L3–L4, and L5–L6) spinal cords at the age of 25 weeks. Three mice per genotype, three sections per each specified region of the spinal cord. Scale bar 50 μm. (B) Quantification of the ChAT/NeuN‐positive ventral horn motor neurons in the distinct levels of cervical and lumbar spinal cord. Mean ± SD are plotted (*n* = 3), as well as individual data points generated from each mouse (i.e. the average values from three sections per region), two‐tailed Student's *t*‐test. (C) Gastrocnemius muscle longitudinal sections were stained and visualized, to assess the presynaptic (Tuj1/synaptophysin) and postsynaptic (α‐bungarotoxin) morphology: acetylcholine receptor (AChR) area and the size of synaptic contact of neuromuscular junctions (NMJs) in 35 weeks old mice. Mean ± SD are plotted (*n* = 4), as well as individual data points generated from each mouse (i.e. the mean values from 40 NMJs), two‐tailed Student's *t*‐test. Scale bar 20 μm. WT, wild‐type.

To see if the embryonic alterations in motoneurons are projected into adult morphology, we scrutinized the spinal ventral horn MN count in 25‐week‐old mice. Analysis of adult spinal cords at (C1–C3, C4–C6, and C7–C8) and lumbar (L1–L2, L3–L4, and L5–L6) levels identified a small decrease in ChAT/NeuN‐positive MNs in C1–C3 regions and an increase in L1–L2 regions (Fig. 7A,B). For full functional capacity, MNs make connections with muscles via neuromuscular junctions (NMJs), which control signaling between the presynaptic nerve ending and the postsynaptic muscle membrane [54, 55]. To assess potential alterations in nerve‐muscle communication, NMJs in the gastrocnemius muscle of WT and KI mice were characterized. Visualization of acetylcholine receptors by α‐bungarotoxin (αBTX) demonstrated comparable NMJ size between WT and *Gle1* ^PFQ/PFQ^ mice, but additionally identified that the *Gle1* ^PFQ/PFQ^ NMJs were severely denervated as seen by the decreased overlay of αBTX‐positive postsynaptic receptors and synaptophysin/TUJ1‐positive presynaptic nerve endings (Fig. 7C). This suggests that the *Gle*1 KI mice do not only have reduced MN count, but also changes reflecting denervation phenotype [56, 57, 58].

Interested if the observed MN and NMJ changes in *Gle1* ^PFQ/PFQ^ mice project into behavioral alterations, we next used a battery of tests (Fig. S9A) to assess the functional changes in *Gle1* KI homozygotes. The experiment started at the age of 10 weeks to avoid the sudden deaths observed at mid‐adulthood and included both males and females with comparable weights (Fig. S9B). The extensive experimentation identified no behavioral differences between WT and *Gle1* ^PFQ/PFQ^ males, but the *Gle1* ^PFQ/PFQ^ females presented a slight increase in light–dark box activity compared to their WT littermates (Figs S9C and S10–S12). From these, we conclude that although the mild MN developmental changes were recapitulated in adult *Gle1* ^PFQ/PFQ^ mice, they were either not severe enough to pass a functional threshold or that the *Gle1* ^PFQ/PFQ^ KI mice have generated compensatory adaptation by the time of tests.

### Adrenal chromaffin cells show a high prevalence in mitosis but are overall diminished in *Gle1* ^PFQ/PFQ^ embryos

**Fig. 8:**
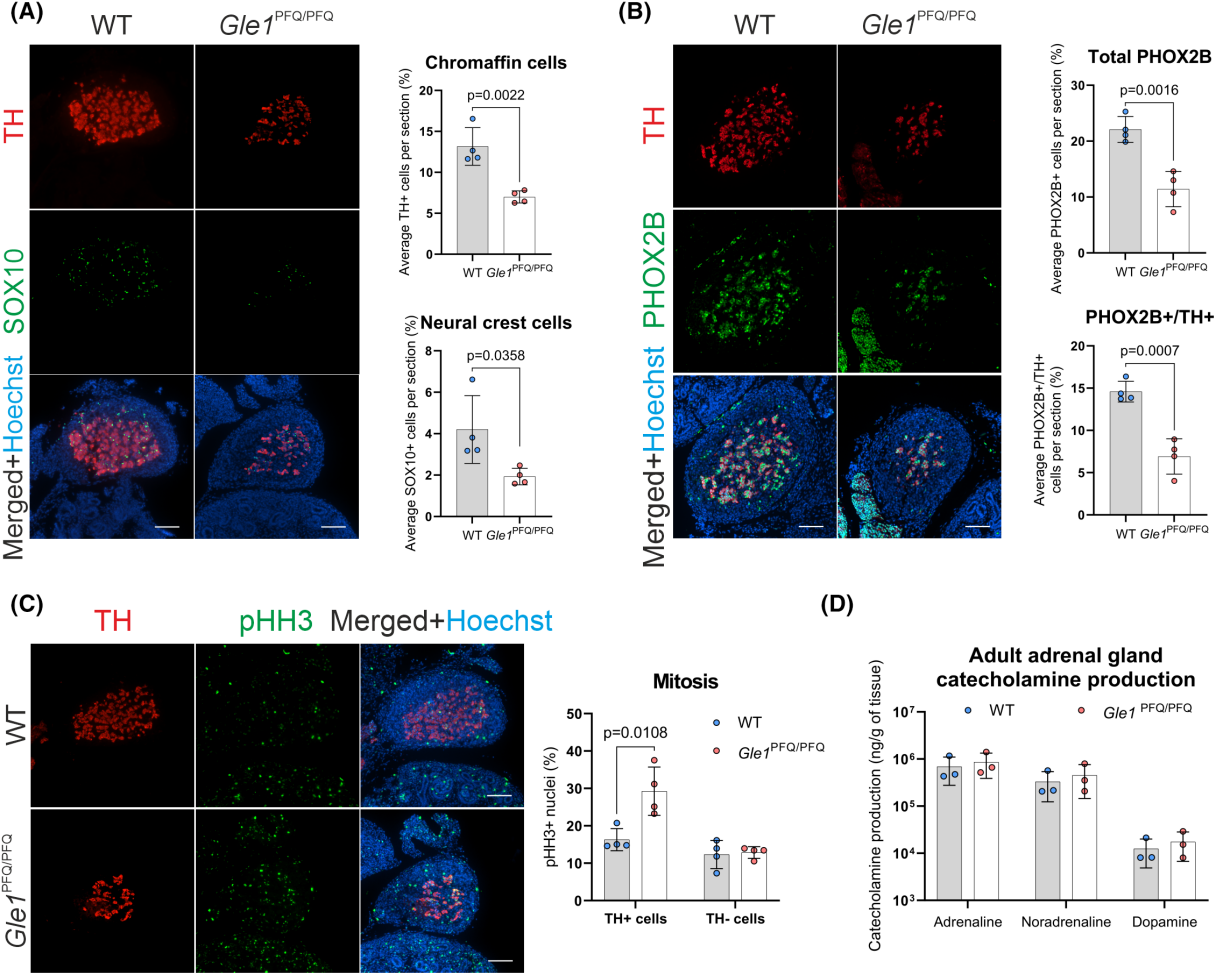
Impaired development of adrenal medulla in *Gle1* ^PFQ/PFQ^ mouse embryos. (A) Representative cross‐sections of E14.5 adrenal glands immunostained for tyrosine hydroxylase (TH, red, chromaffin cells), and SOX10 (green, neural crest cells). Hoechst staining (blue) visualizes all cell nuclei. Mean ± SD are plotted, as well as individual data points generated from each embryo (*n* = 4 per genotype, 3 sections per mouse). Scale bar 100 μm, two‐tailed Student's *t*‐test. (B) Representative cross‐sections of E14.5 adrenal glands immunostained for TH (red, chromaffin cells), and PHOX2B (green, sympathoblasts). Hoechst staining (blue) visualizes all cell nuclei. Mean ± SD are plotted, as well as individual data points generated from each embryo (*n* = 4 per genotype, 3 sections per mouse). Scale bar 100 μm, two‐tailed Student's *t*‐test. (C) Representative cross‐sections of E14.5 adrenal glands immunostained for TH (red, chromaffin cells), phospo‐histone H3 (pHH3, green, mitosis marker) and quantification of mitotic cells in the adrenal gland. Hoechst staining (blue) visualizes all cell nuclei. Mean ± SD are plotted, as well as individual data points generated from each embryo (*n* = 4 per genotype, 3 sections per mouse). Scale bar 100 μm, two‐tailed Student's *t*‐test. (D) High performance liquid chromatography‐mediated quantification of catecholamine (adrenaline, noradrenaline, dopamine) concentration (ng of catecholamine·g^−1^ of adrenal gland) in adult *Gle1* ^PFQ/PFQ^ mouse adrenal glands (*n* = 3 per genotype, both adrenal glands from each mouse collected). Mean ± SD are plotted. WT, wild‐type.

Based on the pathology of LCCS1 fetuses involving skull, chin, and skin abnormalities [59, 60, 61], we hypothesized that in addition to spinal MNs, neural crest‐derived tissues might be affected by the *Gle1* ^PFQ/PFQ^ variant. To test this hypothesis, we studied the adrenal gland chromaffin cells as they are easily accessible and straightforward to analyze in terms of identifying potential defects. Indeed, a statistically significant decrease in tyrosine hydroxylase (TH)‐positive chromaffin cells and SOX10‐positive neural crest cells was detected in *Gle1* ^PFQ/PFQ^ embryos in comparison to WT littermates (Fig. 8A). Furthermore, the developing adrenal gland also harbors PHOX2B‐positive sympathoblasts, a neural crest‐derived cell type capable of producing postganglionic sympathetic neurons and chromaffin cells [62, 63, 64]. This multipotent population demonstrated a decrease in both total PHOX2B‐positive cells and PHOX2B/TH‐double‐positive cells in the developing *Gle1* ^PFQ/PFQ^ adrenal glands (Fig. 8B), further supporting impaired sympathoadrenal development. Analysis of pHH3‐positive chromaffin cell nuclei demonstrated a marked increase specifically in the TH‐positive cells of the *Gle1* ^PFQ/PFQ^ mutants (Fig. 8C) reflecting potential compensatory mechanisms. To determine whether developmental abnormalities in adrenal gland medulla have physiological consequences in adults, catecholamine (adrenaline, noradrenaline, and dopamine) levels were measured in adult mice at 13 weeks of age (Fig. 8D). No differences in catecholamine levels were detected between genotypes. In summary, these findings reinforce the involvement of neural crest dysfunction in the pathogenesis of LCCS1 and expand the range of affected cell types beyond spinal MNs. Lack of adult phenotype likely reflects compensatory mechanisms that restore functional homeostasis postnatally.

### GLE1 function is required for normal development of the sympathetic nervous system

**Fig. 9:**
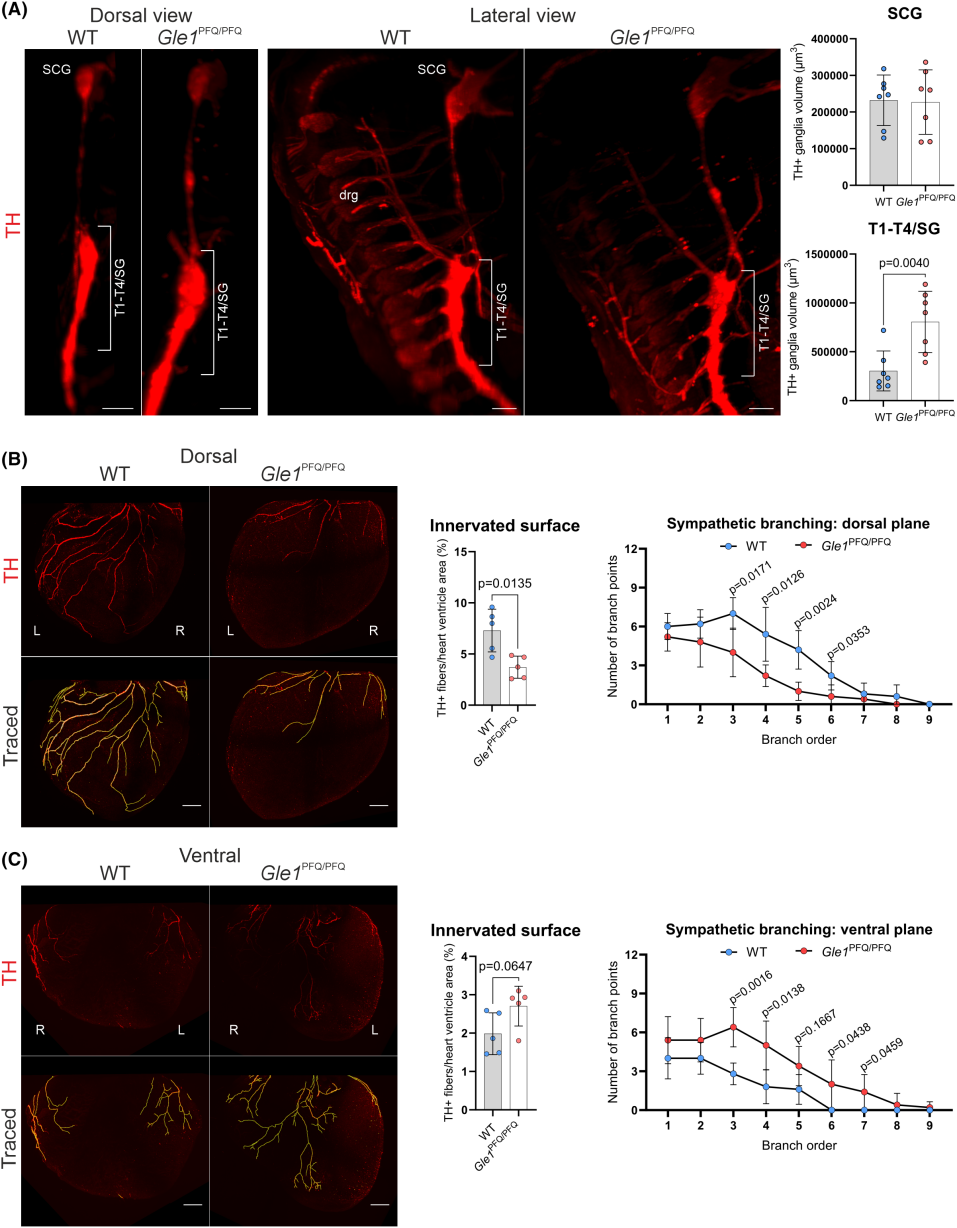
Improper development of cardiac sympathetic innervation *Gle1* ^PFQ/PFQ^ mouse embryos. (A) Whole‐mount tyrosine hydroxylase (TH) immunostaining visualization of the E14.5 paravertebral sympathetic ganglia with focus on the populations contributing to the heart innervation. Both dorsal (left) and lateral (right) views of the stained samples are shown. Quantification of the volume of superior cervical ganglion (SCG), and the block of thoracic (T) T1–T4 and stellate (SG) ganglia (close proximity in early stages, therefore the sympathetic ganglia are distinguished by the position of the nonspecifically stained dorsal root ganglia (drg)). Mean ± SD are plotted, as well as individual data points generated from each embryo (*n* = 7 per genotype). Scale bar 50 μm, two‐tailed Student's *t*‐test. (B) Whole‐mount immunofluorescent staining of tyrosine hydroxylase TH (red)‐positive sympathetic neurons innervating the E16.5 heart ventricles at the dorsal side. Graphs show quantification of innervated surface area and axon branching. Branch points at each order (number of branching events per trunk) were summed across all of the TH‐positive trunks per sample. Shown are mean ± SD, 5 mice per genotype. Scale bar 200 μm, two‐tailed Student's *t*‐test. L, left; R, right; WT, wild‐type. (C) Whole‐mount immunofluorescent staining of tyrosine hydroxylase (TH, red)‐positive sympathetic neurons innervating the E16.5 heart ventricles at the ventral side. Graphs show quantification of innervated surface area and axon branching. Branch points at each order (number of branching events per trunk) were summed across all of the TH‐positive trunks per sample. Shown are mean ± SD, five mice per genotype. Scale bar 200 μm, two‐tailed Student's *t*‐test. L, left; R, right; WT, wild‐type.

Excited about the observations in chromaffin cells that are derivatives of the neural crest but also closely related to sympathetic neurons through sympathoadrenal progenitors [59, 60, 61], we hypothesized that the sympathetic nervous system may be similarly affected in the developing *Gle1* ^PFQ/PFQ^ mice. We have performed whole‐mount imaging of the paravertebral sympathetic ganglia running bilaterally along the spinal cord at the E14.5 stage, which majorly contribute to the sympathetic innervation and control of diverse biological processes, including cardiac output, body temperature, blood glucose levels, and immune function under basal conditions and in response to external stressors such as cold or danger [65, 66]. Indeed, quantification of TH‐positive stellate ganglion and upper thoracic ganglia (SG/T1‐T4) volumes revealed a significant increase in size in the *Gle1* ^PFQ/PFQ^ embryos in comparison to WT controls (Fig. 9A), demonstrating an improper developmental process. The SG/T1‐T4 block of ganglia majorly contributes to the sympathetic innervation of heart ventricles [67, 68]. The heart is a vital organ supplied with multiple neuronal populations densely innervating the myocardium and which shows a high level of neuronal plasticity during injury and disease [69], and we focused on the E16.5 stage as a critical period of sympathetic neurons populating the heart ventricles. Whole‐mount imaging of TH‐positive sympathetic neurons in the dorsal and ventral sides of the ventricles revealed that the innervated surface area in the *Gle1* ^PFQ/PFQ^ hearts was significantly smaller on the dorsal side than in wild‐type embryos, with axon branch points at the 3rd branching order and higher being significantly reduced (Fig. 9B). On the other hand, while no significant changes were observed in the ventral innervated surface area, the number of axon branch points at the 3rd branching order and higher was significantly increased in the *Gle1* ^PFQ/PFQ^ mice (Fig. 9C). This disrupted sympathetic patterning of the heart during embryonic development is particularly concerning, as it can lead to postnatal arrhythmias and sudden death [70, 71].

Axon guidance is a complex symphony of intercellular communication, combining attractive and repulsive signals in temporal and spatial order. Considering the unusual pattern of ventricular innervation, we considered other contributing pathological mechanisms and assessed potential changes in cardiac cell communication. Staining E16.5 heart sections with cellular adhesion (N‐cadherin), axon guidance (Semaphorin 3A), and cardiomyocyte conductivity (Connexin 43) molecules revealed comparable distribution in left and right ventricles of both genotypes (Fig. S13A–C). These results suggest that the abnormal sympathetic ganglia development indeed likely contributes to the aberrant ventricular innervation in our LCCS1 mouse model.

### Adult female *Gle1* ^PFQ/PFQ^ mice present signs of heart failure

Thinking of the premature sudden deaths in the KI mice, we next focused on potential long‐term consequences of the early cardiac innervation defects in the adult hearts. Analysis of laminin α1 (LAMA1) immunostained cardiomyocytes in 25‐week‐old hearts identified increased cardiomyocyte size in the left ventricle of the *Gle1* ^PFQ/PFQ^ mice (Fig. S14). Interested in whether the sympathetic neural network would also be affected in adult hearts, TH‐positive sympathetic neurons counterstained with isolectin B4 (IB4) as an endothelial marker were analyzed. This showed a slight decrease in the TH‐positive area in the left ventricle, suggesting a minor innervation defect, while the IB4‐positive areas presented similar heart vascularization and comparable levels of neurovascular interface in both genotypes (Fig. S15A–C).

**Fig. 10:**
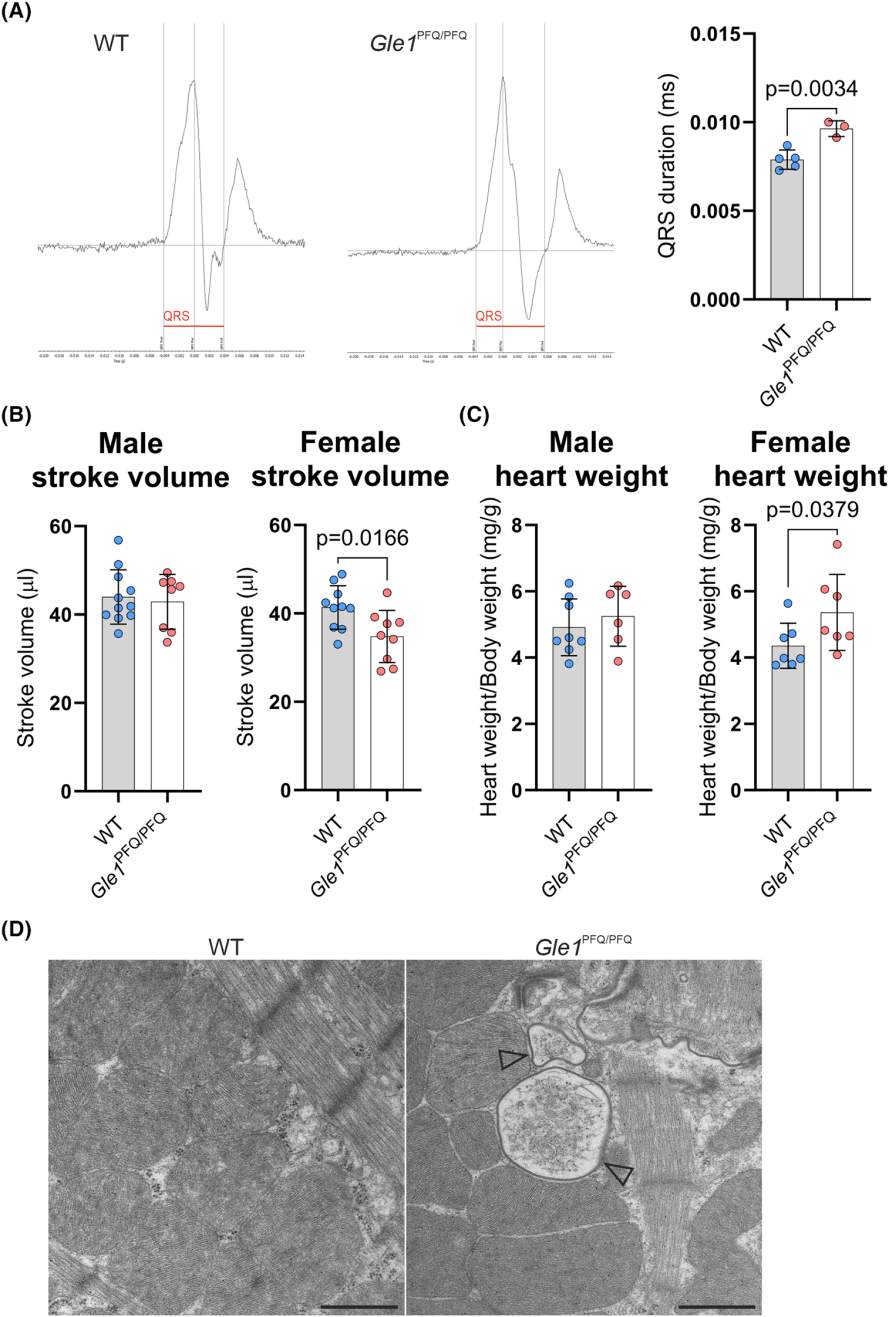
Functional analysis of *Gle1* ^PFQ/PFQ^ adult heart. (A) Representative images of ECG recordings from adult males (8 weeks old). The prolonged QRS wave duration is highlighted by red line. Mean ± SD are plotted, as well as individual data points generated from each mouse (wild‐type, WT males: 5, *Gle1* ^PFQ/PFQ^ males: 3). Two‐tailed Student's *t*‐test. (B) Measurement of left ventricle stroke volume via echocardiography recordings in adult (17–25 weeks old) males and females (WT males: 11, *Gle1* ^PFQ/PFQ^ males: 8, WT females: 10, *Gle1* ^PFQ/PFQ^ females: 9). Two‐tailed Student's *t*‐test. Mean ± SD are plotted. (C) Comparison of heart weight normalized to body weight in adult (17–25 weeks old) males and females (WT males: 8, *Gle1* ^PFQ/PFQ^ males: 6, WT females: 7, *Gle1* ^PFQ/PFQ^ females: 7). Two‐tailed Student's *t*‐test. Mean ± SD are plotted. (D) Representative images from electron microscope analysis of adult mouse left ventricular samples. Arrowheads denote damaged mitochondria (WT females: 3, *Gle1* ^PFQ/PFQ^ females: 3). Original magnifications: 6800×. Scale bar: 1 mm. Mean ± SD are plotted.

To evaluate whether the identified changes affect cardiac function, we first performed electrocardiography (ECG) recordings in WT and *Gle1* KI mice at 8 weeks of age to detect no difference in the female mice, whereas male *Gle1* KI mice presented a longer QRS wave duration than WT males (Fig. 10A, Data S3). On the other hand, ECG analysis at 16 weeks of age showed no difference in any of the ECG parameters between the genotypes in either females or males (Data S4). To provide insights into the autonomous regulation of heart function, heart rate variability (HRV) analysis was performed from the continuous ECG measurements. This identified an increase in the very low frequency (VLF) bands of unknown etiology in the 8‐week‐old females, but no other significant changes (Data S5 and S6). Echocardiography analysis focusing on the systolic function of the left ventricle, which showed increased cardiomyocyte size (Fig. S14), was used next. Analysis of 17–25‐week‐old mice identified a decrease in stroke volume and cardiac output in female *Gle1* ^PFQ/PFQ^ mice but no significant differences in left ventricular systolic function or the structure of the left ventricle (Fig. 10B, Data S7 and S8). Analysis of heart weights also showed an increase in female KI mice, which was absent in males (Fig. 10C, Data S7 and S8).

Characterization of the adult hearts through picrosirius red staining used for myocardial fibrosis detection showed no difference between the genotypes (Fig. S16A), even though the hearts of female KI mice showed a mild increase in the expression of *Col1a1* and *Ccn2* genes that are associated with fibrosis and tissue remodeling [72, 73] (Fig. S16B). In support of the reduced cardiac output, female KI mice presented an increase in the expression of heart failure‐related genes [74, 75] *Nppa*, *Nppb*, and *Myh6*, while this was not seen in male mice (Fig. S16C). Electron microscopy characterization of female *Gle1* ^PFQ/PFQ^ hearts, which exhibited functional and molecular defects, showed occasional mitochondrial defects in otherwise normal cardiac ultrastructure (Fig. 10D). Yet, qPCR analysis did not show any irregular expression of tested mitochondrial modulation related genes [76] suggesting that changes in mitochondrial function or biogenesis do not contribute to the phenotype (Fig. S16D).

To assess if the differentially expressed genes in the *Gle1* KO RNA‐seq dataset are also involved in the LCCS1 disease model, we performed a literary review and picked genes associated with cardiac health and disease, namely *Dag1* [77, 78], *Map4k2* [79], *Bves* [80], *Zic3* [81], *Gfpt2* [82], *Gpr20* [83], and *Kcnv2*, for transcriptional analyses in embryonic (E14.5) and adult *Gle1* KI mouse hearts. We found that the expression of *Gpr20*, *Kcnv2*, and *Map4k2* was beyond the detection limits in embryonic hearts, but *Gfpt2* and *Zic3* showed significantly lower expression in the E14.5 *Gle1* ^PFQ/PFQ^ hearts than in wild‐types (Fig. S17A), mimicking the observations in the *Gle1* KO blastocysts. The very same genes presented differential expression in adults as well, but with a clear sexual bias: *Gfpt2* was upregulated in adult male *Gle1* ^PFQ/PFQ^ hearts, and *Zic3* was upregulated in adult female *Gle1* ^PFQ/PFQ^ hearts (Fig. S17B,C).

## Discussion

Defects in nucleocytoplasmic transport factors and nucleoporins are genetic modifiers of disease [84, 85, 86, 87]. Specifically, mutations in *GLE1* are associated with developmental [5] and neurologic disorders [88]. Previous studies show that most of the nucleoporin knockout mice die during embryogenesis or shortly after birth [89]. We report here that *Gle1* inactivation in mouse mimics previous findings and proves the essential nature of nucleocytoplasmic transport in mammalian cells. Our results demonstrate that total loss of GLE1 function results in embryonic lethality at the pre‐implantation period and that this gene is essential for early developmental processes, such as cell proliferation and spatial organization. In essence, *Gle1* KO blastocysts fail to converge the inner cell mass into a unified population that would localize in one polarized spot of the embryo as seen in controls.

Transcriptional profiling of the *Gle1* ^−/−^ embryos identified ion channels and transporters as the main molecular functions affected by *Gle1* inactivation. Electrical properties of the plasma membrane and alterations in the function as well as distribution of ion channels are known to modulate developing embryos from gamete maturation to neuronal differentiation and function [90]. Our results suggest that early compaction and lineage specification in *Gle1* ^−/−^ embryos occur without major challenges, but the following processes that prepare embryos for implantation and require extensive exchange of adhesive and antiadhesive factors, fluid transport in response to osmotic gradients, and mitosis‐driven paracellular water influx [91] require normal function of GLE1. Differential expression of ion channels, transporters, and adhesion molecules affects their functions but also modulates membrane potential and intracellular signaling via, for example, calcium [92] and is required for survival and thriving.

The severe defects and early lethality of *Gle1* ^−/−^ embryos suggest that even in the most severe *GLE1*‐related developmental disorders, the mutations either modify or disturb the physiological GLE1 functions, rather than induce a complete loss of function. The most severe phenotype among the known genetic GLE1‐related disorders is associated with an intronic *GLE1* variant causing a rare developmental disorder known as LCCS1 [93]. Thus, we genocopied the LCCS1 causative *GLE1*_FinMajor_ variant in mice to produce a *Gle1* ^PFQ/PFQ^ KI model that mimics the documented outcome of *GLE1*_FinMajor_‐induced alternative splicing [5]. The molecular phenotype of the *Gle1* ^PFQ/PFQ^ MEFs indicates that the LCCS1 pathology in mammalian cells may be driven by mechanisms other than simply disrupted nucleocytoplasmic shuttling of poly(A) + RNA [13]. Our results support the importance of GLE1 function in the cellular stress response, as also previously suggested by the studies in HeLa cells [94]. With the emerging trends in stem cell‐derived differentiations, using human pluripotent cells with the intronic *GLE1*_FinMajor_ mutation and pushing them towards differentiation into a more diversified cell types shall be the future strategy to verify the current knowledge and uncover the full spectrum of molecular defects resulting in LCCS1 etiology.

*GLE1* variants enriched in isolated populations are associated with CAAHD and LCCS1 disorders [5]. Perinatal death in CAAHD is caused by postnatal respiratory failure, but the cause of fetal death in LCCS1 remains unknown. LCCS1 fetuses show hydrops, pulmonary and skeletal muscle hypoplasia, micrognathia, and arthrogryposis, defects in anterior horn motor neurons, and severe atrophy of the ventral spinal cord [5]. Our *Gle1* ^PFQ/PFQ^ mouse model presents a milder phenotypic profile and better survivability than what is documented in human patients [95]. It remains to be elucidated whether the mice are less sensitive or more resistant towards the PFQ amino acid insertion due to the interspecies differences in development and physiology [96, 97, 98]. On the other hand, the alternative splicing events induced by the *GLE1*_FinMajor_ mutation may be more complex than what was originally described by Sanger sequencing of the LCCS1 patient cDNA [5]. With the progress in long‐read sequencing methods [99], checking the full *GLE1* transcript length and amino acid composition in LCCS1 patient cells should be the next step for better understanding of molecular biology in LCCS1 disease and to improve the efforts of modeling this disorder.

Even though the *Gle1* ^PFQ/PFQ^ mouse model does not fully phenocopy LCCS1 or other related human diseases, it shows disturbed GLE1 functions at the cellular level and thus provides significant insights into the developmental and physiological roles of GLE1 in mammals. Peripheral nervous system defects are reported for LCCS1 patients, who manifest loss of spinal MNs [4, 5, 6, 7, 8]. We show that *Gle1* ^PFQ/PFQ^ embryos exhibit a similar, though milder, MN defect than human fetuses and additionally have morphologically shifted MN patterning. Beyond the motoric function, the early MN communication with endothelial cells of growing blood vessels is crucial for spinal cord vascularization and blood vessel patterning [100, 101]. Misguided MN patterning can potentially impact the vascular patterning process, affecting the overall integrity of the developing spinal cord. More importantly, we demonstrate that disturbed GLE1 function in the *Gle1* ^PFQ/PFQ^ model results in mid‐adulthood lethality, irregularly developing paravertebral sympathetic ganglia, and adrenal chromaffin cell abnormalities. Our results additionally show that the embryonic heart innervation is abnormal in *Gle1* ^PFQ/PFQ^ mice, which also present mild, sex‐specific phenotypic changes in the adult female hearts. Particularly interesting are the transient irregularities in ECG measurements, which could be explained by compensatory or corrective mechanisms during the cardiac maturation process. It is also possible that the *Gle1* ^PFQ/PFQ^ mice are more susceptible to stress‐induced cardiac arrhythmias (not tested here) leading to sudden death of the mice in mid‐adulthood.

In conclusion, our findings demonstrate the essential nature of GLE1 functions for early mammalian development and suggest that the pathogenic *GLE1* variants rather modify than completely inactive protein function(s). We provided novel information on modified cell‐type specific functions, but further studies especially focusing on protein structure are needed for a better understanding of how protein interactomes are affected by a given pathogenic variant. Additionally, modeling LCCS1 in mice identified that the importance of GLE1 in developmental biology stretches beyond spinal MNs. The future efforts will reveal if these results can be validated in the context of human tissues to enhance understanding of RNA biology in human health and disease.

## Materials and methods

### Mouse generation by CRISPR/Cas9 editing

The FVB/NRj mice were obtained from the Janvier Labs (Le Genest‐Saint‐Isle, France) and used in the study for genome editing. We confirm that all experiments were performed in accordance with relevant guidelines and regulations. The Committee for animal experiments of the District of Southern Finland approved the animal experiments under the license ESAVI/10982/2021 (valid: 29.4.2021–28.5.2024). The Cas9 protein (HiFi Cas9 nuclease, IDT; Leuven, Belgium), gRNA(s), and DNA repair template (Table S1) were microinjected into the zygotes that were further transferred into oviducts of pseudopregnant females. The gRNAs were formed by annealing the crRNA and universal CRISPR‐Cas9 tracrRNA (IDT) and the crRNAs were designed via Benchling (https://benchling.com). The final concentration of reagents for microinjection was 20 ng·μL^−1^ per Cas9 protein, 25 ng·μL^−1^ per gRNA, and 25 ng·μL^−1^ per ssDNA.

#### Generation of *Gle1* KO mice

Two gRNAs were employed to cut out approximately 100 base pairs in exon 1 of the *Gle1* gene. Out of the 467 injected zygotes, five founders were born and verified by Sanger sequencing. The founder, whose deletion induced a reading frameshift and formation of a stop codon in exon 2, was chosen for breeding into the next generations.

#### Generation of *Gle1* KI mice

The combination of gRNA and a ssDNA repair template was employed to directly knock‐in nine nucleotides (coding the PFQ amino acithds) upstream of exon 4 to mimic the outcome of alternative splicing in LCCS1 disease, since the *GLE1*_FinMajor_ is an intronic mutation but mouse and human introns are vastly different. From 54 born pups, 13 *Gle1* ^*em1Skuu*^ founders were identified, 6 of them were verified by Sanger sequencing, and 3 randomly selected founders were bred to the F2 generation. After observing premature lethality of *Gle1* ^*em1Skuu/em1Skuu*^ homozygotes among progenies of all three founders, only one line was randomly selected for further experiments.

#### Genotyping and DNA sequencing

Mouse pups between 2 and 3 weeks were sampled from ear to collect material for genotyping to generate both *Gle1* mouse lines. The DNA was extracted using the Extracta DNA Prep for PCR (95091‐025; Quantabio, Beverly, MA, USA) and used as a template for the PCR reaction, amplified by DreamTaq DNA Polymerase (EP0705; Thermo Fisher, Vilnius, Lithuania) using the SimpliAmp™ Thermal Cycler (A24811; Thermo Fisher, Singapore). Enzyme digestion of the PCR product by BceAI (R0623L; NEB, Ipswich, MA, USA) had been used for the *Gle1* KI mice to distinguish the carriers of the insertion. Genotype was distinguished from the mixture of DNA fragments on SYBR™ Safe (S33102; Thermo Fisher, Carlsbad, CA, USA) stained agarose (BIO‐41025; Bioline, London, UK) gel after electrophoresis. The genotyping primers (Fig. S2) have also been used for Sanger sequencing by employing the Eurofins DNA sequencing services (https://www.eurofins.com). The DNA sequences have been viewed and analyzed through the snapgene software (https://www.snapgene.com).

#### Early embryo genotyping

Either fresh or postfixed embryos after imaging were washed with PBST and lysed in 10 μL of blastocyst lysis buffer (10 mm Tris–HCl, pH 8.0, 50 mm KCl, 2.5 mm MgCl_2_, 0.1 mg·mL^−1^ gelatin, 0.45% Igepal, 0.45% Tween‐20, 0.2 mg·mL^−1^ proteinase K) at +56 °C for 30 min. Proteinase K was inactivated at 95 °C for 10 min. Lysate (2–6 μL) was used as a template for PCR for genotyping.

### Early embryo collection

Six‐ to eight‐week‐old heterozygous females were superovulated by pregnant mare serum – human chorion gonadotrophin (5 IU each) combination [102] and mated with heterozygous males. Embryos (E2.5, E3.5 or E4.5) were flushed out from the oviducts in M2 medium, and either snap‐frozen (RNA isolation), or immediately fixed with 4% PFA for 30 min, or cultured in KSOM medium in a 5% CO_2_ humidified incubator at 37 °C: 2‐cell embryos until E3.25 and E3.5 for 1 day (E3.5 + 1) prior to fixation.

### Spinal cord processing for motoneuron analyses

E11.5 embryos were collected after *Gle1* ^PFQ/+^ heterozygote‐to‐heterozygote mating, fixed in 4% PFA in PBS overnight, and genotyped from the tails. The embryos of desired genotype were cryoprotected in 30% sucrose in PBS for 48 h, before embedding in Tissue‐Tek O.C.T. compound on dry ice. The embryos were cryo sectioned using the Leica CM3050 S cryostat (Leica Biosystems, Deer Park, IL, USA) at the 16 μm thickness and the brachial region spinal cord sections we collected using the anatomical cues.

### RNA extraction

Mouse embryonic fibroblasts were washed with ice‐cold PBS twice, and cells were lysed by TRIzol (15596018; Thermo Fisher) for 5 min on ice. RNA was isolated using the standard phenol–chloroform extraction.

Tissues were quickly dissected, snap‐frozen in liquid nitrogen, and homogenized in TriZol using the Precellys lysing kit (CK28; Bertin Instruments, Rockville, MD, USA). The homogenate was left on ice for 5 min before pushing the content through a 25 G needle several times and leaving it on ice for another 5 min. RNA was isolated using the standard phenol–chloroform extraction.

Blastocysts (E3.5) RNA was extracted with Allprep DNA/RNA Micro kit (#80284; Qiagen, Germantown, MD, USA) according to the instructions. Isolated DNA was used for genotyping.

### Bulk RNA‐sequencing of blastocysts

RNA amplification (SMARTer kit), mRNA library preparation, RNA‐sequencing, and data processing were performed by Novogene (Cambridge, UK) on Illumina Sequencing PE150. Each sample represents a pool of four blastocysts (WT, *n* = 3; KO, *n* = 3). We used R packages txdbmake (1.2.1), genomicfeatures (1.58.0) and rsubread (2.20.0) to extract the genomic location annotations and raw counts from company offered GTF file containing the information about gene location and bam files containing the read alignment to the reference. Raw count matrix and column data were transformed into a DeSeqDataSet. The DeSeqDataSet was filtered to exclude low count genes by only including the genes where a minimum of three samples had a count of 10 or higher. The resulting filtered DeSeqDataSet was used for the differential gene expression analysis using deseq2 (1.46.0) that both normalizes the data and performs differential gene expression analysis. Annotating gene symbols and entrez gene ids was performed after the differential gene expression analysis using R package annotationdbi (1.68.0) and annotation org.mm.eg.db (3.20.0). Results of differential gene expresison were extracted to an excel file. For generating the volcano plot genes with NA *P*‐value and adjusted *P*‐value, genes detected as extreme count outliers by deseq2, were filtered out. Before filtering the data contained 12 240 genes and after filtering 12 083 genes. Adjusted *P*‐value was log_10_ transformed for generating the volcano plot. Volcano plot was generated using R package ggplot2 (3.5.1). Gene Ontology overrepresentation analysis was performed using clusterprofiler (4.14.6). Genes without entrez gene were filtered out. This resulted 58 upregulated genes and 169 downregulated genes that were used together (227 DE genes) as an input for the enrichGO. The background universe genes were set to the deseq2 results filtered to remove missing *P*‐values or missing entrez genes and the final size of the universe was 10 982 genes. *P*‐value cutoff was limited to 1 to obtain maximum results while *q* value cutoff was left to default 0.2. MinGSSize was set to 5 and maxGSSize was set to 1500. For the plotting we used R packages ggplot2 (3.5.1), forcats (1.0.0), dose (4.0.0) and stringr (1.5.1).

R version 4.4.2 (R Foundation for Statistical Computing, Wienna, Austria) and R studio version 2024.12.1 Build 563 (Posit; Boston, MA, USA) were used for all analysis. Genes with adjusted *P*‐value < 0.05 and log_2_(FoldChange) |1| were considered as differentially expressed.

### Reverse transcription‐quantitative polymerase chain reaction (RT‐qPCR)

The cDNA was synthesized using the SuperScript IV Reverse Transcriptase (18090050; Thermo Fisher) and diluted in nuclease‐free water. For the qPCR reaction, 2.5 μL of cDNA was mixed with 1 μL each of forward and reverse primer (Tables S2 and S3) along with 1.5 μL of PCR water and 5 μL SensiFAST™ SYBR® No‐ROX Kit (BIO‐98020; Bioline) or with TaqMan probes (Table S4). qPCR was carried out at CFX384 Touch Real‐Time PCR Detection System (Bio‐Rad; Hercules, CA, USA). qPCR was performed with at least three biological replicates in two technical replicates. The gene expression was normalized to the geometric mean of *Actb*/*Ywhae* or 18S ribosomal RNA and the relative gene expression was calculated using the $2^{-\Delta \Delta C_{T}}$ method [103].

### Western blot

Cells were washed with ice‐cold PBS twice and lysed using 1× RIPA cell lysis solution supplemented with Pierce™ Protease Inhibitor Tablets, EDTA‐free (A32965; Thermo Fisher, Rockford, IL, USA) and Pierce™ Phosphatase Inhibitor Mini Tablets (A32957; Thermo Fisher). The adult heart was snap‐frozen in liquid nitrogen and homogenized in 1× RIPA with the Precellys lysing kit. The homogenate was pushed through the 25 G needle several times, centrifuged at 10 000 r.p.m. for 15 min, and the supernatant was collected for analysis. Protein quantity estimation was performed using Pierce™ BCA Protein Assay Kit (23225; Thermo Fisher). In summary, 12.5 μg heart lysate was used for Gle1 quantification, 7.5 μg of MEFs lysate was used for integrated stress response analysis, and 18 μg of protein was employed for nascent protein synthesis quantification in MEFs. Protein was loaded onto in‐house prepared 10–15% SDS/PAGE separating gels and 6% stacking gel and separation was performed at 110 V at room temperature. Transfer was carried out using the wet blot transfer at 250 mA for 70 min onto the nitrocellulose membrane. The membrane was blocked with 5% BSA solution for 2 h and all the primary antibodies (Table S5) were incubated overnight at 4 °C, while secondary antibodies were incubated for 90 min at room temperature. The detection was performed using the WesternBright ECL HRP substrate on Bio‐Rad ChemiDoc™ Imaging System (Bio‐Rad Laboratories). The proteins were either normalized to β‐actin after the antibodies were stripped away by the Restore™ PLUS Western Blot Stripping Buffer (46430; Thermo Fisher) for 10 min at room temperature and reblocking and restaining, or to the total protein load quantified by the Ponceau S (78376; Merck, Buchs, Switzerland) staining.

### Long range RNA‐sequencing

RNA isolated from P0 WT and *Gle1* ^PFQ/PFQ^ hearts was used for PacBio SMRT sequencing to analyze the presence of any undesired modifications or changes in length of the mouse *Gle1* KI transcript. Sample purity was checked with NanoDrop (ND2000USCAN; Thermo Fisher, Vantaa, Finland) and the RNA integrity with Agilent 2100 Bioanalyzer (Agilent Technologies; Santa Clara, CA, USA). The library has been prepared from 150 μg of RNA using the SMRTbell prep kit guidelines. The quality control, library preparation, and sequencing have been performed by Novogene (Cambridge, UK). Following the sequencing, the demultiplexed files were further processed using PacBio's IsoSeq bioconda package (v.4.0.0) following tool documentation provided by Pacific Biosciences (Menlo Park, CA, USA). In short, any remaining primer sequences were removed from sample specific HiFI‐reads using the lima tool in isoseq mode. Next, ‘isoseq refine’ was used to trim Poly‐A tails and to identify and remove concatamers. Isoform‐level clustering was done using the ‘isoseq cluster2’ function, which was followed by alignment to the reference genome using pbmm2, a A minimap2 SMRT wrapper for PacBio data. The reference genome and annotations used were from GENCODE release M33. ‘Isoseq collapse’ was used to collapse redundant transcripts into unique isoforms using recommended settings for bulk tissue IsoSeq. Pigeon PacBio Transcript Toolkit was then used to prepare the input files and then to classify transcripts against given annotations.

### Tissue collection and processing

#### Paraffin sections

Tissues were collected at the stages indicated in the text. Samples were fixed overnight in 4% paraformaldehyde (PFA), processed, and embedded in paraffin using an automated tissue processor Tissue‐Tek (Sakura; Espoo Finland), cut into 5‐μm‐thick sections, and allowed to dry overnight. Immunofluorescent staining of paraffin sections was performed following a previously published standard protocol [104] with a xylene‐alcohol deparaffination series followed by heat‐induced antigen retrieval in 20 mm Tris–HCl, 1 mm EDTA buffer at pH 8.5. After cooling to room temperature, tissue sections were blocked for 1 h in 50 mm Tris–HCl, 100 mm NaCl, 0.1% Tween‐20 at pH 7.5 (TNT) supplemented with 10% FBS, followed by incubation with primary antibodies at 4 °C overnight, TNT washing, and a 1‐h incubation with secondary antibodies. Additionally, the fluorescent dye Hoechst 33342 (H3570; Thermo Fisher, Eugene, Oregon, USA) was used at a 1 : 1000 dilution for counterstain.

#### Frozen sections

Brachial spinal cord frozen sections were thawed for 5 min at +4 °C and for 10 min at room temperature, followed by washing and rehydration using PBS for 2 × 10 min. Sections were permeabilized for 30 min using 0.5% Triton X‐100 in PBS and blocked for 1 h with 5% FBS in permeabilization buffer. Subsequently, sections were incubated with primary antibodies at 4 °C overnight in blocking buffer, washed in PBS 3×, and underwent 1 h incubation with secondary antibodies in blocking buffer. Additionally, fluorescent dye Hoechst 33342 (H3570; Thermo Fisher) was used at a 1 : 1000 dilution for counterstain.

#### E14.5 limb innervation

The whole forelimb was cut off with scissors, fixed overnight in 4% PFA, permeabilized for 30 min using 0.5% Triton X‐100 in PBS, and blocked for 1 h with 5% FBS in permeabilization buffer. The limbs were incubated in primary antibody, diluted in the blocking buffer, for 48 h at 4 °C. Afterwards, limbs were washed in 0.1% Triton X‐100 in PBS for 3 × 15 min, and incubated in secondary antibody diluted in the blocking buffer overnight at 4 °C. Following up with washing the limbs again, samples were dehydrated with ethanol series (pH = 9, at 4 °C, gently shaking): 30% for 2 h, 50%, 70%, 2 × 100% for 4 h each, finished with 100% ethanol overnight. After dehydration, the samples were transferred to ethyl cinnamate (ECi, 112372; Merck, St. Louis, MI, USA) for at least 6 h before imaging, using the ECi also as a mounting medium.

#### Adult spinal cord

Adult Gle1 KI mice (25 weeks old, both sexes) were transcardially perfused before the spinal cord collection. First, mice were anesthetized with ketamine (1 mg·kg^−1^)/medetomidine (100 mg·kg^−1^) until no pain was felt (no withdrawal reflex). Afterwards, the mice were perfused with heparinized saline (0.9% NaCl, 0.05% NaN_3_, 10 000 U·L^−1^ heparin sodium) through the apex of the left ventricle for 3 min. Subsequently, the saline was replaced with 4% PFA for 20 min. After perfusion, respective cervical and lumbar segments of the spinal cord were dissected, post‐fixed in PFA overnight, cryoprotected in 30% sucrose for 48 h, and cryosectioned at 16 μm thickness. For analysis, three spinal cord sections (several sections apart) from each region were analyzed.

### Immunostaining

#### Early embryos

Fixed embryos were permeabilized with 0.3% Triton X‐100/0.1 m glycine in PBS for 20 min at room temperature and washed with PBS + 0.1% Tween‐20 (PBST). The embryos were blocked with 10% filtered FBS in PBST for 30 min and incubated with primary antibodies diluted in 10% FBS in PBST + 0.2% NaN_3_ at +4 °C overnight. After two washes with PBST, embryos were incubated with secondary antibodies for 2 h at room temperature. After a washing step, nuclei were stained with Hoechst for 8 min. Individual embryos were mounted in an imaging chamber in 30% glycerol in PBS and immediately imaged. A list of antibodies is summarized in Table S6.

#### Mouse embryonic fibroblasts

A total of 20 000 cells were seeded and grown on fibronectin‐coated coverslips until desired confluence. Cells were fixed with 4% PFA in PBS for 20 min at room temperature and washed twice with wash buffer (0.2% BSA in PBS + 0.1% Tween 20). The cells were permeabilized with 0.1% Triton X‐100 + 50 mm Glycine for 15 min, washed with wash buffer three times while on a horizontal shaker, blocked with 5% FBS in 0.1% Triton X‐100 in PBS to block unspecific binding, and incubated with primary antibodies (Table S6) diluted in the blocking buffer overnight at 4 °C. Cells were washed with the wash buffer three times again before incubation with the secondary antibodies diluted in the blocking buffer for 90 min. Following another washing step, the nuclei were stained with Hoechst for 8 min. After washing the cells with PBS, they were mounted on glass slides using the Epredia™ Immu‐Mount™ (9990412; Thermo Fisher, Landsmeer, Netherlands). Optionally, phalloidin staining has been added after the secondary antibody staining by diluting the phalloidin in PBS and staining for 2 h.

#### Heart innervation

E16.5 heart ventricles were dissected, fixed with 4% PFA in PBS overnight, and then immunostained and clarified using a published protocol [105]. First, the hearts were incubated in CUBIC reagent‐1 solution containing 25% (wt/vol) of urea, 25% (wt/vol) of *N*,*N*,*N*,*N*‐tetrakis(2‐hydroxypropyl)ethylenediamine and 15% (wt/vol) Triton X‐100 in water. Tissues were immersed in the solution (600 μL in a 4‐well plate) and gently rocked at 37 °C for 5 h. Hearts were blocked with 5% FBS in PBS, 0.1% Triton X‐100 for 12 h at room temperature and incubated with primary antibody in the blocking buffer overnight at +4 °C. The tissues were washed with 0.5% Tween 20 in PBS for 3 × 10 min before incubating the tissues in secondary antibody in blocking buffer. After washing the hearts for 3 × 10 min again, they were equilibrated in 20% sucrose in PBS overnight. Subsequently, hearts were incubated in CUBIC reagent‐2 (50% sucrose, 25% (wt/vol) of urea, 10% (wt/vol) of triethanolamine, 0.1% triton X‐100) for 1 day in the dark and at room temperature. CUBIC reagent‐2 was also employed as a mounting medium for imaging.

#### Paravertebral sympathetic ganglia

E14.5 embryos were collected and cut beneath the thoracic cavity in half before removing the liver. The upper half of the embryo was fixed with 4% PFA in PBS overnight and then immunostained and clarified using a slightly modified iDISCO protocol [106]. Fixed samples were washed in PBS for 1 h twice, then in 50% methanol (in PBS) for 1 h, 80% methanol for 1 h, and 100% methanol for 1 h twice. Samples were then bleached with 5% H_2_O_2_ in 20% DMSO/methanol (1 vol 30% H_2_O_2_/1 vol DMSO/4 vol methanol, ice‐cold) at 4 °C overnight. After bleaching, samples were washed in methanol for 1 h twice, then in 20% DMSO/methanol for 1 h twice, then in 80% methanol for 1 h, 50% methanol for 1 h, PBS for 1 h twice, and finally in PBS/0.2% Triton X‐100 for 1 h twice before further staining procedures. Pretreated samples were incubated in PBS/0.2% Triton X‐100/20% DMSO/0.3 m glycine at 37 °C overnight, then blocked in PBS/0.2% Triton X‐100/10% DMSO/6% FBS at 37 °C overnight. Samples were washed in PBS/0.2% Tween‐20 with 10 μg·mL^−1^ heparin (PTwH) for 1 h twice, then incubated in primary antibody dilutions in PTwH/5% DMSO/3% FBS at 37 °C for 5 days. Samples were then washed in PTwH for 1 day, then incubated in secondary antibody dilutions in PTwH/3% DMSO/3% FBS at 37 °C for 4 days. Samples were finally washed in PTwH for 2 days before clearing and imaging. Immunolabeled tissues were incubated overnight in 10 mL of 50% v/v tetrahydrofuran/H_2_O (THF) (186562‐12X100ML; Merck). Samples were then incubated for 1 h in 10 mL of 80% THF/H_2_O and twice for 1 h in 100% THF and then in dichloromethane (DCM) (270997‐12X100ML; Merck) until they sank at the bottom of the vial. Finally, samples were incubated in 15 mL of dibenzyl ether (DBE) (108014‐1KG; Merck) until clear (~ 2 h) and then stored in DBE at room temperature. Before imaging, the samples were incubated in ethyl cinnamate for 4 h at room temperature (112372‐100G; Merck).

### Mouse embryonic fibroblast isolation, growth, and functional analyses

Plugged females from *Gle1* ^PFQ/+^ heterozygote to heterozygote mating were euthanized by CO_2_ at the E13.5 stage. The uterus was removed and the embryos dissected in Petri dishes. The head was cut off and the organs removed before washing the embryos in PBS to remove blood as much as possible. The carcasses were minced in PBS into cubes of about 2–3 mm in diameter with scissors. The cubes were incubated in trypsin/EDTA (25200‐056; Thermo Fisher, Paisley, UK) at 37 °C for 30 min with stirring, with additional DNase I (10649890; Thermo Fisher) and trypsin/EDTA added for a further 30‐min incubation. Cell suspensions were centrifuged at 1000 r.p.m. (11.2 g) for 5 min. The cell pellets were washed with MEFs culture medium (DMEM (31966‐021; Gibco, Bleiswijk, Netherlands) + 10% FBS (10500064; Thermo Fisher) + penicillin/streptomycin (15140122; Thermo Fisher, New York, USA) twice to eliminate the trypsin activity and finally resuspended in the culture medium. Living nucleated cells were seeded in 30 mm tissue culture dishes and expanded until passage 2 (P2) before freezing the trypsinized cells in the freezing media (7.5% DMSO and 92.5% fetal bovine serum) until later use. The preserved MEFs with the desired genotype were thawed before the experiment and expanded. All of the experiments with MEFs were performed at passage 4 (P4) and at 60–70% confluence on glass fibronectin (1030‐FN‐05M; R&D Systems, Minneapolis, MN, USA) coated coverslips or plastic culture plates (Starlab; Hamburg, Germany).

#### Poly(A) + RNA distribution

Fluorescent *in situ* hybridization of the poly(A) + RNA has been performed on MEFs grown on fibronectin coated coverslips [107]. Cells were fixed with 4% PFA in PBS for 20 min at room temperature, washed in PBS twice, permeabilized with ice‐cold methanol for 10 min, and then incubated in 70% ethanol at 4 °C overnight. On the next day, the cells were incubated for 5 min at room temperature in wash buffer (10% formamide in 2× saline sodium citrate (SSC)). Then, the coverslips were transferred face down onto a drop of 100 μL of hybridization buffer (10% formamide (AM9342, Thermo Fisher), 2× SSC, 1 mg·mL^−1^ yeast tRNA (AM7119; Thermo Fisher) and 10% Dextran sulfate (J14489; Thermo Fisher, Fair Lawn, NJ, USA) containing 100 nm probe (5′‐Cy3‐oligo‐dT(30); IDT), in a humidified chamber sealed with parafilm and incubated in the dark at 37 °C overnight. After hybridization, the coverslips were transferred face up to a fresh well and washed twice in wash buffer in the dark at 37 °C, for 30 min each wash, and then washed briefly in 1× PBS. Hoechst (10 min) was used as a DNA counterstain.

#### RNA synthesis

Global RNA synthesis was analyzed in MEFs grown on fibronectin‐coated coverslips through metabolic labeling. Cells were treated with 1 mm ethynyl uridine (EU) for 1 h before fixation with 4% PFA in PBS for 20 min. Cells were washed and permeabilized with 0.5% Triton X‐100 in PBS, and click chemistry was performed according to the Click‐iT™ RNA Alexa Fluor™ 594 Imaging Kit (C10330; Thermo Fisher) instructions.

#### Protein synthesis

Global protein synthesis was analyzed through metabolic labeling. Cells were treated with 20 μm O‐propargyl‐puromycin for 30 min before fixation with 4% PFA in PBS for 20 min. Cells were washed and permeabilized with 0.5% Triton X‐100 in PBS, and click chemistry was performed according to the Click‐iT™ Plus OPP Alexa Fluor™ 594 Protein Synthesis Assay Kit (C10457; Thermo Fisher) instructions.

### Cell cycle and apoptosis analyses

#### Flow cytometry

Mouse embryonic fibroblasts were grown in 10 cm plates until 60–70% confluence before washing with PBS and trypsinization (15090046; Thermo Fisher). Cells were fixed by slowly adding ice‐cold 70% ethanol and kept at 4 °C for 4 h. Cells were centrifuged (500 r.p.m. for 5 min) and the pellet was washed twice with 2% FBS in PBS. Cells were treated with 100 μL of RNase A for 1 × 10^6^ cells at a concentration of 1 mg·mL^−1^ and incubated at 37 °C for 30 min. MEFs were stained with propidium iodide (421301; BioLegend, San Diego, CA, USA). Samples were analyzed using the BD Accuri C6 plus Analyzer (BD Biosciences; Milpitas, CA, USA), with the cell population gated in the scatter plot and the cell cycle viewed as FL3‐H using a linear scale.

#### Proliferation assay

EdU labeling was performed using Click‐iT EdU Alexa Fluor 488 Imaging Kit (C10337; Thermo Fisher) according to the manufacturer's instructions. Blastocysts (E3.5) were incubated with 10 μm EdU in KSOM media in a 37 °C incubator (5% CO_2_) for 30 min, while MEFs were incubated for 6 h in the standard growth medium before fixation with 4% PFA in PBS for 30 min.

#### TUNEL assay

DNA damage in the blastocysts (E3.5) was assessed by using the *In Situ* Cell Death Detection Kit, Fluorescein (11684795910; Merck, Mannheim, Germany) according to the manufacturer's instructions.

### SA‐β‐gal staining

Mouse embryonic fibroblasts were seeded at passage 4 on the 6‐well plates and grown for 48 h until 60–70% confluence. The SA‐β‐Gal staining was performed using the Senescence Detection Kit (ab65351; Abcam, Waltham, MA, USA). Cells were washed with PBS once, fixed with the kit fixative for 15 min, and stained with X‐gal (10 μg·mL^−1^) for 12 h at 37 °C (incubator without CO_2_).

### Proteasome 20S activity assay

Activity of the 20S catalytic core was measured by fluorometric assay (MAK172; Merck). MEFs were homogenized in 1× PBS, cellular debris was removed by centrifugation at 13 000 ***g*** for 10 min (4 °C) and the resulting supernatant was used according to the manufacturer's instructions. Fluorescence was measured by EnSpire (PerkinElmer; Waltham, MA, USA) plate reader, and the relative fluorescence intensity was normalized to the total protein concentration (determined by BCA assay).

### Small noncoding RNA‐sequencing

Mouse embryonic fibroblasts were grown in 10‐cm plates until 60–70% confluence (cells collected after growth in normal conditions or after hyperosmotic shock: 400 mm sorbitol for 60 min), washed twice with ice‐cold PBS, and lysed by TRIzol (15596018; Thermo Fisher). Total RNA was extracted by the phenol–chloroform method, and RNA concentration and purity were measured by DS‐11 FX Spectrophotometer.

Library preparation from 170 ng of total RNA was performed according to Lexogen Small RNA‐Seq library prep Reference Guide (Lexogen GmbH, Vienna, Austria). Library quality check was performed using Agilent Bioanalyzer High Sensitivity DNA assay (Agilent Technologies, Waldbronn, Germany) and libraries were pooled based on the concentrations acquired from the assay. The library pools were quantified for sequencing using Collibri Library Quantification kit (Thermo Fisher Scientific, Waltham, MA, USA) and sequenced on the Illumina NovaSeq6000 system using SP flow cell (Illumina, San Diego, CA, USA). Read length for the single‐end run was 101 bp.

### tRNA and miRNA data analysis

#### tRNA

The tsRNAsearch Nextflow pipeline, a specialized analysis tool for tRNAs, is used for sequence alignment and feature counting. It employs a prebuilt mouse tRNA genome based on the GRCm38 release 95. Sequence alignment is performed using star (v.2.5.2b), while read counting is executed with featurecounts (v.2.0.6). tRNA gene read counts for the same genes present at multiple loci were aggregated by summing them. For differential gene expression (DGE) analysis, deseq2 (v.1.44.0) is utilized to identify differentially expressed genes. PCA plots are generated using the deseq2 R package, incorporating all genes. Volcano plots are created using the enhancedvolcano package (v.1.13.2), applying a *P*‐value cutoff of 0.05 and a fold‐change threshold of 0.5. Heatmaps are generated with complexheatmap (v.2.20.0). The adjusted *P*‐value (*P*‐adj) cutoff of 0.05 and a fold‐change threshold of 0.5 is applied to heatmaps.

#### miRNA

The nfcore/smrnaseq (v.1.1.0) pipeline was used to analyze miRNAs, utilizing the miRNA database from miRBase release 22. For differential gene expression (DGE) analysis, deseq2 (v.1.44.0) is utilized to identify differentially expressed mature miRNA genes. PCA plots are generated using the deseq2 R package. *P*‐value cutoff of 0.05 and a fold‐change threshold of 0.5 was used. To investigate the functional implications of differentially expressed miRNAs, Reactome pathway enrichment analysis was performed. Target predictions of the differentially expressed miRNAs were obtained using mirpath v.4.0 [108], incorporating experimentally validated targets from tarbase v.8.0. Target predictions of the differentially expressed miRNAs were obtained using mirpath v.4.0 [108], incorporating experimentally validated targets from tarbase v.8.0. A union of predicted and validated gene lists was used as input for the analysis. Enrichment analysis was conducted using the classic algorithm, and *P*‐values were adjusted for multiple testing using the False Discovery Rate (FDR) correction.

### Catecholamine quantification in adrenal glands

Both adrenal glands were collected from adult mice (13 weeks old, both sexes), and catecholamines (adrenaline, noradrenaline, dopamine) were quantified via HPLC‐ECD approach. The system consisted of a mobile phase (0.1 m NaH_2_PO_4_, pH 3.0, 3.5% v/v MeOH, 450 mg·L^−1^ EDTA and 300 mg·mL^−1^ sodium 1‐octanesulfonate), an electrochemical detector (ESA Coulochem model 5600 with 12 channels) and a reverse‐phase C18 column (Kinetex 150 mm × 4.6 mm, 2.6 μm particle size; Phenomenex; Torrance, CA, USA) which was kept at 45 °C. The frozen adrenal samples were homogenized in 0.5 mL of homogenization solution consisting of six parts 0.2 m HCLO_4_ and one part antioxidant solution containing oxalic acid in combination with acetic acid and l‐cysteine [109]. The homogenates were centrifuged at 20 800 ***g*** for 35 min at 4 °C. The supernatant was removed to 0.5 mL Vivaspin filter concentrators (10 000 MWCO PES; Sartorius Lab Instruments, Göttingen, Germany) and centrifuged at 8600 ***g*** at 4 °C for 35 min. Twenty microliter of the filtrate was injected directly into the HPLC system using a flow rate of 1 mL·min^−1^.

### Adult heart characterization

#### ECG and HRV measurements at 8–25 weeks old mice in both sexes

The anesthetized mouse was placed on a platform with its paws attached to three paw‐sized electrodes. Gel was applied to improve conductivity. One channel ECG signal was measured from the right paw to the left paw (lead II). The ECG system was connected to the laptop with an analog‐toto‐digital transformer with a 10 kHz sampling frequency (Power lab/8SP; ADInstruments; Sydney, New South Wales, Australia) managed with labchart software v.7.3.2 (ADInstruments). Ectopic and abnormally shaped beats were removed from the analysis. ECG samples were analyzed manually by a trained operator, who identified the P‐wave onset and offset, QRS boundaries, and peak amplitudes of P, R, and S waves. For the HRV analysis, ECG was recorded for a 5‐min period. HRV parameters were analyzed and calculated by the labchart software tool “HRV analysis.”

#### Echocardiography

High‐resolution ultrasound system Vevo 2100 (Fujifilm‐Visualsonics, Toronto, ON, Canada) was used for the image acquisition with MD‐550 linear array transducer (40 MHz, 30 μm axial, 90 μm lateral resolution). Transthoracic echocardiography was performed on unconscious, anesthetized mice (isoflurane inhalation, 4% for induction and 1.7% for maintenance) on a warm examination platform. Morphological data (interventricular septum thickness (IVS), left ventricular internal diameter (LVID) and left ventricular posterior wall thickness (LVPW)) were collected and parameters for systolic function were calculated with the formulas: fractional shortening = ((LVID; d‐LVID; s)/LVID; d) × 100, ejection fraction = ((LV Vol; d‐LV Vol; s)/LV Vol; d) × 100, LV Vol = (7.0/(2.4 + LVID)) × LVID3. The image analysis was performed with the vevo lab 5.8.1 software (FUJIFILM, Toronto, Ontario, Canada) by an experienced sonographer blinded to the genotype.

#### Analysis of myocardial fibrosis

To quantify myocardial collagen content, the sections were stained with Picro‐Sirius Red (Picro‐Sirius Red Solution ab246832; Abcam) as described previously [110]. The sections were scanned with a brightfield digital slide scanner (40× objective, NanoZoomer S60; Hamamatsu; Hamamatsu City, Shizuoka, Japan) and the obtained images were saved to an external hard drive. Collagenous tissue was quantified automatically with a specially tailored script within visiopharm software (VISIOPHARM; Hoersholm, Denmark) utilizing Bayesian quadratic discriminant analysis of red‐green contrast to detect and calculate the area of collagenous and noncollagenous tissue.

#### Transmission electron microscopy

Heart samples were fixed in 1% glutaraldehyde and 4% formaldehyde in 0.1 m phosphate buffer, postfixed in 1% osmium tetroxide, dehydrated in acetone, and embedded in Epon LX 112 (Ladd Research Industries; Williston, VT, USA) as described previously [111]. Thin sections were cut with a Leica UC6 ultramicrotome, stained in uranyl acetate and lead citrate, and examined by Tecnai G2 Spirit 120 kV transmission electron microscope. The images were captured using a Quemesa CCD camera (EMSIS GmbH, Muenster, Germany).

### Image acquisition

Early embryos were imaged with Leica TCS SP8 X (z‐stack: 1 μm per step) confocal equipped with the Leica HyD hybrid detector and HC PL APO CS2 63×/1.40 objective at 512 × 512 or 1024 × 1024 format. The NMJs were imaged with Leica TCS SP8 X (z‐stack: 1 μm per step) confocal equipped with the Leica HyD hybrid detector and HC PL APO CS2 63×/1.40 objective at 512 × 512 format. For fluorescence intensity analysis, MEFs were imaged with the Zeiss Axio Imager (z‐stack: 7 sections, 1 μm per step) equipped with Hamamatsu Orca Flash 4.0 LT B&W camera and using the EC Plan Neofluar 40×/1.3 and 63×/1.4 objectives. For stress granule analysis, the stressed out MEFs were imaged via Leica TCS SP8 X (z‐stack: 10 sections, 0.5 μm per step) confocal equipped with the Leica HyD hybrid detector and HC PL APO CS2 63×/1.40 objective at 3144 × 3144 format. Images were then deconvoluted using the HyVolution pipeline. E16.5 heart ventricle whole‐mounts were imaged by the Andor Dragonfly spinning disc microscope (z‐stack: 900 sections, 1 μm per step + tile scan: 2 × 2) with Zyla 4.2 sCMOS camera and Plan Fluor DL 10×/0.3 objective. The images were stitched and deconvoluted in the Leica las x 4.6.0 software. The paravertebral sympathetic ganglia analysis was performed using the LaVision Biotec Ultramicroscope II Light Sheet microscope with the Andor Neo 5.5‐CL3 camera (zoom: 0.8, thickness: 6.69 μm, sheet NA: 0.035, step size: 3.35 μm). Following the SA‐β‐Gal staining, representative images of SA‐β‐Gal‐positive MEFs were acquired with the Leica DMi1 at the 10× magnification. Furthermore, Hoechst‐stained nuclei were imaged and overlayed with SA‐β‐Gal stain visible in the brightfield using the Leica TCS SP8 X with the HC PL APO CS2 10×/0.40 objective. The heart paraffin sections were imaged either with Andor Dragonfly Zyla 4.2 sCMOS camera and Plan Fluor DL 10×/0.3 objective (E16.5 sections) or Zeiss Axio Imager at 40× magnification (adult sections). The E12.5 spinal cords were imaged with Andor Dragonfly Plan Apo λ 20×/0.75 objective (z‐stack: 1 μm per step, 2 × 2 tile scan). The E14.5 forelimbs were imaged with Andor Dragonfly Plan Fluor DL 10×/0.3 objective (z‐stack: 1 μm per step, 2 × 2 tile scan).

### Image analysis

Mouse embryonic fibroblasts have been segmented by cellprofiler 4.2.1 either to measure the mean fluorescence intensity of target structures in subcellular compartments or to quantify the number of stress granules as speckle‐type objects per cell (threshold at least 4 pixels per speckle). Heart innervation was analyzed using the Imaris 10.0 filament tracer plug‐in and normalizing the innervated surface area to the total ventricle surface. Paravertebral sympathetic ganglia and early mouse embryos were segmented and analyzed in Imaris as well. SA‐β‐Gal‐positive MEFs were counted manually, after the segmentation of Hoechst‐positive nuclei, via cell counter plug‐in in fiji. The analysis of heart sections through segmentation and thresholding was performed in fiji. The colocalization of TH and IB4 was performed via jacop [112] plug‐in in fiji.

The E12.5 motoneuron soma coordinates were acquired with respect to the midline using fiji software. To assign *x* and *y* coordinates, the maximal height (from the ventral lower border of the spinal cord under the central canal to the dorsal‐most border of the spinal cord) and width (from the center of the central canal to the most lateral edge) of each hemi‐section were measured for normalization [113, 114]. Contour, and density plots were created using the ‘ggplot2’ package in R‐4.4.1. The NMJs were analyzed via the NMJ‐morph macro for fiji [115].

### Behavioral study

#### Housing

The mice were housed in the individually ventilated plastic cages (groups of 2–6 mice/cage; Mouse IVC Green Line – overall dimensions 391 × 199 × 160 mm, floor area 501 cm^2^; Tecniplast, Buguggiate, Italy) with half of the cage covered by a wire bar food hopper. Air inlet and outlet valves are located in the cage lid, on top of the cage, air change 75 times per hour. Cages were in the rack containing up to 70 cages. The cages contained the following enrichment resources: bedding (aspen chips 5 × 5 × 1 mm, 4HP; Tapvei; Paekna, Estonia), nesting material (aspen strips, PM90L; Tapvei) and aspen bricks (50 × 10 × 10 mm; Tapvei), handling tube (red or transparent, length 100 mm, diameter 45 mm). The lights in the colony rooms were on between 6:00 and 18:00 (12‐h normal light–dark cycle). All behavioral experiments were carried out during the light phase (between 9 and 15), as described previously [116, 117, 118, 119, 120].

#### Elevated plus‐maze

Elevated plus‐maze (Fig. S9C) probes brain regions and mechanisms underlying anxiety‐related behavior. The maze consisted of two open arms (30 × 5 cm) and two enclosed arms (30 × 5 cm) connected by a central platform (5 × 5 cm) and elevated to 40 cm above the floor. Open arms were surrounded by 0.5 cm high edges to prevent accidental falling of mice. The floor of each arm was light gray, and the closed arms had transparent (15 cm high) side‐ and end‐walls. The illumination level in all arms was ~ 150 lx. The animal was placed in the center of the maze facing one of the closed arms and observed for 5 min. The trials were recorded with Ethovision XT17 videotracking software (Noldus Information Technology, Wageningen, the Netherlands) [116]. The latency to the first open arm entry, number of open and closed arm entries (four paw criterion), distance traveled, and the time spent in different zones of the maze were measured. The number of fecal boli was counted after the trial [117].

#### Light–dark box

Light–dark box (Fig. S10A) assesses anxiety, exploratory behavior, and interest in aversive areas. The test was carried out in the open field arena (30 × 30 cm; Med Associates, St. Albans, VT, USA) equipped with infrared light sensors detecting horizontal and vertical activity. The dark insert (non‐transparent for visible light) was used to divide the arena into two halves; an opening (a door with a width of 5.5 cm and height of 7 cm) in the wall of the insert allowed the animal's free movement from one compartment to another. Illumination in the center of the light compartment was ~ 550 lx (bright ceiling lights). The animal was placed in the dark compartment and allowed to explore the arena for 10 min. Distance traveled, number of rearings, latency to enter the light compartment, and time spent in different compartments were recorded by the program. The number of fecal boli was counted by the experimenter after the end of the trial [118].

#### Open field

Open field (Fig. S10B) assesses also anxiety, exploratory behavior, and interest in aversive areas. The mice were released in the corner of a novel open field arena (white floor, transparent walls, 30 × 30 cm; Med Associates). Horizontal and vertical activity (distance moved and rearings, respectively) was recorded by infrared sensors for 30 min (light intensity ~ 150 lx). The peripheral zone was defined as a 6 cm wide corridor along the wall [116].

#### Rotarod

Rotarod tests coordination and balance (Fig. S11A). The accelerating rotarod (Ugo Basile, Comerio, Italy) test was performed on two consecutive days. The mice were given three trials a day with an inter‐trial interval of 1 h. Acceleration speed from 4 to 40 r.p.m. over a 5‐min period was chosen. The latency to fall off was recorded with the cutoff time set at 6 min [119].

#### Hot‐plate

Hot‐plate assesses nociception (Fig. S11C). Standard hot plate (Ugo Basile) was heated to 52 °C, and the mouse was confined there by a Plexiglas cylinder (diameter 19 cm, height 26 cm). The latency to display licking or shaking of the hindpaw was recorded [119].

#### Grip strength

Grip strength analyzes muscle strength and function in different positions (Fig. S11B). A commercially available grip strength meter (Ugo Basile) was used to measure forelimb grip strength in mice. The animal was allowed to grasp a bar and was pulled by the tail. Maximal pulling force (in grams) was recorded when the animal lost its grip on the grasping bar. Five trials were performed with an intertrial interval of 1–2 min [119].

#### Social approach

Social approach shows interaction preference (Fig. S11D). A square box (40 × 40 cm, side walls 50 cm high) was used as an arena under reduced light (25 lux), where two Plexiglas cylinders (8 cm diameter, 10 cm high) were placed in the opposite corners, and an unfamiliar NMRI mouse (sex‐ and age‐matched) was confined in one of the cylinders. The test mouse was allowed to explore the arena for 15 min. The distance traveled in the arena and the time spent exploring the cylinders were measured (tracking the animals by Ethovision XT 17) [119].

#### Olfactory habitation‐dishabituation tests

Olfactory habitation‐dishabituation tests sense of smell (Fig. S12A). For this test, the mice were individually habituated for 15 min in a clean cage (GM500) containing bedding material. The cage was closed with a lid (without food hopper) and the cotton swabs were used to present the odors, inserted into the cage through the opening for the water bottle spout. The following odors were used (in the order of testing): almond, vanilla, urine of the same‐sex unfamiliar mice. The concentrated odors were diluted 1 : 50 (into 50 mL Falcon tube). Each odor was presented in three consecutive trials for a duration of 2 min (inter‐trial interval at least 15 min) and during the trial, the sniffing time was recorded.

#### Acoustic startle reflex

Acoustic startle reflex allows noninvasive measurement of acoustic functions (Fig. S12B). Mice were enclosed in a transparent plastic tube (Ø 4.5 cm, length 8 cm) that was placed in the startle chamber (Med Associates) with a background white noise of 65 dB and left undisturbed for 5 min. Acoustic startle stimuli (20‐ms white noise bursts) were presented in a random order with 8–15 s between the subsequent trials. Altogether, 36 trials with the following noise intensities were randomly applied: 68, 72, 75, 78, 82, 86, 90, 100, 110 dB. The startle response was recorded for 65 ms starting with the onset of the startle stimulus. The maximum startle amplitude recorded during the 65‐ms sampling window was used as the dependent variable and averaged over 4 trials with a given stimulus intensity [120].

### GLE1 protein domain prediction analysis

#### Coiled coil domain

The coiled coil domain prediction analysis was performed from human (NP_001003722.1) and mouse (NP_083199.1) amino acid sequences via DeepCoil [121, 122, 123].

#### Aggregation

The aggregation‐prone domain prediction analysis was performed from human (NP_001003722.1) and mouse (NP_083199.1) amino acid sequences via AggreProt [124] prediction tool.

### Statistics and reproducibility

Statistical analyses were performed using the graphpad prism software version 9.2.0 (Dotmatics; Boston, MA, USA) or R‐4.4.1. Statistical significance was determined by the specific tests indicated in the corresponding figure legends. Only two‐tailed tests were used. All experiments presented in the Article were repeated in three independent biological replicates at least. No statistical methods were used to predetermine sample sizes. Data collection and analysis were not performed blinded.

## Conflict of interest

The authors declare no conflict of interest.

## Author contributions

TZ, SL, SM, and SK designed the experiments. TZ, PS, RH, and SK conceptualized the project. ZS, RV, JM, SS, SB, IM, RKe, MT, VV, PTP, JV, RKi, and F‐PZ conducted the experiments. HL and PM carried out the tRNA sequencing. JK performed RNAseq and BY performed tRNA analyses. TZ, ZS, and RKi analyzed data. TZ and SK wrote and edited the manuscript according to all authors' feedback.

## Supporting information


**Fig. S1.** *Gle1* knockout (*Gle1* ^*−/−*^) blastocyst characterization.
**Fig. S2.** Disrupted cell cycle and cell death in *Gle1* knockout (*Gle1* ^*−/−*^) blastocysts.
**Fig. S3.** E3.5 blastocyst RNA seq analysis.
**Fig. S4.** Characterization of GLE1 transcript and protein in *Gle1* ^PFQ/PFQ^ mice.
**Fig. S5.** Cell properties of the *Gle1* ^PFQ/PFQ^ mouse embryonic fibroblasts (MEFs).
**Fig. S6.** Functional analysis in *Gle1* ^PFQ/PFQ^ mouse embryonic fibroblast (MEFs).
**Fig. S7.** Motor neuron (MN) development in E11.5 *Gle1* ^PFQ/PFQ^ mice.
**Fig. S8.** Quantitative analysis of the developing spinal cord in E12.5 *Gle1* ^PFQ/PFQ^ embryos.
**Fig. S9.** Behavioral analysis of adult *Gle1* ^PFQ/PFQ^ mice I.
**Fig. S10.** Behavioral analysis of adult *Gle1* ^PFQ/PFQ^ mice II.
**Fig. S11.** Behavioral analysis of adult *Gle1* ^PFQ/PFQ^ mice III.
**Fig. S12.** Behavioral analysis of adult *Gle1* ^PFQ/PFQ^ mice IV.
**Fig. S13.** Intercellular communication analysis in E16.5 hearts.
**Fig. S14.** Analysis of adult heart cardiomyocytes.
**Fig. S15.** Characterization of adult heart sympathetic innervation and neurovascular interface.
**Fig. S16.** Molecular analysis of the Gle1^PFQ/PFQ^ adult hearts.
**Fig. S17.** Transcriptional analysis of cardiac changes in Gle1^PFQ/PFQ^ embryonic and adult hearts.
**Table S1.** Oligomers used for microinjections to modify the *Gle1* gene.
**Table S2.** Genotyping and sequencing primers for the Gle1 KO and KI mice.
**Table S3.** List of RT‐qPCR primers.
**Table S4.** List of TaqMan probes.
**Table S5.** List of antibodies used for western blot.
**Table S6.** List of antibodies and probes used for fluorescent staining.

**Data S1.** List of differentially expressed genes from *Gle1* KO blastocyst RNA‐seq analysis.

**Data S2.** List of differentially expressed miRNAs and tRNAs in hypersosmotically stressed *Gle1* KI MEFs.

**Data S3.** Analysis of electrocardiogram (ECG) recordings of *Gle1* KI mice at 8 weeks of age.

**Data S4.** Analysis of electrocardiogram (ECG) recordings of *Gle1* KI mice at 16 weeks of age.

**Data S5.** Analysis of heart rate variability (HRV) from electrocardiogram (ECG) recordings of *Gle1* KI mice at 8 weeks of age.

**Data S6.** Analysis of heart rate variability (HRV) from electrocardiogram (ECG) recordings of *Gle1* KI mice at 16 weeks of age.

**Data S7.** Echocardiography analysis of female *Gle1* KI mice.

**Data S8.** Echocardiography analysis of male *Gle1* KI mice.

## Data Availability

The blastocyst RNA‐seq data (PRJNA1162070), the long‐range RNA‐sequencing results (PRJNA1162399), and small non‐coding RNA‐seq data (PRJNA1247996) have been deposited in NCBI's Sequence Read Archive (SRA).
