# Supplementary material for: In vivo modeling of lethal congenital contracture syndrome 1 suggests pathomechanisms in cellular stress responses
Source: FEBS J. 2025 Jul 17;292(23):6365–99. doi: 10.1111/febs.70195 (PMC12699189; doi:10.1111/febs.70195)
Supplement: Supplementary file 1 — Fig. S1. Gle1 knockout (Gle1 −/− ) blastocyst characterization. Fig. S2. Disrupted cell cycle and cell death in Gle1 knockout (Gle1 −/− ) blastocysts. Fig. S3. E3.5 blastocyst RNA seq analysis. Fig. S4. Characterization of GLE1 transcript and protein in Gle1 PFQ/PFQ mice. Fig. S5. Cell properties of the Gle1 PFQ/PFQ mouse embryonic fibroblasts (MEFs). Fig. S6. Functional analysis in Gle1 PFQ/PFQ mouse embryonic fibroblast (MEFs). Fig. S7. Motor neuron (MN) development in E11.5 Gle1 PFQ/PFQ mice. Fig. S8. Quantitative analysis of the developing spinal cord in E12.5 Gle1 PFQ/PFQ embryos. Fig. S9. Behavioral analysis of adult Gle1 PFQ/PFQ mice I. Fig. S10. Behavioral analysis of adult Gle1 PFQ/PFQ mice II. Fig. S11. Behavioral analysis of adult Gle1 PFQ/PFQ mice III. Fig. S12. Behavioral analysis of adult Gle1 PFQ/PFQ mice IV. Fig. S13. Intercellular communication analysis in E16.5 hearts. Fig. S14. Analysis of adult heart cardiomyocytes. Fig. S15. Characterization of adult heart sympathetic innervation and neurovascular interface. Fig. S16. Molecular analysis of the Gle1PFQ/PFQ adult hearts. Fig. S17. Transcriptional analysis of cardiac changes in Gle1PFQ/PFQ embryonic and adult hearts. Table S1. Oligomers used for microinjections to modify the Gle1 gene. Table S2. Genotyping and sequencing primers for the Gle1 KO and KI mice. Table S3. List of RT‐qPCR primers. Table S4. List of TaqMan probes. Table S5. List of antibodies used for western blot. Table S6. List of antibodies and probes used for fluorescent staining. [file FEBS-292-6365-s004.pdf]

Supplementary figure 1

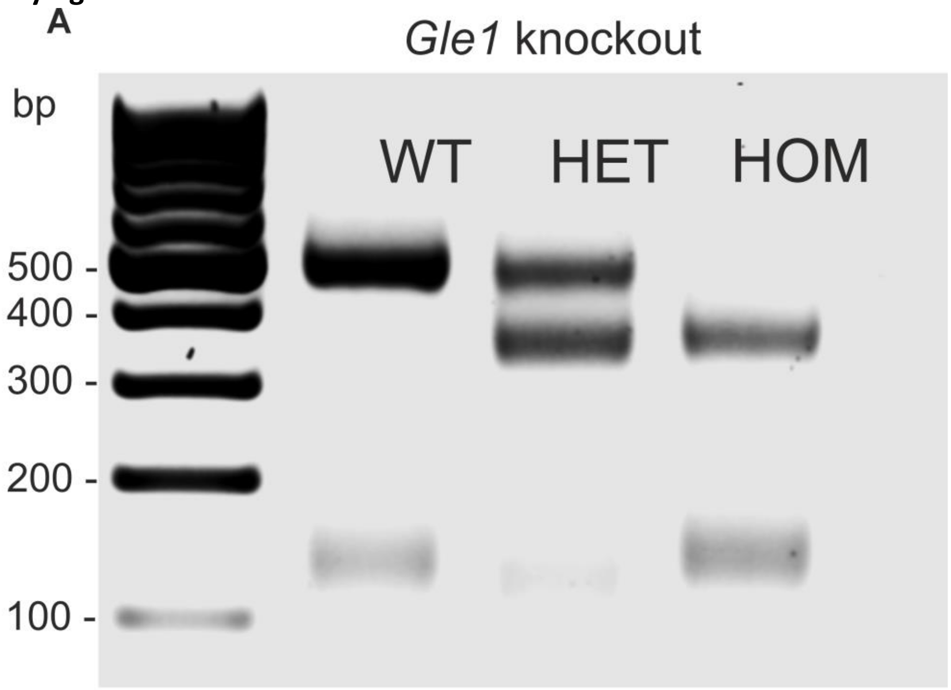

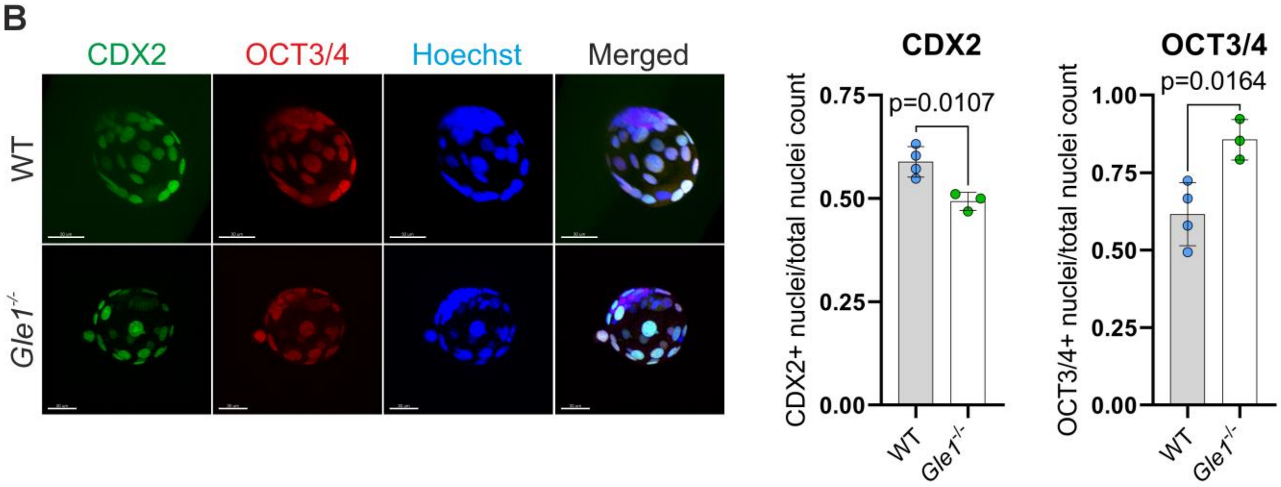

Supplementary figure 1: *Gle1* knockout (*Gle1*$^{-/-}$) blastocyst characterization

A) Comparison of genotyping results via migration of the PCR amplicons, demonstrating the deletion of the *Gle1* gene.

B) Maximum projections of E3.5 embryos flushed from superovulated females, immunostained markers for trophectoderm (CDX2) and inner cell mass marker (OCT3/4) in both WT and *Gle1*$^{-/-}$ early blastocysts. The quantification involves determining the number of CDX2-positive and OCT3/4-positive nuclei relative to the total number of nuclei. Means ± SD are plotted, as well as individual data points generated from each embryo (WT: n=4, *Gle1*$^{-/-}$: n=3). Scale bar 30 µm, two-tailed Student's t test.

Supplementary figure 2

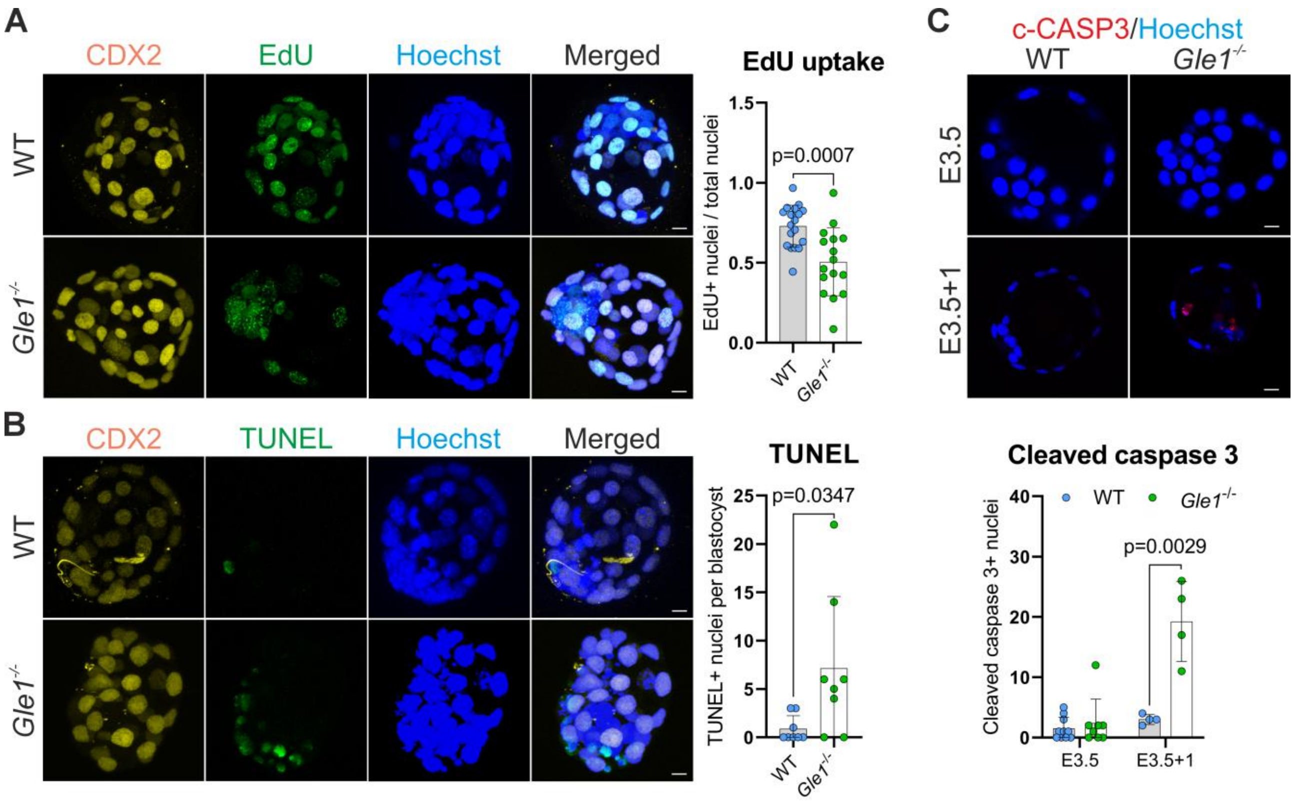

Supplementary figure 2: Disrupted cell cycle and cell death in *Gle1* knockout (*Gle1*$^{-/-}$) blastocysts

A) Proliferation assay of E3.5 blastocysts and quantification of EdU uptake (10 µM EdU, 30 min) as the number of EdU-positive nuclei relative to the total number of nuclei. Means ± SD are plotted, as well as individual data points generated from each embryo (WT: n=18, Gle1$^{-/-}$: n=16). Scale bar 10 µm, two-tailed Student's *t* test.

B) Terminal deoxynucleotidyl transferase dUTP nick end labeling (TUNEL) assay for detecting DNA fragmentation in E3.5 blastocysts. Quantification of DNA damage as the number of TUNEL-positive nuclei per blastocyst. Means ± SD are plotted, as well as individual data points generated from each embryo (WT: n=8, Gle1-/-: n=8). Scale bar 10 µm, two-tailed Student's t test.

C) Cross-section of E3.5 and E3.5+1 cultured blastocysts immunostained for cleaved caspase 3 as a marker of apoptosis. Quantification of apoptotic cells as the number of cleaved caspase 3-positive nuclei per blastocyst. Means ± SD are plotted, as well as individual data points generated from each embryo (E3.5 WT: n=10, *Gle1*$^{-/-}$: n=8; E3.5+1 WT: n=4, *Gle1*$^{-/-}$: n=4). Scale bar 10 µm, two-tailed Student's t test.

Supplementary figure 3

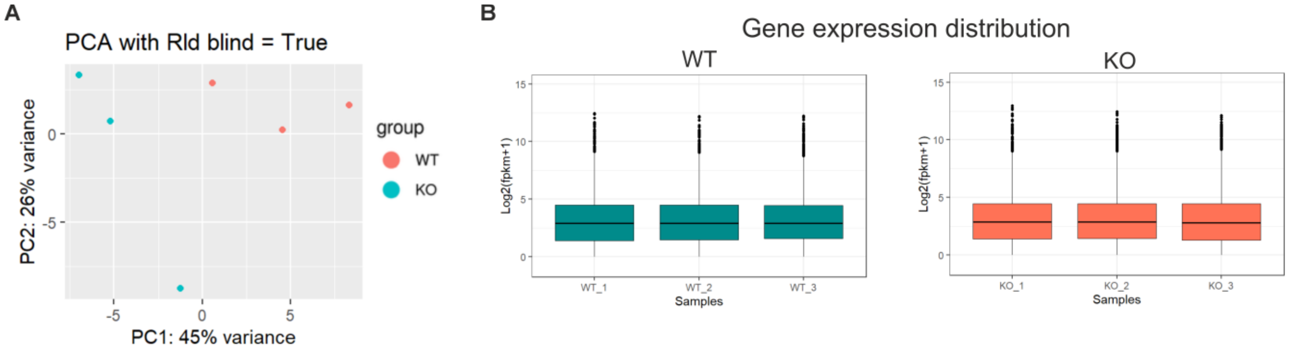

Supplementary figure 3: E3.5 blastocyst RNA seq analysis

A) Principal component analysis of the whole transcriptome. Points represent the whole transcriptomes of WT (red), and *Gle1*$^{-/-}$ (KO) (blue) E3.5 blastocysts. PC1 represents principal component one, and PC2 represents principal component two, both of which explain the first and the second higher variance among embryos.

B) Gene expression distribution boxplot of each sample.

Supplementary figure 4

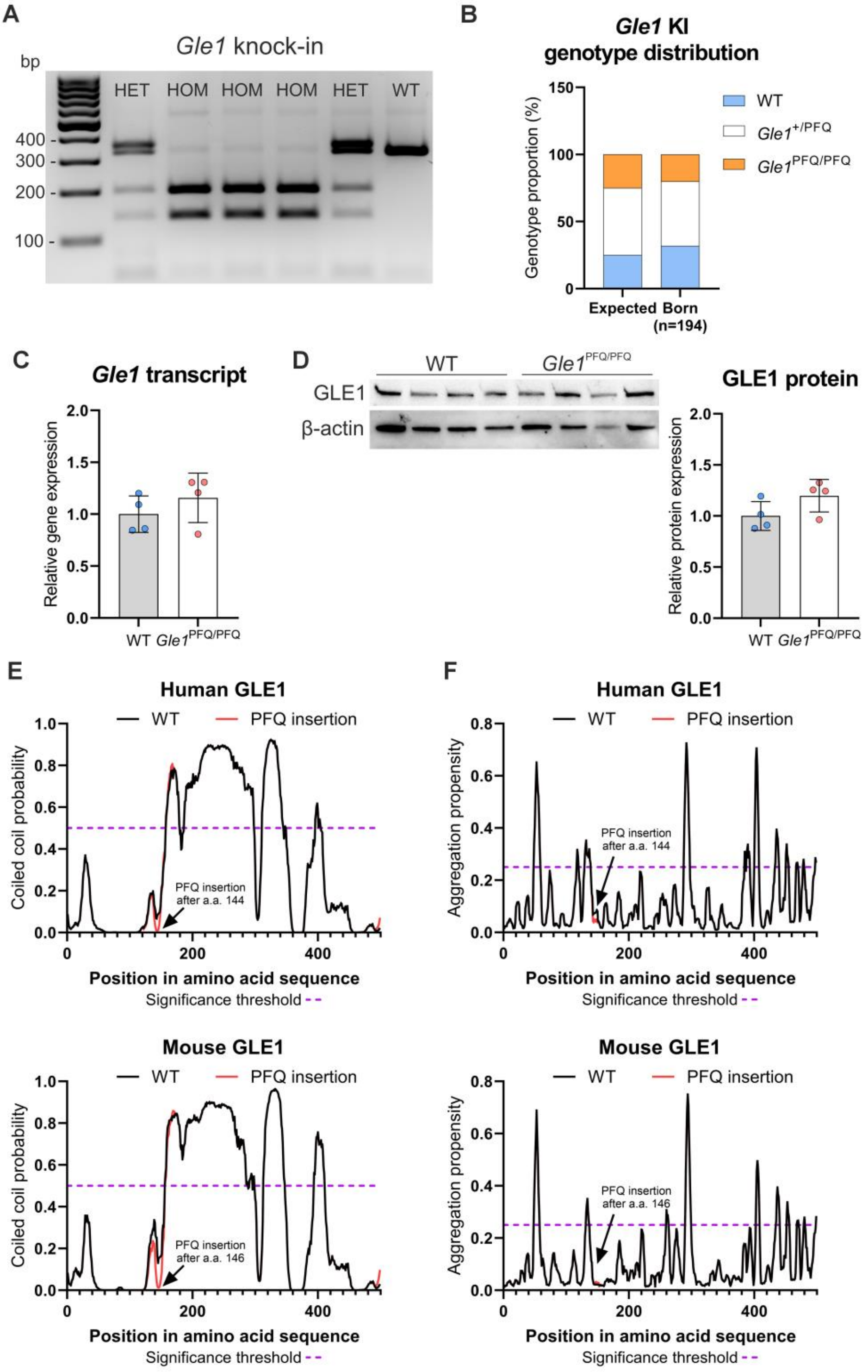

Supplementary figure 4: Characterization of GLE1 transcript and protein in *Gle1*$^{PFQ/PFQ}$ mice

A) Genotyping results of *Gle1* KI mice by PCR followed by amplicon digestion and analysis of the products on agarose gel.

B) Genotype frequencies of mice produced from *Gle1*$^{+/PFQ}$ heterozygote to heterozygote mating (21 litters).

C) RT-qPCR quantification of *Gle1* gene expression in 10-week old adult hearts. Means ± SD are plotted, as well as individual data points generated from each mouse (n=4 per genotype), two-tailed Student's *t* test.

D) Western blot analysis of GLE1 protein expression in adult heart (10 weeks), normalized to the β-actin expression. Means ± SD are plotted, as well as individual data points generated from each mouse (n=4 per genotype), two-tailed Student's *t* test.
E) Bioinformatic prediction of coiled coil domains in human and mouse GLE1 protein, before and after the insertion of PFQ amino acids. Analysis was performed via DeepCoil prediction tool.
F) Bioinformatic prediction of aggregation-prone regions in human and mouse GLE1 protein, before and after the insertion of PFQ amino acids. Analysis was performed via AggreProt prediction tool.

Supplementary figure 5

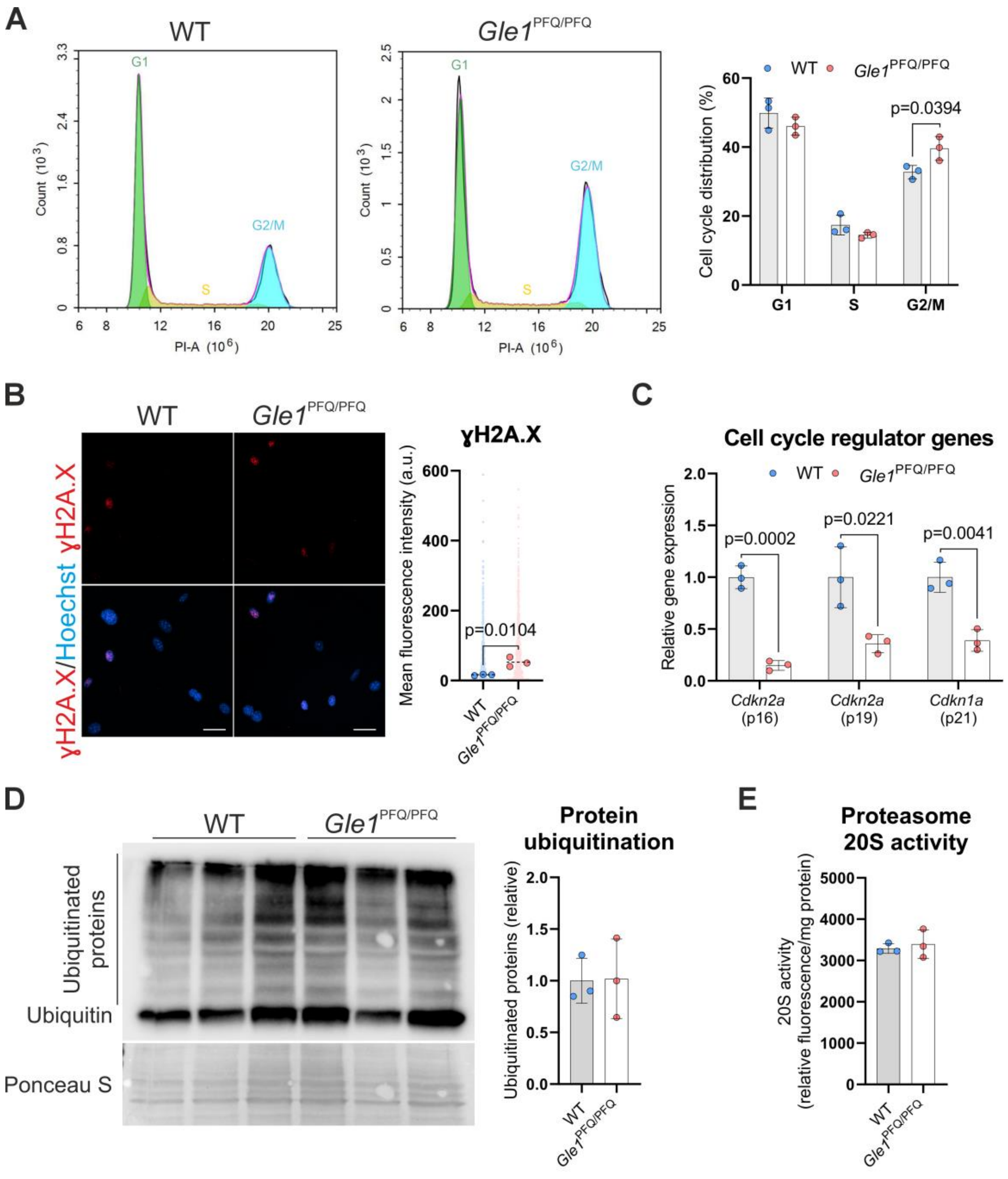

Supplementary figure 5: Cell properties of the *Gle1*PFQ/PFQ mouse embryonic fibroblasts (MEFs)

A) Distribution of the MEFs in the G1, S and G2/M phases of the cell cycle after 48 h by propidium iodide (PI) staining and flow cytometry. Means ± SD are plotted, as well as individual data points generated from each embryo (three embryos per genotype), two-tailed Student's *t* test.

B) Immunostaining and quantification of ɣH2Ax as a marker of DNA damage. Individual data points generated from each cell are plotted (150 cells per embryo, three embryos per genotype), as well as median values from each embryo (large dots). Scale bar 50 µm, two-tailed Student's *t* test.

C) RT-qPCR quantification of *Cdkn2a* (p16 and p19) and *Cdkn1a* (p21) mRNA in MEFs at passage 4. Means ± SD are plotted, as well as individual data points generated from each embryo (three embryos per genotype). Two-tailed Student's *t* test.

D) Quantification of ubiquitinated proteins in MEFs via Western blot analysis (normalized to ponceau S-stained total protein load). Means ± SD are plotted, as well as individual data points generated from each embryo (three embryos per genotype).

E) Quantification of proteasome 20S activity in MEFs via LLVY-R110 as a fluorogenic substrate cultures with MEFs lysates for 1 hour. Means ± SD are plotted, as well as individual data points generated from each embryo (three embryos per genotype).

Supplementary figure 6

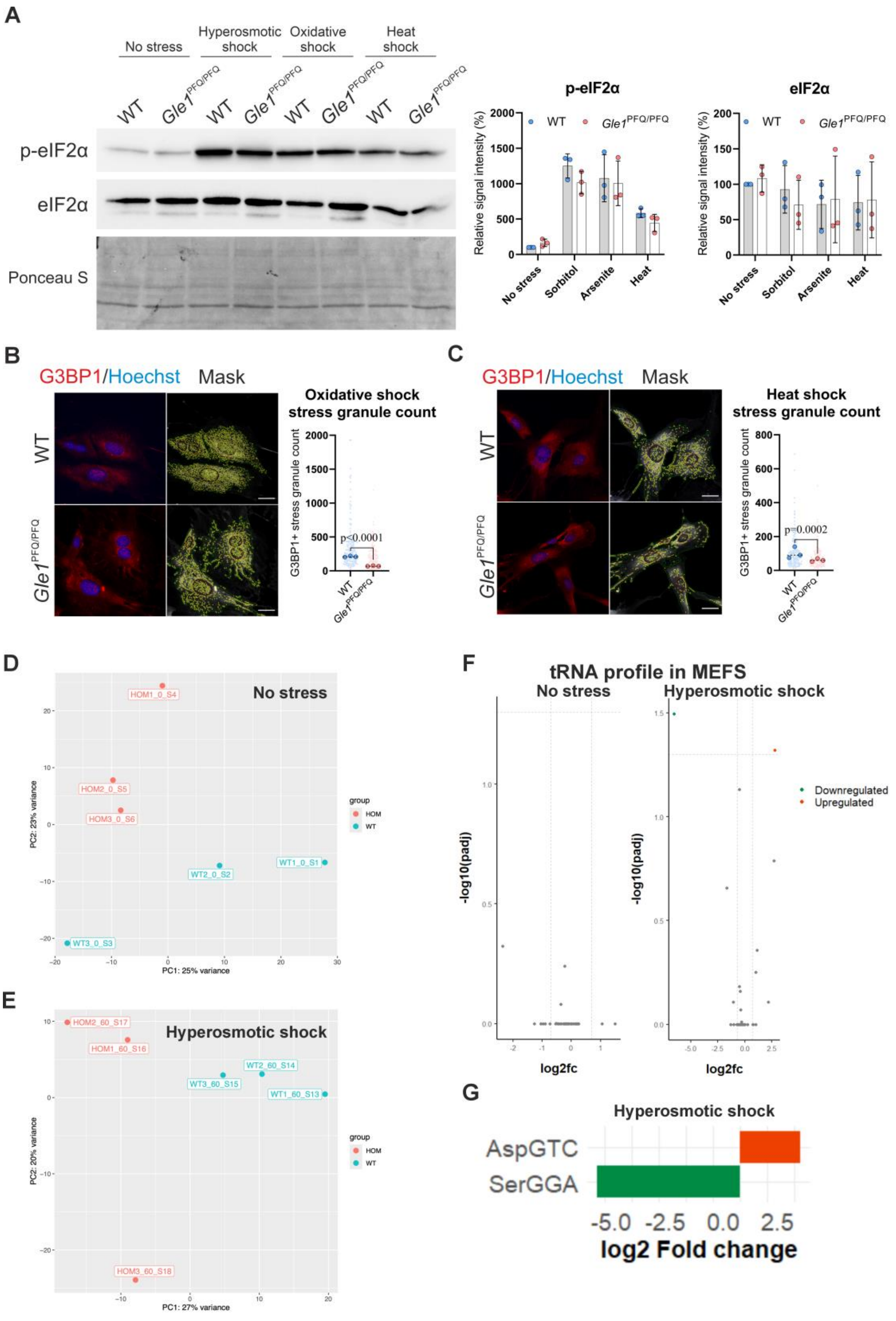

Supplementary figure 6: Functional analysis in *Gle1*$^{PFQ/PFQ}$ mouse embryonic fibroblast (MEFs)

A) The integrated stress response was analyzed by comparing the levels of eIF2α and it phosphorylated form p-eIF2α between wild type and knock-in MEFs upon different stress conditions: no stress vs 60 minutes of hyperosmotic shock (sorbitol)/oxidative shock (sodium arsenate)/heat shock (43 °C), and normalization of the

total protein load (Ponceau S-stained membrane). Means ± SD are plotted, as well as individual data points generated from each embryo (three embryos per genotype).

B) Maximum projection of MEFs after stress challenge with oxidative shock (500µM sodium arsenate, 1 hour) and stress granule quantification as G3BP11 positive speckle type objects (yellow) in the cytoplasm (green) and around the nucleus (red). Individual data points generated from each cell are plotted (50 cells per embryo, three embryos per genotype), as well as median values from each embryo (large dots). Scale bar 30 µm, two-tailed Student's *t* test.

C) Maximum projection of MEFs after stress challenge with heat shock (43°C, 1 hour) and stress granule quantification as G3BP1 positive speckle type objects (yellow) in the cytoplasm (green) and around the nucleus (red). Individual data points generated from each cell are plotted (50 cells per embryo, three embryos per genotype), as well as median values from each embryo (large dots). Scale bar 30 µm, two-tailed Student's *t* test.

D) Principal component analysis of the small non-coding transcriptome in MEFs cultured under normal conditions. Points represent the whole transcriptomes of WT (blue), and *Gle1*$^{PFQ/PFQ}$ homozygote (red) MEFs. PC1 represents principal component one, and PC2 represents principal component two, both of which explain the first and the second higher variance among MEFs.

E) Principal component analysis of the small non-coding transcriptome in MEFs cultured under hyperosmotic shock (400mM sorbitol, 1 hour). Points represent the whole transcriptomes of WT (blue), and *Gle1*$^{PFQ/PFQ}$ homozygote (red) MEFs. PC1 represents principal component one, and PC2 represents principal component two, both of which explain the first and the second higher variance among MEFs.

F) Volcano plots showing differentially expressed tRNAs from small non-coding RNA-seq analysis of *Gle1*$^{PFQ/PFQ}$ MEFs (three embryos per genotype) under no stress or after hyperosmotic shock (400mM sorbitol, 1 hour). Significantly differentially expressed tRNAs (log2 fold change > 0.7; adjusted p-value < 0.05) are indicated in green and orange.

G) Plot of differentially expressed tRNAs in *Gle1*$^{PFQ/PFQ}$ MEFs under hyperosmotic shock (upregulated: orange, and downregulated: green) and their log2 fold change.

Supplementary figure 7

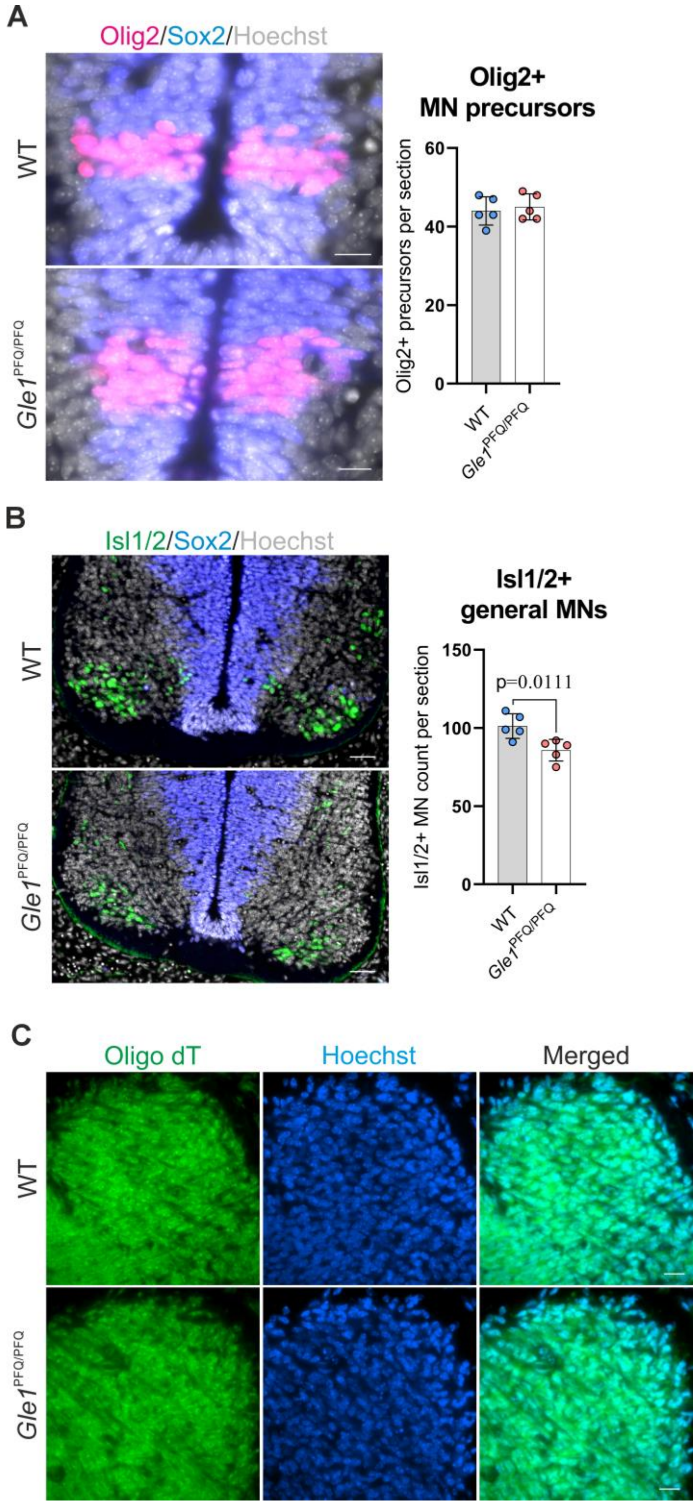

Supplementary figure 7: Motor neuron (MN) development in E11.5 *Gle1*$^{PFQ/PFQ}$ mice

A) Transverse sections through the brachial level of *Gle1*$^{PFQ/PFQ}$ embryos quantified for the immunostained OLIG2-positive progenitors (pink) of MNs from amongst the SOX2-positive neuroepithelial cells (blue) of the neural tube, counterstained with Hoechst (grey). Individual data points generated from each mouse embryo are plotted (average value from 2 analyzed sections per embryo, five embryos per genotype). Scale bar 20 µm, two-tailed Student's *t* test.

B) Transverse sections through the brachial level of *Gle1*$^{PFQ/PFQ}$ embryos quantified for the immunostained Isl1/2-positive general MNs (green) at the lateral motor column, counterstained with SOX2 (blue) and Hoechst (grey). Scale bar = 50µm. Individual data points generated from each mouse embryo are plotted (average value from 2 analyzed sections per embryo, five embryos per genotype). Scale bar 20 µm, two-tailed Student's *t* test.
C) Representative images of oligo dT (green) hybridized lateral motor columns in wild type (WT) and *Gle1*$^{PFQ/PFQ}$ mice, demonstrating normal poly(A)+ RNA distribution between genotypes in the embryonic spinal cords (five embryos per genotype). Scale bar 20 µm

Supplementary figure 8

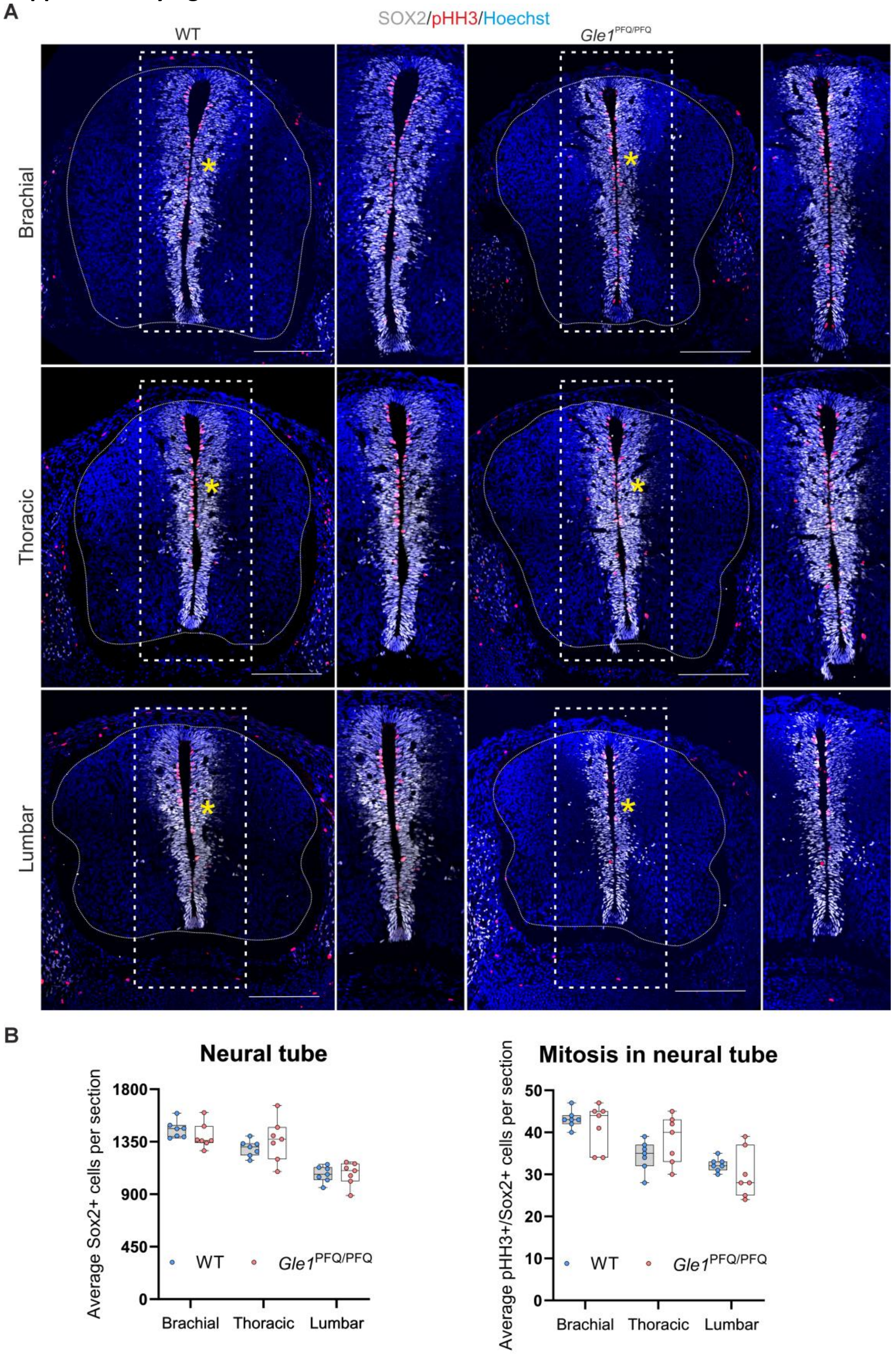

Supplementary figure 8: Quantitative analysis of the developing spinal cord in E12.5 *Gle1*$^{PFQ/PFQ}$ embryos
A) Representative images of E12.5 spinal cord transverse sections at the brachial, thoracic, and lumbar regions, immunolabelled for SOX2 (white) as a marker of neural progenitors constituting the ventricular zone (yellow

asterisk) of the developing neural tube and pHH3 (red) as a marker of the mitotic cells. White boxed area highlights the spinal cord shapes and shows which part of the original images are magnified on right.
B) Quantification of SOX2-positive neural progenitors and pHH3-positive mitotic cells in the neural tube of E12.5 brachial, thoracic, and lumbar spinal cord sections. Data are presented as box and whisker plots, as well as individual data points (n=7) generated from each mouse (average cell count from multiple sections – brachial: 3, thoracic: 4, lumbar: 4), two-tailed Student’s t test Scale bar 200 µm.

Supplementary figure 9

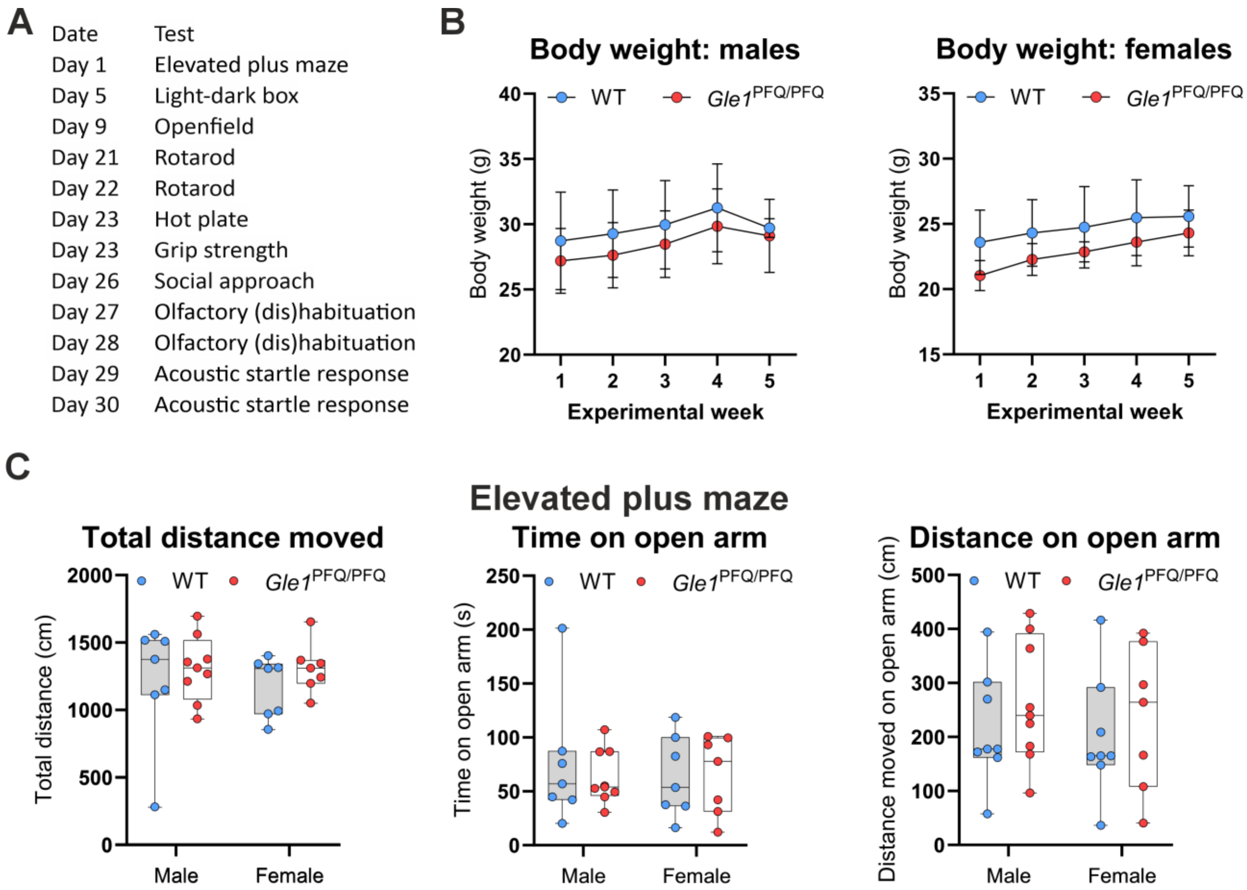

Supplementary figure 9: Behavioral analysis of adult *Gle1*$^{PFQ/PFQ}$ mice I

A) Schedule of the behavioral battery of tests performed on adult (10-14 weeks old) *Gle1*$^{PFQ/PFQ}$ mice.

B) The body weight of the mice, measured during the course of the experiment (WT males: 7, KI males: 8, WT females: 7, KI females: 7).

C) Proportion of time and distance in open arms of the elevated plus-maze (WT males: 7, KI males: 8, WT females: 7, KI females: 7).

Supplementary figure 10

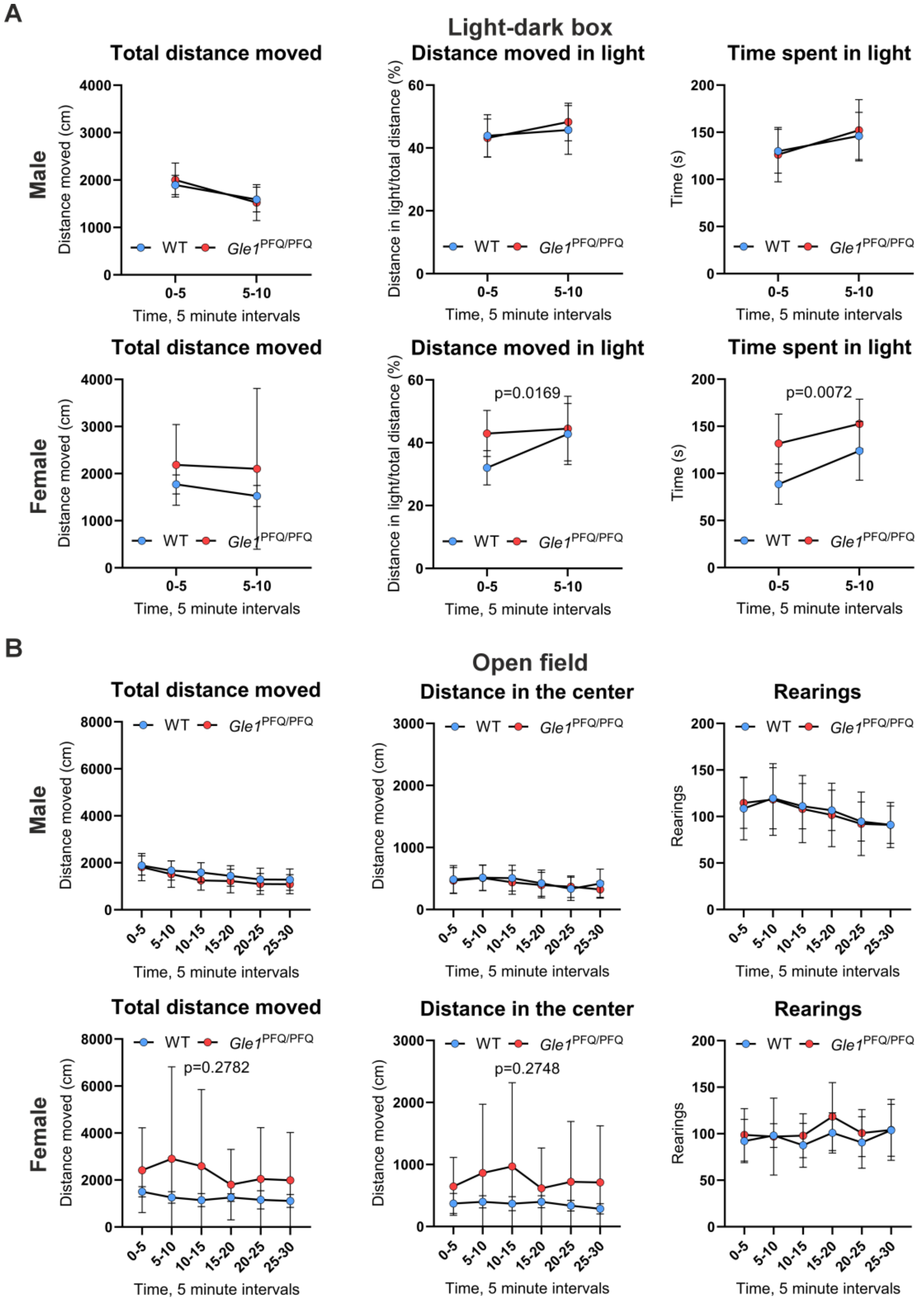

Supplementary figure 10: Behavioral analysis of adult *Gle1*$^{PFQ/PFQ}$ mice II

A) Light-dark test: analysis of time and distance moved in light compartment (WT males: 7, KI males: 8, WT females: 7, KI females: 7). Two-way ANOVA.

B) Distance travelled and exploratory rearing during 30 min in the open field arena (WT males: 7, KI males: 8, WT females: 7, KI females: 7).

Supplementary figure 11

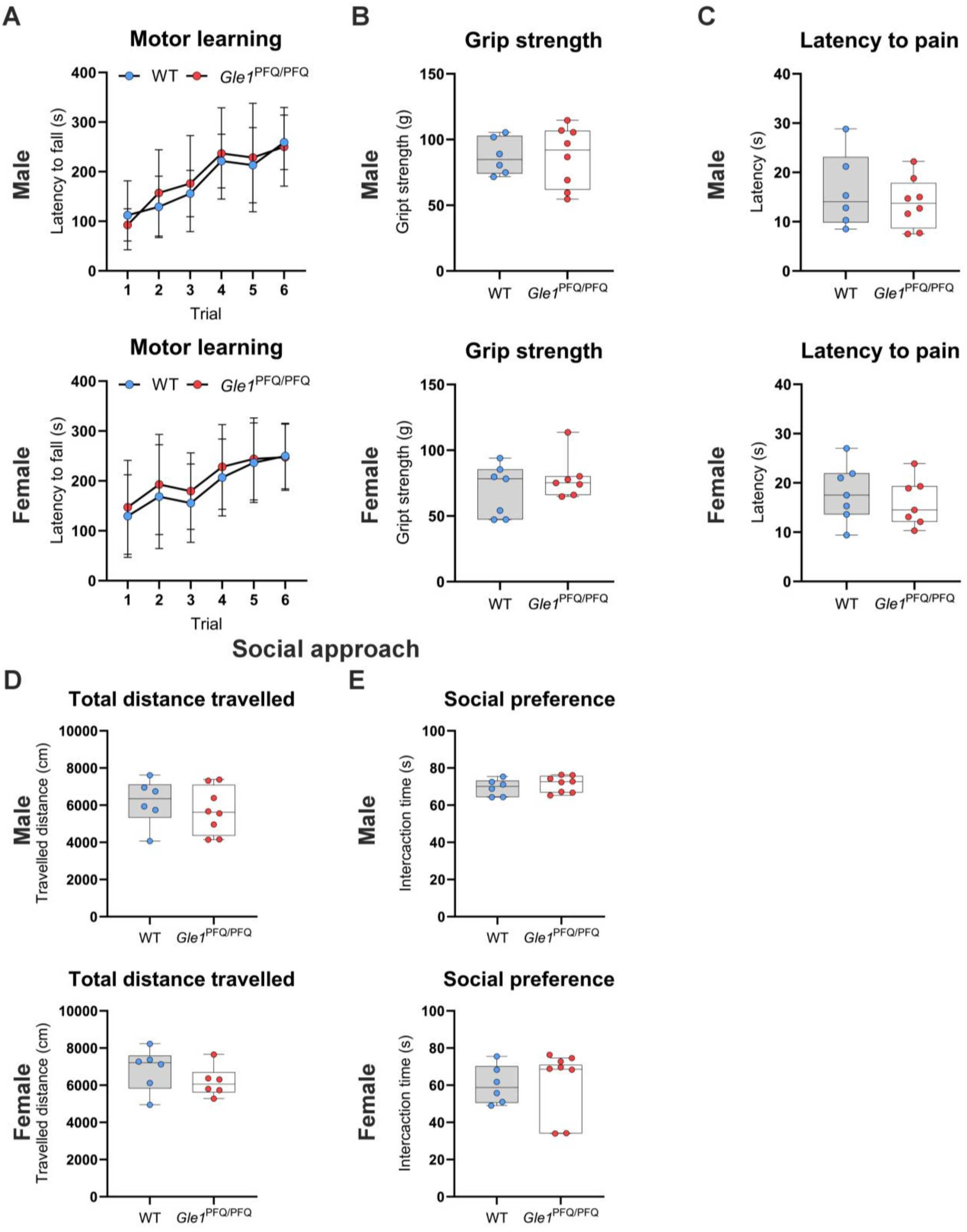

Supplementary figure 11: Behavioral analysis of adult *Gle1*$^{PFQ/PFQ}$ mice III

A) Rotarod: latency to fall (WT males: 6, KI males: 7, WT females: 7, KI females: 7).

B) Grip strength measurement (WT males: 6, KI males: 8, WT females: 7, KI females: 7).

C) Hot plate: latency to pain (males: 6, KI males: 8, WT females: 7, KI females: 7).

D) Social approach: time interaction with social stimulus mouse (males: 6, KI males: 8, WT females: 7, KI females: 7).

Supplementary figure 12

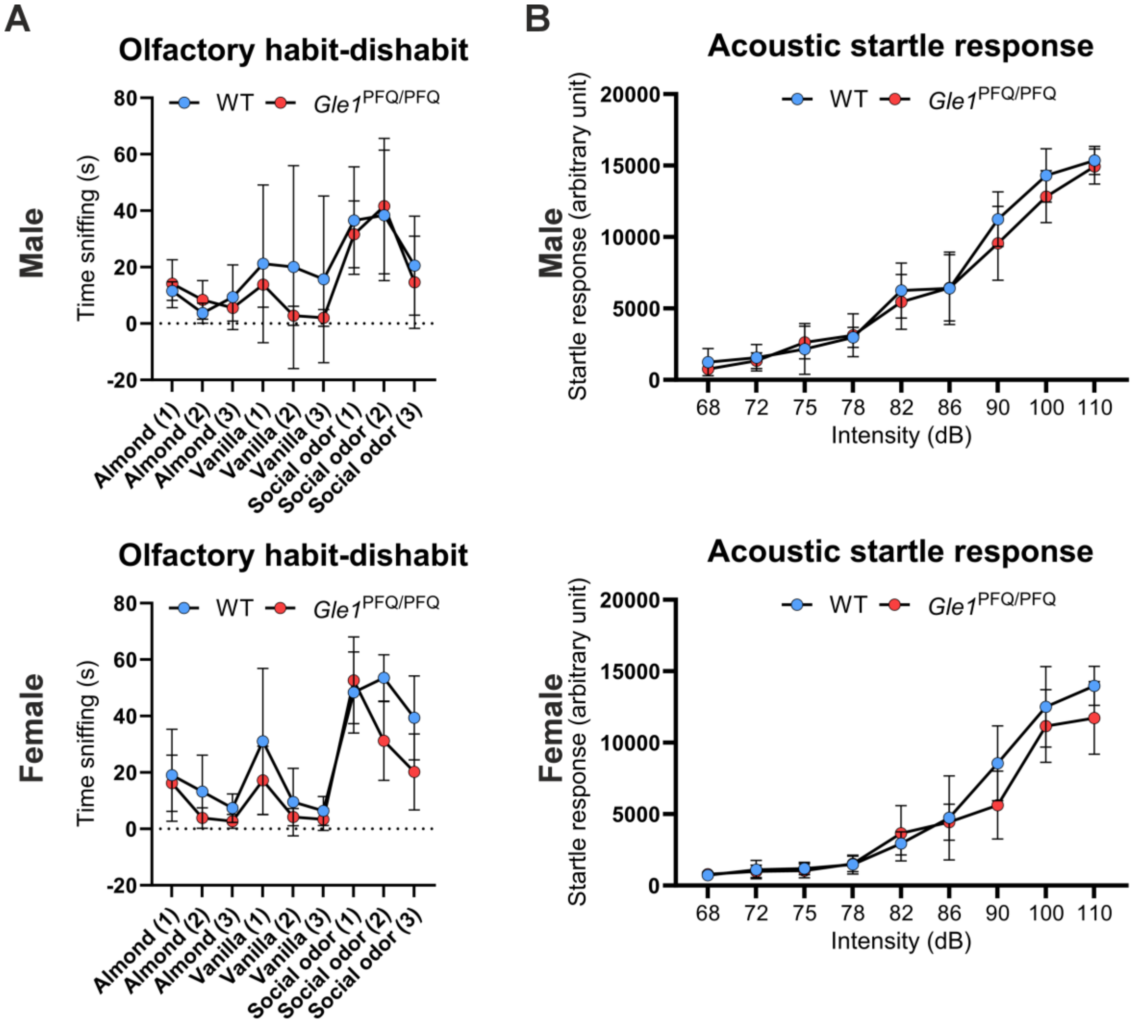

Supplementary figure 12: Behavioral analysis of adult *Gle1*$^{PFQ/PFQ}$ mice IV

A) Olfactory habituation-dishabituation to almond, vanilla, and social odors (WT males: 6, KI males: 8, WT females: 6, KI females: 6).

B) Acoustic startle reflex (WT males: 6, KI males: 8, WT females: 7, KI females: 7).

Supplementary figure 13

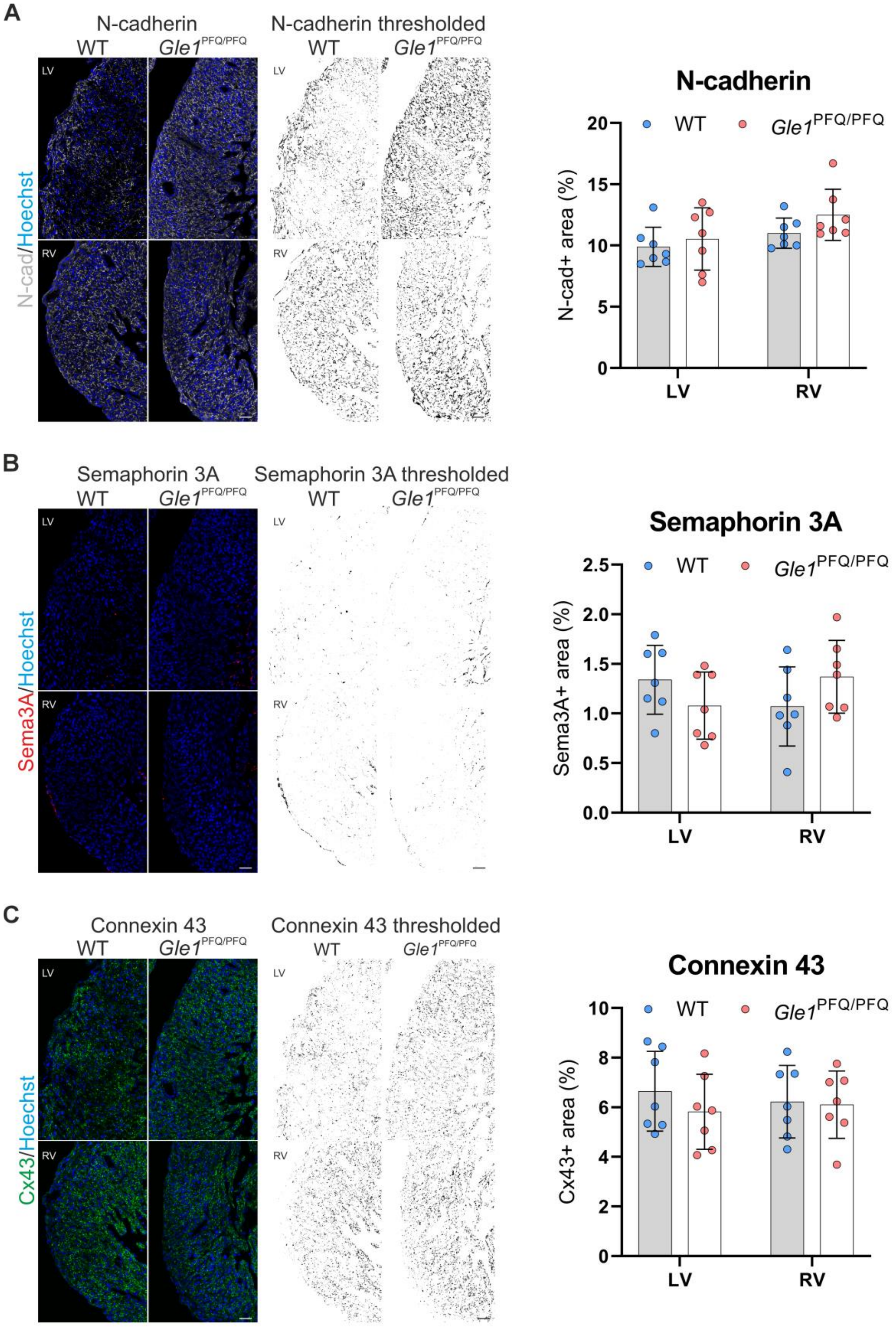

Supplementary figure 13: Intercellular communication analysis in E16.5 hearts

N-CADHERIN **A)**, SEMAPHORIN 3A **B)**, and CONNEXIN 43 **C)** immunofluorescent staining and signal thresholding (right) for ImageJ analysis. Fluorescent-positive area was expressed as percentage of total cross-sectional area of compact myocardium of the left (LV) and right (RV) ventricle in wildtype (WT) and *Gle1*$^{PFQ/PFQ}$ littermates. Means ± SD are plotted, as well as individual data points generated from each embryo (n=7 per genotype). Scale bar 40 µm.

Supplementary figure 14

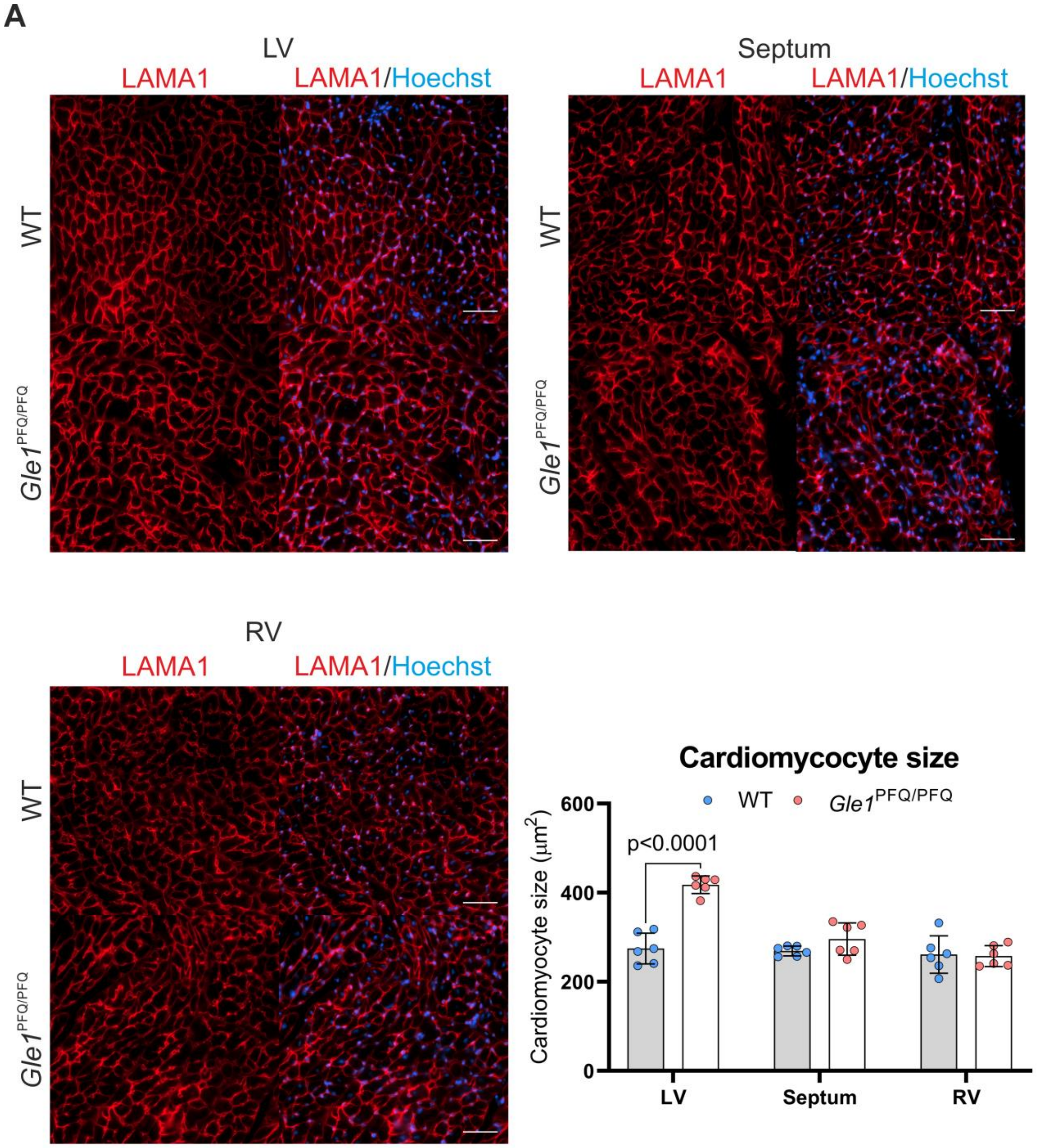

Supplementary figure 14: Analysis of adult heart cardiomyocytes

A) Laminin subunit α1 immunostaining of 25-week adult heart cross sections shown at the mid-level of the left ventricle (LV), septum, and right ventricle (RV). Quantification of cardiomyocyte size in each distinct heart region. Means ± SD are plotted, as well as individual data points generated from each mouse (over 100 cardiomyocytes in 3 fields per region, n=7 per genotype). Scale bar 50 µm, two-tailed Student's *t* test.

Supplementary figure 15

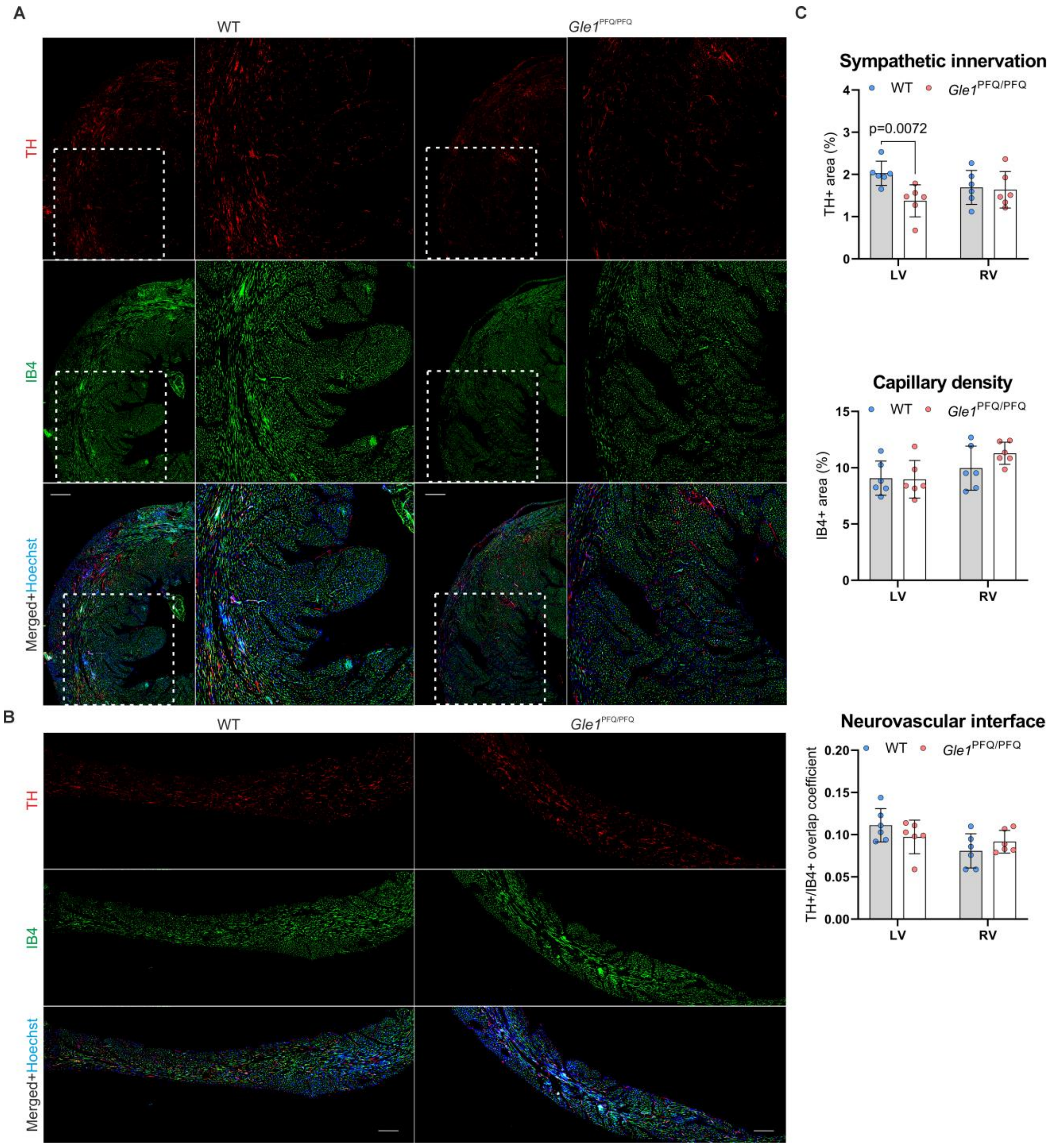

Supplementary figure 15: Characterization of adult heart sympathetic innervation and neurovascular interface

Representative images of 25-week wildtype (WT) and *Gle1*$^{PFQ/PFQ}$ adult heart immunostained for tyrosine hydroxylase (TH, red) and isolectine B4 (IB4, green) in **A)** left ventricles (LV) and **B)** right ventricle (RV). The squared areas in original images are magnified in right. **C)** Quantification of TH-positive area in a single plane as a rate of sympathetic neuron innervation, IB4-positive area as a rate of vascularization, and TH vs IB4 colocalization as a rate of neurovascular interface. Means ± SD are plotted, as well as individual data points generated from mouse (n=7 per genotype). Scale bar 300 µm, two-tailed Student's *t* test

Supplementary figure 16

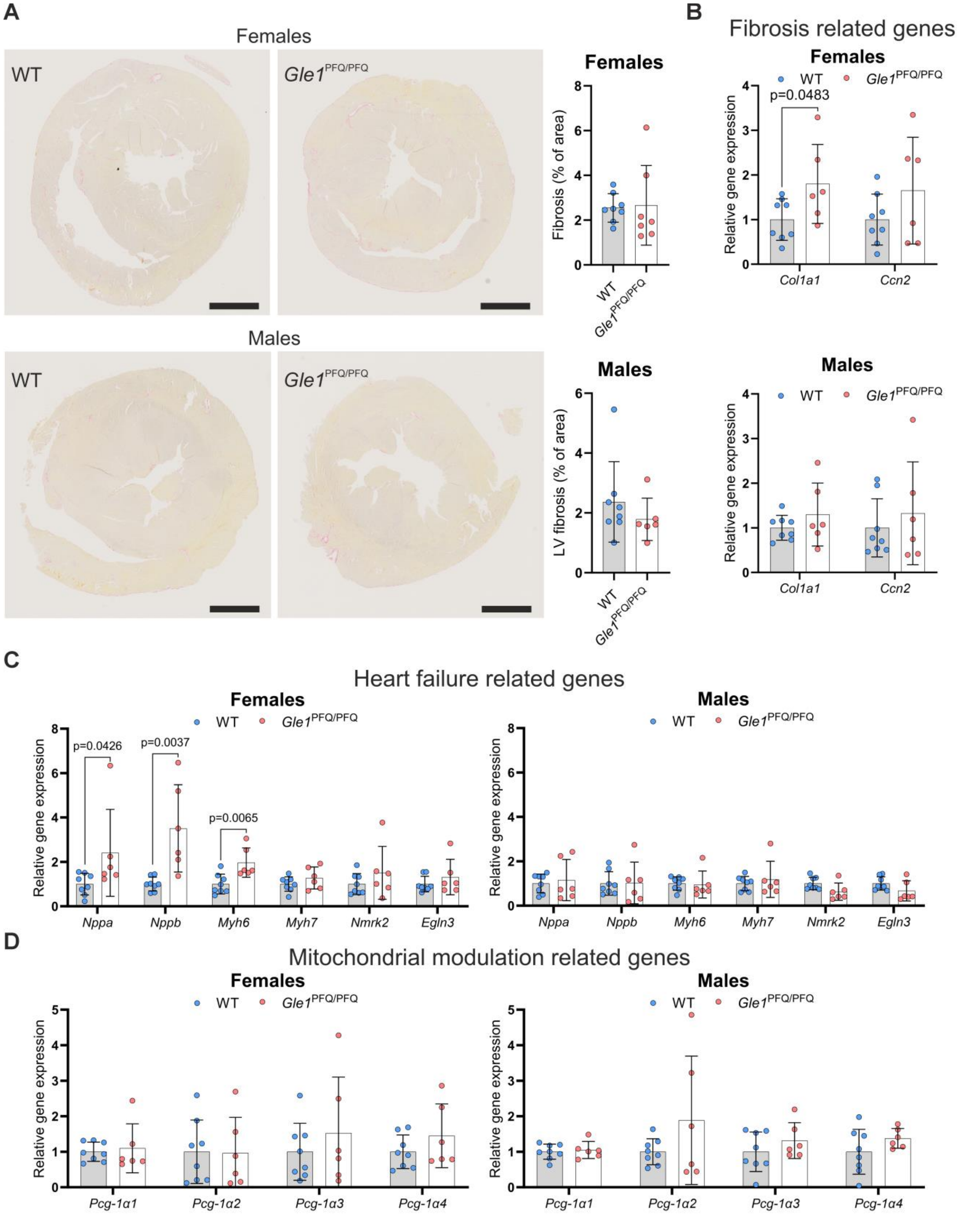

Supplementary figure 16: Molecular analysis of the *Gle1*$^{PFQ/PFQ}$ adult hearts

A) Adult heart (17-25 weeks) sections stained by picrosirius red to visualize the level of fibrosis. Means ± SD are plotted, as well as individual data points generated from each mouse (WT females: 8, KI females: 7, WT males: 8, KI males: 6). Scale bar 1 mm.

B-D) RT-qPCR analysis of adult hearts (17-25 weeks), focusing on transcripts related to **B)** fibrosis, **C)** heart failure, and **D)** mitochondrial modulation. Means ± SD are plotted, as well as individual data points generated from each mouse (WT females: 8, *Gle1*$^{PFQ/PFQ}$ females: 6, WT males: 8, *Gle1*$^{PFQ/PFQ}$ males: 6). Two-tailed Student's *t* test.

Supplementary figure 17

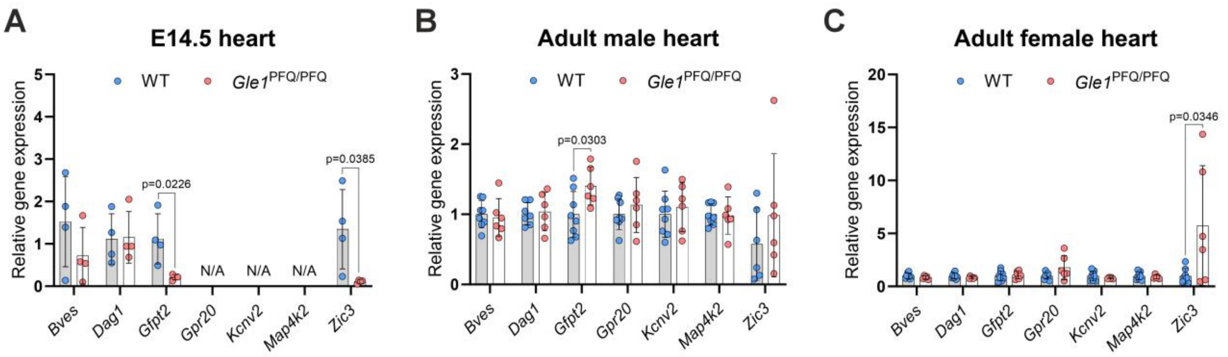

Supplementary figure 17: Transcriptional analysis of cardiac changes in *Gle1*$^{PFQ/PFQ}$ embryonic and adults hearts

A-C) RT-qPCR analysis of embryonic (**A**), E14.5) and adult (**B, C**), 17-25 weeks) hearts, focusing on differentially expressed transcripts from the *Gle1* KO RNA-seq dataset, which are associated with heart dysfunction based on the literature. Means ± SD are plotted, as well as individual data points generated from each mouse (WT embryos: 4, *Gle1*$^{PFQ/PFQ}$ embryos: 4, WT females: 8, *Gle1*$^{PFQ/PFQ}$ females: 6, WT males: 8, *Gle1*$^{PFQ/PFQ}$ males: 6). Two-tailed Student's *t* test.

Supplementary table 1

Oligomers used for microinjections to modify the *Gle1* gene. The nine nucleotides inserted in the *Gle1* KI line via the ssDNA repair template are highlighted in red.

	**Oligo**	**Sequence**	**Manufacturer**
KO line	crRNA1	TGACATGCCATGCCGTCCGA	IDT
	crRNA2	CTAGGGAGCCTGCTAAACGC	IDT
KI line	crRNA3	CAGGTGCTGACCCTTCTAGG	IDT
	ssDNA	AGCAGATGAGCCAGGTGCTGTGCCACAGGGAAAACA GGTGCTGACCCTTCTAGCCGTTTCAAGAGGGCCTGAG GCAATGGCAGGAGGAGCAAGAGAGGAAGGTGCGAG CCCTCTCAGAGATGGC	IDT

Supplementary table 2

Genotyping and sequencing primers for the Gle1 KO and KI mice

Primer	Sequence	Manufacturer
Gle1 KO DNA Se	CCACGTAGGCTCGCTAGTTT	IDT
Gle1 KO DNA As	AGCTGAAGGCCCAAGCAAAT	IDT
Gle1 KI DNA Se	ACAAGTCAGTGCTTGAATATAGAGC	IDT
Gle1 KI DNA As	AACAGGCACTCAGGAAGACT	IDT
Gle1 KI cDNA Se	GAGTCCCCGTTCACAGAACC	IDT
Gle1 KI cDNA As	GAAGCTTTAGGTTGAGAATCTTGGC	IDT

Supplementary table 3

List of RT-qPCR primers

**Primer**	**Sequence**	**Manufacturer**
Gle1 F	CTTCTTGGAGGTGTGCGGGA	IDT
Gle1 R	TGTCCTGAGCTCGTGATGGC	IDT
Cdkn2a (p16) F	GTGTGCATGACGTGCGGG	IDT
Cdkn2a (p16) R	GCAGTTCGAATCTGCACCGTAG	IDT
Cdkn2a (p19) F	GCTCTGGCTTTCGTGAACAT	IDT
Cdkn2a (p19) R	TCGAATCTGCACCGTAGTTGAG	IDT
Cdkn1a (p21) F	AACATCTCAGGGCCGAAA	IDT
Cdkn1a (p21) R	TGCGCTTGGAGTGATAGAAA	IDT
Il1α F	GCACCTTACACCTACCAGAGT	IDT
Il1α R	AAACTTCTGCCTGACGAGCTT	IDT
Il1β F	TTCAGGCAGGCAGTATCACTC	IDT
Il1β R	GAAGGTCCACGGGAAAGACAC	IDT
Il6 F	TAGTCCTTCCTACCCCAATTTCC	IDT
Il6 R	TTGGTCCTTAGCCACTCCTTC	IDT
Cxcl1 F	AACCGAAGTCATAGCCACAC	IDT
Cxcl1 R	GACACCTTTTAGCATCTTTTGG	IDT
Pai-1 F	TGGGTGGAAAGGCATACCAAA	IDT
Pai-1 R	AAGTAGAGGGCATTCACCAGC	IDT
Actb F	CAGCAAGCAGGAGTACGATG	IDT
Actb R	GGTGTAAAACGCAGCTCAGT	IDT
Ywhae F	CCGAGCGATACGACGAAATG	IDT
Ywhae R	CAGTTTCAACCATTTGCCGG	IDT
Zic3 F	TGCTGCCAGTTCAGGCTATG	IDT
Zic3 R	GCAGAAGGGGTTTTAGTGGTATC	IDT
Map4k2 F	CTTCGAGCTGCTACAGCGT	IDT
Map4k2 R	TGGGTCTAGCTTGACGATCTTC	IDT
Gpr20 F	ATGCCCTCTGCGTTGTCTATG	IDT
Gpr20 R	CACGACCATTAGTGCTTGCC	IDT
Dag1 F	CTTGAGGCGTCCATGCACT	IDT
Dag1 R	GGCAATTAAATCCGTTGGAATGC	IDT
Bves F	CCGTGCCTTCTAATGAGACCA	IDT
Bves R	AGACCACATAAAGGGTACATCCT	IDT
Gfpt2 F	ATGTGCGGAATCTTTGCCTAC	IDT
Gfpt2 R	GTCATAGCCCCTGTACTCCAG	IDT
Kcnv2 F	GGATGAACTGAGCGAGCAACT	IDT
Kcnv2 R	TTGTGGTCCATAGAAGCGCAT	IDT
18s F	CGCCGCTAGAGGTGAAATTC	IDT
18s R	CCAGTCGGCATCGTTTATGG	IDT
Pgc-1α1 F	GGACATGTGCAGCCAAGACTCT	IDT
Pgc-1α1 R	CACTTCAATCCACCCAGAAAGCT	IDT
Pgc-1α2 F	CCACCAGAATGAGTGACATGGA	IDT
Pgc-1α2 R	GTTCAGCAAGATCTGGGCAAA	IDT
Pgc-1α3 F	AAGTGAGTAACCGGAGGCATTC	IDT
Pgc-1α3 R	TTCAGGAAGATCTGGGCAAAGA	IDT
Pgc-1α4 F	TCACACCAAACCCACAGAAA	IDT
Pgc-1α4 R	CTGGAAGATATGGCACAT	IDT
Nmrk2 F	GCCGTATGAGGAATGCAAGC	IDT
Nmrk2 R	GCCATCTAAATAGACCACTTCCACC	IDT

Supplementary table 4

List of TaqMan probes

Primer	Forward	Reverse	Probe
18s	TGGTTGCAAAGCTGAAACTTAAAG	AGTCAAATTAAGCCGCAGGC	CCTGGTGGTGCCCTTCCGTCA
Nppa	GAAAAGCAAACTGAGGGCTCTG	CCTACCCCCGAAGCAGCT	TCGCTGGCCCTCGGAGCCT
Nppb	TGGGCAGAAGATAGACCGGA	ACAACCTCAGCCCGTCACAG	CGGCGCAGTCAGTCGCTTGG
Col1a1	CCCTGGCCTTGGAGGAA	CACGGAAACTCCAGCTGATTTT	CTTTGCTTCCCAGATGTCCTATGGCTATGATG
Ctgf	CGCCAACCGCAAGATTG	CACGGACCCACCGAAGAC	CACTGCCAAAGATGGTGCACCCTG
Myh6	GGTGCCAAGAAGATGCACG	TTATGTTTATTGTGTATTGGCCACAG	CGAGGAATAACCTCTCCAGCAGACCCTC
Myh7	AGCTCTAAGGGTGCCCGTG	TGCTTCCACCTAAAGGGCTG	AGCCCTCAGACCTGGAGCCTTTGC
Egln3	TGAGGCTGGATCTGGAGAAGA	AGCCGACCTCGTGCAGAC	CGCCCTGGAGTACATCGTGCCC

Supplementary table 5

List of antibodies used for Western blotting

Target	Host	ID	Manufacturer	Dilution
GLE1	Rabbit	ab96007	Abcam	1:500
Ubiquitin	Rabbit	3933	Cell signaling	1:1 000
eIF2α	Rabbit	ab26197	Abcam	1:1 000
eIF2α (phospho S51)	Rabbit	ab32157	Abcam	1:500
Anti-rabbit-HRP	Sheep	A16172	Thermo Fisher	1:5 000

Supplementary table 6

List of antibodies and probes used for fluorescent staining

Target	Host	ID	Manufacturer	Dilution
GLE1	Rabbit	ab96007	Abcam	1:500
ZO-1	Mouse	339100	Invitrogen	1:400
Tyrosine hydroxylase	Rabbit	657012	Merck	1:500 (whole-mount)
Tyrosine hydroxylase	Mouse	MAB318	Merck	1:1000 (section)
SOX10	Rabbit	AB155279	Abcam	1:250
PHOX2B	Mouse	sc-376997	Santa Cruz	1:100
G3BP1	Rabbit	13057-2-AP	Thermo Fisher	1:500
HH3 (pSer10)	Rabbit	06-570	Thermo Fisher	1:500
γH2A.X (phospho S139)	Rabbit	ab81299	Abcam	1:200
LAMA1	Rabbit	L9393	Merck	1:1 000
Connexin 43	Rabbit	C6219	Thermo Fisher	1:1 000
N-cadherin	Mouse	MA1-91128	Thermo Fisher	1:400
Sema3A	Goat	sc-1148	Santa Cruz	1:50
Vinculin	Mouse	V9131	Merck	1:500
CDX2	Mouse	CDX2-88	BioGenex	1:500
Cleaved caspase-3	Rabbit	9664	Cell Signalling	1:600
GATA6	Goat	AF1700	R&D Systems	1:150
NANOG	Rabbit	8822	Cell Signalling	1:1 000
OCT3/4	Goat	sc-8628	Santa Cruz	1:400
Isl1/2	Mouse	39.4D5	DSHB	1:100
Sox2	Goat	AF2018	R&D Systems	1:200
Olig2	Rabbit	AB9610	Merck	1:500
TUJ1	Rabbit	AB18207	Abcam	1:1000
Synaptophysin	Rabbit	NB120-16659	Novus Biologicals	1:50
Alexa Fluor® 488 AffiniPure™ Donkey Anti-Goat IgG (H+L)	Donkey	705-546-147	Jackson ImmunoResearch	1:400
Donkey anti-Mouse IgG (H+L) Alexa Fluor™ 568	Donkey	A10037	Thermo Fisher	1:400
Donkey anti-Rabbit IgG (H+L) Alexa Fluor™ Plus 647	Donkey	A32795	Thermo Fisher	1:400
Alexa Fluor™ 488 Phalloidin		A12379	Thermo Fisher	1:600
Griffonia Simplicifolia Lectin I (GSL I) Isolectin B4. Biotinylated		B-1205	Vector laboratories	1:50
Streptavidin / FITC		F0422	DakoCytomation	1:200
α-Bungarotoxin-AF555		B35451	Thermo Fisher	1:500
